# Extracellular matrix proteolysis maintains synapse plasticity during brain development

**DOI:** 10.1038/s41593-025-02153-4

**Published:** 2025-12-22

**Authors:** Haruna Nakajo, Ran Cao, Supriya A. Mula, Justin McKetney, Nicholas J. Silva, Kathy H. Li, Robert J. Chalkley, Lisa K. Randolph, Muskaan Shah, Indigo V. L. Rose, Martin Kampmann, Danielle L. Swaney, Christoph Kirst, Anna V. Molofsky

**Affiliations:** 1 Department of Psychiatry and Behavioral Sciences/Weill Institute for Neurosciences, University of California, San Francisco, San Francisco, CA USA; 2 J. David Gladstone Institutes, San Francisco, CA USA; 3 Quantitative Biosciences Institute (QBI), University of California, San Francisco, San Francisco, CA USA; 4 Department of Cellular and Molecular Pharmacology, University of California, San Francisco, San Francisco, CA USA; 5 Department of Pharmaceutical Chemistry, University of California, San Francisco, San Francisco, CA USA; 6 Institute for Neurodegenerative Diseases, Weill Institute for Neurosciences, University of California, San Francisco, San Francisco, CA USA; 7 Neuroscience Graduate Program, University of California, San Francisco, San Francisco, CA USA; 8 Department of Biochemistry and Biophysics, University of California, San Francisco, San Francisco, CA USA; 9 Department of Anatomy, University of California, San Francisco, San Francisco, CA USA; 10 Data & Computational Science Department, Scientific Data Division, Computing Sciences Area, Lawrence Berkeley National Laboratory, Berkeley, CA USA; 11 Kavli Institute for Fundamental Neuroscience, University of California, San Francisco, San Francisco, CA USA

**Keywords:** Synaptic development, Synaptic plasticity

## Abstract

The extracellular matrix (ECM) regulates synaptic plasticity via mechanisms that are still being defined and have been studied predominantly in adulthood. Here, using live imaging of excitatory synapses in zebrafish hindbrain, we observed a bimodal distribution of short-lived (dynamic) and longer-lived (stable) synapses. Disruption of ECM via digestion or brevican deletion destabilized dynamic synapses and led to decreased synapse density. Conversely, loss of matrix metalloproteinase 14 (MMP14) led to accumulation of brevican and increased the lifetime of the dynamic synapse pool without affecting the stable synapse pool, resulting in increased overall synapse density. Microglial MMP14 was essential to these effects in both fish and human induced pluripotent stem cell-derived cultures. Both MMP14 and brevican were required for experience-dependent synapse plasticity in a motor learning assay. These data, complemented by mathematical modeling, define an essential role of ECM remodeling in maintaining a dynamic subset of synapses during brain development.

## Abstract (web summary)

This study demonstrates that the extracellular matrix (ECM) of the developing brain stabilizes recently born synapses. The authors identify a microglial metalloprotease that digests the ECM to increase the plasticity of these newborn synapses.

## Main

Neuronal synapse numbers increase markedly during brain development and undergo a prolonged period of experience-dependent refinement that shapes adult brain function^1^. In humans, synapses increase throughout early childhood and are pruned in adolescence^2,3^ and synapse dysfunction occurs in neurodevelopmental diseases^4,5^. The extracellular matrix (ECM) is a lattice of sugars and glycoproteins that attach to a hyaluronan sugar backbone and fill the extracellular spaces of the brain, which compose up to 20% of brain volume^6^. The ECM is also a critical regulator of synaptic plasticity^7,8^. Much of the evidence for this view derives from studies showing that enzymatic ECM digestion can reopen plasticity in cortical circuits^9,10^, impair learning and memory^11,12^, and promote recovery from central nervous system (CNS) injury^13^. However, ECM protein composition and glycosylation patterns change over the course of development, and some of these changes may be linked to the closure of critical periods^14,15^. Taken together, this suggests that the ECM has a unique molecular composition in development and could have temporally distinct roles.

The abundance and composition of ECM depends on both its structural components as well as enzymes that digest and remodel it. Many structural ECM proteins impact CNS synaptic function, including chondroitin sulfate proteoglycans (CSPGs) such as aggrecan and brevican^9,16–18^, link proteins^19–21^ and tenascins^22,23^, which collectively assemble onto the hyaluronan backbone in the brain’s extracellular space. However, the endogenous mechanisms that remodel the ECM are less understood. ECM remodeling enzymes include matrix metalloproteinases of the MMP, ADAM and ADAMTS families^24,25^. Among these is the matrix metalloproteinase MMP14, which is among a small number of MMPs that are membrane bound, potentially enabling ECM remodeling in a cell contact-dependent manner.

Microglia, the innate immune cells of the CNS parenchyma, promote ECM remodeling^26–30^. Live imaging studies show that microglial contact induces spine formation and spine plasticity^31,32^, mirroring the effect of localized ECM digestion^9^. However, studies of synapse, ECM and microglial dynamics in the intact developing brain have been limited by imaging constraints in developing mammals. In contrast, zebrafish (*Danio rerio*) is a vertebrate model system that develops ex utero and is transparent up to 14 days post fertilization (dpf), enabling dynamic imaging of core neurodevelopmental processes^33,34^. We recently characterized a population of microglia in the zebrafish hindbrain that interact with synapses and recapitulate core morphological and molecular features of mammalian microglia^35^, facilitating studies of microglia–synapse interactions during development.

Here, using time-lapse live imaging in the developing zebrafish hindbrain, we show that the structural ECM protein brevican and the matrix metalloproteinase MMP14 are key regulators of developmental synapse plasticity. We identify two pools of excitatory synapses with distinct kinetics, and demonstrate that ECM modifications are selectively required for the maintenance of newborn, but not long-lived, synapses. Our data suggest that a key function of ECM remodeling during development is to maintain a dynamic pool of synapses to enable experience-dependent adaptation.

## Results

### Characterization of synapse dynamics and ECM in the developing brain

**Fig. 1:**
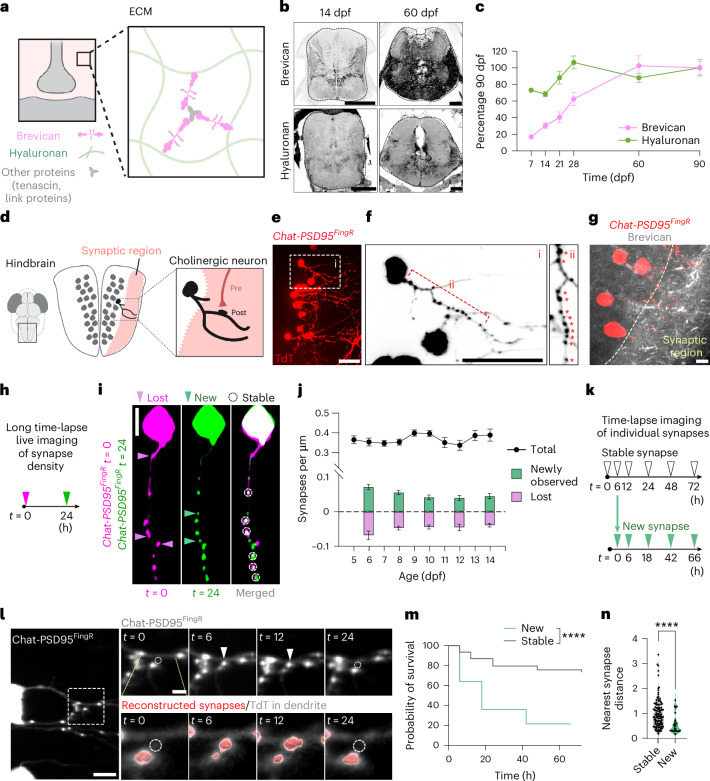
Characterization of synapse dynamics and ECM in the developing brain. **a**, Schematic of the perisynaptic ECM which consists of a hyaluronan sugar matrix connected by proteoglycans including brevican, linking proteins such as tenascins and others. **b**, Representative images of zebrafish hindbrain at 14 and 60 dpf showing brevican (antibody staining) and hyaluronan labeled with a genetically encoded sensor, *ubi:ssncan-GFP*. **c**, Quantification of fluorescence intensity of brevican and hyaluronan (*ubi:ssncan-GFP*) over development from 7 to 90 dpf. Mean fluorescence intensities for brevican and hyaluronan were normalized to the intensity at 90 dpf. Brevican quantification, number of fish: 7 dpf, *n* = 9; 14 dpf, *n* = 8; 21 dpf, *n* = 8; 28 dpf, *n* = 7; 60 dpf, *n* = 7; 90 dpf, *n* = 6. For hyaluronan, number of fish: 7 dpf, *n* = 11; 14 dpf, *n* = 10; 21 dpf, *n* = 6; 28 dpf, *n* = 10; 60 dpf, *n* = 7; 90 dpf, *n* = 3. **d**, Schematic of zebrafish larval hindbrain. Gray dots indicate cell bodies and the synaptic region is shown in pink. A cholinergic neuron with a cell body and dendrites is shown in black. **e**, Dorsal view of zebrafish hindbrain at 10 dpf shows sparsely labeled cholinergic neurons expressing a TdT-tagged FingR construct that binds to the excitatory post-synaptic marker PSD95. *Tg(chata:gal4);Tg(zcUAS:PSD95.FingR-TdT-CCR5TC-KRAB(A))*, hereafter abbreviated *Chat-PSD95*^*FingR*^. **f**, Strategy for quantification of synapses using *Chat-PSD95*^*FingR*^. Insets of region in **e** show (i) several sparsely labeled neurons and (ii) a dendritic segment from one cholinergic neuron with synapses indicated by asterisks. **g**, Immunostaining for brevican protein and *Chat-PSD95*^*FingR*^ at 14 dpf. Synaptic region is indicated in the image. **h**, Schematic of 24-h time-lapse imaging assay to quantify changes of synapse density. **i**, Representative images show a single *Chat-PSD95*^*FingR*^ dendrite imaged at 7 dpf (*t* = 0) and 8 dpf (*t* = 24). Pink arrowheads: synapses present at *t* = 0 and absent at *t* = 24, ‘lost synapses’. Green arrowheads: synapses that appear at *t* = 24, ‘new synapses’. White dashed circles: present at both time points. **j**, Quantification of total excitatory synapse density and dynamics over the live imaging window of hindbrain development (5–14 dpf), based on *Chat-PSD95*^*FingR−TdT*^ puncta normalized to µm of dendrite length. Black line indicates static synapse density per day (*P* = 0.3437, one-way ANOVA). Lilac bars indicate lost synapses (**F_(4,78)_ *P* = 0.0040, one-way ANOVA). Green bars indicate new synapses (*F_(4,78)_ *P* = 0.018, one-way ANOVA). Asterisks in the figure represent results of Tukey’s multiple comparisons with respect to 5–6 dpf. Number of fish: 5–6 dpf, *n* = 15; 7–8 dpf, *n* = 18; 9–10 dpf, *n* = 18; 11–12 dpf, *n* = 15; 13–14 dpf, *n* = 17. Results from individual fish are shown in Extended Data Fig. 2b. **k**, Schematic of time-lapse imaging to determine the fate of individual synapses at the indicated time points. Synapses at *t* = 0 were defined as ‘stable’ synapses. Synapses born between *t* = 0 and *t* = 6 were defined as ‘new’ synapses and subsequently followed with the 6-h time point set as *t* = 0 (lower time course). Experiments were performed at 10–12 dpf. **l**, Representative image of a single excitatory synapse imaged at *t* = 0, 6, 12 and 24 shows a ‘new synapse’ born between *t* = 0 and *t* = 6, which disappeared between *t* = 12 and *t* = 24. Left: a low-power image of a *Chat-PSD95*^*FingR*^ cholinergic neuron. Dashed square indicates inset. Inset shows raw fluorescence (top) and fluorescence overlaid with 3D reconstruction of synapses (bottom). Arrowheads: newborn synapse. Dashed circle: site of newborn synapse. **m**, Kaplan–Meier plot of survival of individual synapses over time (data from *n* = 19 fish, *n* = 427 stable synapses and *n* = 25 new synapses, ****P < 0.0001, log-rank test). **n**, Quantification of the distance from the nearest synapse for stable and new synapses at *t* = 6 (stable, *n* = 116 inter-synapse intervals from *n* = 14 fish; new, *n* = 25 inter-synapse intervals from *n* = 14 fish; ****P < 0.0001, Welch’s *t*-test, performed for synapses). The inter-synapse intervals were normalized by the mean of stable synapses for each cell. Values were plotted as mean ± s.e.m. ****P < 0.0001; **P < 0.01; *P < 0.05. For representative images in **e**, **f** and **g**, similar results were observed from more than *n* = 3 independent experiments. Scale bars, 100 µm (**b**), 20 µm (**e**,**f**), 5 µm (**g**,**i**,**l** (low-power image)), 2 µm (**l**, inset). NS, not significant. Illustrations in **a** and **d** created using BioRender.com. Source data

**Extended Data Fig. 1:**
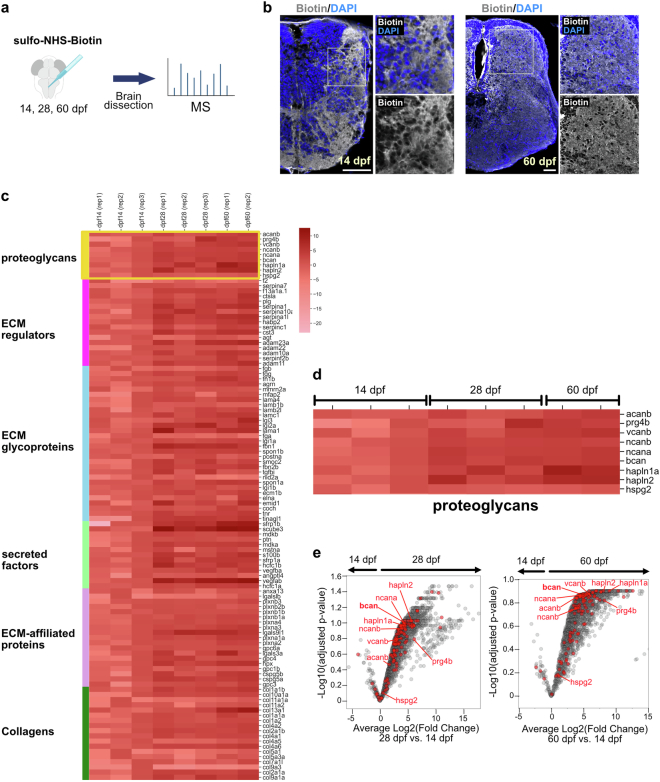
Characterization of ECM over development in hindbrain. **a**) Schematics of proteomics analysis in zebrafish brains. **b**) Representative images of biotinylation of the extracellular space for mass spectrometry at 14 and 60 dpf. Sulfo-NHS-Biotin were detected by Streptoavidin conjugated with fluorophores. Squares indicate inset on the right. Scales: 100 µm. Similar result was observed in n = 2 individual experiments. **c**) Heat map of the extracellular proteome detected by proteomics analysis at 14, 28, 60 dpf filtered by proteins in the matrisome database^41^ (n = 3 replicates at 14 and 28 dpf, n = 2 replicates at 60 dpf). **d**) Proteoglycans from the heatmap in c. **e**) Volcano plots of proteins comparing 14 dpf vs. 28 dpf (left) and 14 dpf vs. 60 dpf (right). The log fold change and adjusted p value are shown.

**Extended Data Fig. 2:**
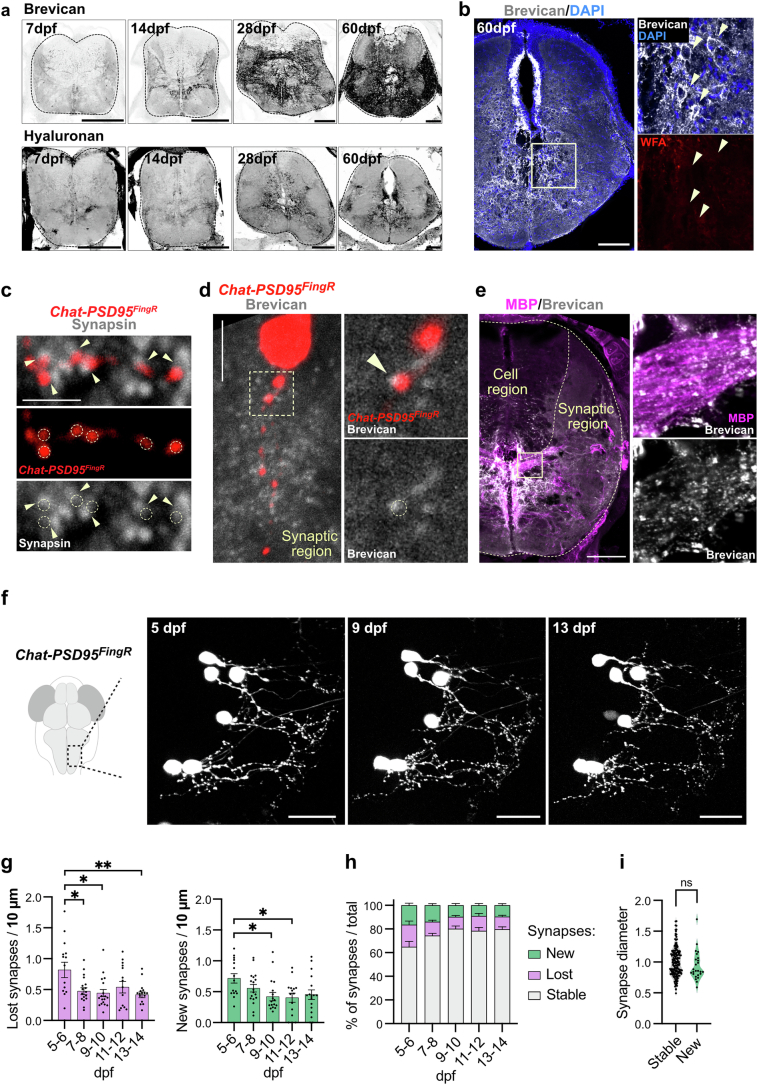
Further assessment of ECM and synapse dynamics in the developing hindbrain. **a**) Representative images of brevican and hyaluronan (*ubi:ssncan-GFP*) in the hindbrain at 7, 14, 28, and 60 dpf. Scales: 100 µm. **b**) Representative image of PNN (perineuronal net)-like brevican staining at 60 dpf. Square indicates the inset on the right. Arrowheads indicate PNN-like brevican signals. Scale: 100 µm. **c**) Representative images of *Chat-PSD95*^*FingR*^ puncta co-stained with presynaptic protein synapsin. Dashed circles indicate *Chat-PSD95*^*FingR*^ puncta and arrowheads indicate colocalization with synapsin. Scale: 2 µm. **d**) Representative images of co-staining for brevican and *Chat-PSD95*^*FingR*^ at 14 dpf. Dashed box indicates inset on the right. Arrowhead indicates association between brevican and *Chat-PSD95*^*FingR*^ puncta. Dashed circle indicates location of *Chat-PSD95*^*FingR*^ puncta. Scale: 5 µm. **e**) Representative images of co-staining for brevican and MBP (myelin basic protein) at 14 dpf hindbrain. Dashed line indicates the hindbrain region. Square indicates inset on the right. Scale: 50 µm. **f**) Representative images of *Chat-PSD95*^*FingR*^ at 5, 9, and 13 dpf from the hindbrain of the same fish. Scales: 20 µm. **g**) Plots of lost (left, pink) and new (right, green) synapses for individual fish from Fig. 1j. Individual dots indicate the value for each fish (5-6 dpf, n = 15 fish; 7-8 dpf, n = 18 fish; 9-10 dpf, n = 18 fish; 11-12 dpf, n = 15 fish; 13-14 dpf, n = 17 fish). Asterisks in the figure represent results of Tukey’s multiple comparisons with respect to the 5-6 dpf shown in Fig. 1j. **h**) Quantification of percentage of new, lost, and stable synapses in each measured time point of Fig. 1j. Total number of synapses were calculated as the sum of the number of newly observed, lost and stable synapses. **i**) Quantification of synapse diameter from stable synapses and new synapses at t = 6 (Stable, n = 157 synapses from n = 14 fish; New, n = 25 synapses from n = 14 fish; p = 0.206, Welch’s t-test, performed for synapses). The synapse diameters were normalized by the mean value of stable synapses for each cell. Values were plotted as mean ± SEM. *: p < 0.05; **: p < 0.01; ns: not significant. For the representative images, similar results were observed in at least n = 2 experiments. Source data

The brain’s perisynaptic ECM includes a diverse protein scaffold (Fig. 1a). We characterized the extracellular proteome of the developing zebrafish by biotinylation and mapping to the zebrafish matrisome^36^ (Extended Data Fig. 1a,b). Most ECM proteins increased over developmental time (14–60 dpf), including CSPGs (Extended Data Fig. 1c–e and Supplementary Tables 1 and 2). For further validation in situ, we focused on brevican, an abundant and brain-specific CSPG that plays important roles in synapse development and plasticity^16,37^. We quantified brevican and hyaluronan in the hindbrain region^35^ between 7 and 90 dpf, spanning the equivalent of mammalian embryonic development through adulthood. Brevican was quantified by immunostaining and hyaluronan was visualized using the reporter fish *Tg(ubi:ssncan-GFP)*^38^ (Fig. 1b and Extended Data Fig. 2a). Hyaluronan was mostly stable over development, whereas brevican increased progressively until 60 dpf (Fig. 1c), consistent with studies in rodents^39,40^. In mammals, dense ECM structures around neurons called perineuronal nets are thought to regulate synapse plasticity in adult brain^41^. Adult zebrafish also have net-like brevican structures that were not observed in development (Extended Data Fig. 2b) but did not co-stain with *Wisteria* *floribunda* agglutinin lectin (WFA), a marker of perineuronal nets used in mammals. These results suggest a distinct composition and structure of the developing brain ECM compared with adulthood.

We next established tools to examine synapses in developing hindbrain, focusing on cholinergic neurons expressing the choline acetyltransferase gene *chata*^42^ (Fig. 1d). To visualize excitatory synapses onto cholinergic neurons, we used a transgenic system based on FingR technology (fibronectin intrabodies generated by messenger RNA display), which can report on endogenous synaptic proteins^43,44^. We established a transgenic (Tg) line expressing FingR targeted to post-synaptic density protein 95 (PSD95) using the GAL4/UAS system to sparsely label neurons^42–44^ (Fig. 1e,f). This transgenic fish *Tg(chata:gal4);Tg(zcUAS:PSD95.FingR-TdT-CCR5TC-KRAB(A))* is hereafter referred to as *Chat-PSD95*^*FingR*^ (Fig. 1f). Chat-PSD95^FingR^ puncta colocalized with the presynaptic protein synapsin (Extended Data Fig. 2c). Brevican diffusely surrounded neurons and their synapses in the developing hindbrain (Fig. 1g), although we sometimes observed punctate brevican near synapses (Extended Data Fig. 2d). We also observed brevican near axons, consistent with previous studies^45^ (Extended Data Fig. 2e). Synapses are dynamic during early development. Here, we quantified excitatory synapse numbers using single images from fixed or live-imaged brains as shown in Fig. 1f. To quantify synapse dynamics, we imaged the same neuron at two time points (*t* = 0 and *t* = 24 h; Fig. 1h). Using these two approaches, we could quantify both synapse density as well as synapses that formed or were removed during the time window (Fig. 1i).

We first quantified total synapse density and dynamics between 5 and 14 dpf. We found that excitatory synapse density was largely stable over this imaging period (Fig. 1j, black line). Neuronal dendrite length and branching was also stable (Extended Data Fig. 2f). However, time-lapse imaging showed that 24 ± 1% of synapses were replaced every 24 h (Fig. 1j and Extended Data Fig. 2g,h). We termed these ‘newly observed’ and ‘lost’ synapses. The term ‘newly observed’ was meant to clarify that these numbers reflected an average over a 24-h window, and might not capture more transient ‘new’ synapses, which are examined in more detail below. While synapse turnover was mildly decreased at later time points, overall, our data suggest a remarkable amount of synaptic turnover throughout this developmental window.

Studies suggest that while some synapses are stable over the life of the organism^46,47^, others live only a few hours^34^. We tracked individual synapses by serial time-lapse imaging over 72 h. Synapses present at the start of the imaging window (*t* = 0) were defined as ‘stable’, whereas synapses that appeared between *t* = 0 and *t* = 6 were defined as ‘new’ (Fig. 1k). We found that 74% of stable synapses were still present at *t* = 72. In contrast, less than 20% of new synapses survived to the end of the imaging window (Fig. 1l,m). New synapses tended to form in proximity to other synapses (Fig. 1n), reminiscent of findings in adult mouse^48^. PSD95 diameter was not different (Extended Data Fig. 2i). These findings suggest a majority population of stable synapses and a smaller proportion of synapses that live only a few hours.

### ECM depletion preferentially destabilized newborn synapses

**Fig. 2:**
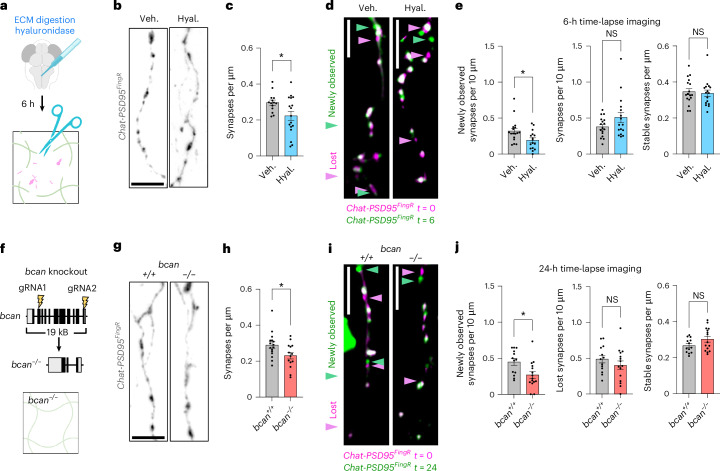
ECM depletion preferentially destabilized newborn synapses. **a**, Schematic of strategy for ECM digestion by hyaluronidase injection into the hindbrain ventricle. Tissues fixed or imaged 6 h after injection with hyaluronidase (Hyal.) or vehicle (Veh.) (PBS). **b**, Representative images of *Chat-PSD95*^*FingR*^ dendrites at 14 dpf from fixed section stained for ΤdT after vehicle or hyaluronidase injection. **c**, Quantification of synapse density at 14 dpf (Chat-PSD95^FingR^ puncta) per µm of dendrite length. Dots represent means per fish, with *n* = 1–4 dendritic segments quantified per fish (vehicle, *n* = 19 dendrites from *n* = 14 fish; hyaluronidase, *n* = 25 dendrites from *n* = 17 fish; *P = 0.020, Welch’s *t*-test for fish). **d**, Representative merged images of *Chat-PSD95*^*FingR*^ collected before injection (*t* = 0, pink) and 6 h after (*t* = 6, green) hyaluronidase or vehicle injection. Overlap of pink and green appears white. Pink arrowheads, lost synapses; green arrowheads, newly observed synapses. Experiment performed at 10–12 dpf. Nonmerged images are shown in Extended Data Fig. 3h. **e**, Quantification of newly observed, lost or stable synapses between *t* = 0 and *t* = 6 after hyaluronidase versus vehicle injection (vehicle, *n* = 17 fish; hyaluronidase, *n* = 16 fish; *P = 0.017 for newly observed, *P* = 0.117 for lost, *P* = 0.722 for stable, Welch’s *t*-test). **f**, Schematic of generation of brevican knockout (*bcan*^*−/−*^) fish by CRISPR genome editing. gRNAs targeting exon 3 and exon 14 were injected to delete 19 kilobases of the *bcan* gene. The truncation resulted in loss of *bcan* mRNA and brevican protein (Extended Data Fig. 3e,f). **g**, Representative images of *Chat-PSD95*^*FingR*^ dendrites at 14 dpf from fixed section stained for ΤdT from *bcan*^*+/+*^ versus *bcan*^*−/−*^ fish. **h**, Quantification of synapse density at 14 dpf (Chat-PSD95^FingR^ puncta) per µm of dendrite length. Dots represent means per fish, with *n* = 1–3 dendritic segments quantified per fish (*bcan*^*+/+*^, *n* = 23 dendrites from *n* = 16 fish; *bcan*^*−/−*^, *n* = 17 dendrites from *n* = 14 fish; *P = 0.031, Welch’s *t*-test for fish). **i**, Representative merged images of *Chat-PSD95*^*FingR*^ collected from *bcan*^*+/+*^ and *bcan*^*−/−*^ at *t* = 0 (pink) and *t* = 24 (green). Pink arrowheads, lost synapses; green arrowheads, new synapses. Experiment performed at 10–12 dpf. Nonmerged images are shown in Extended Data Fig. 3i. **j**, Quantification of newly observed, lost and stable synapses between *t* = 0 and *t* = 24, *bcan*^*+/+*^ versus *bcan*^*−/−*^ fish (*bcan*^*+/+*^, *n* = 13 fish; *bcan*^*−/−*^, *n* = 15 fish; *P = 0.010 for newly observed, *P* = 0.288 for lost, *P* = 0.085 for stable, Welch’s *t*-test). Values were plotted as mean ± s.e.m. *P < 0.05. Scale bars, 5 µm (**b**,**d**,**g**,**i**). Illustration in **a** created using BioRender.com. Source data

**Extended Data Fig. 3:**
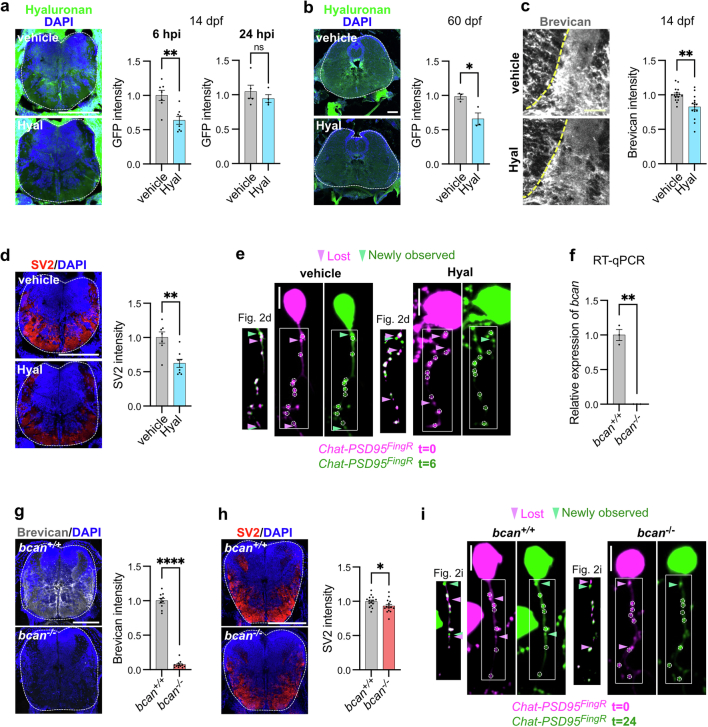
Optimization and validation of ECM digestion and *bcan* deletion. **a**) Representative images and quantification of hyaluronan depletion after hyaluronidase injection at 14 dpf, measured as mean fluorescence intensity of GFP (*ubi:ssncan-GFP*) at 6 h post injection (hpi) (vehicle, n = 7 fish; Hyal, n = 8 fish; p = 0.0023**, Welch’s t-test) and 24 hpi (vehicle, n = 5 fish; Hyal, n = 4 fish; p = 0.392, Welch’s t-test). GFP intensity normalized to mean of vehicle control. Dashed lines indicate hindbrain regions. Scale: 100 µm. **b**) Representative images and quantification of hyaluronan depletion at 6 hpi at 60 dpf, measured as mean fluorescence intensity of GFP (*ubi:ssncan-GFP*) nomalized to vehicle control (vehicle, n = 3 fish; Hyal, n = 3 fish; p = 0.049*, Welch’s t-test). Scale: 100 µm. **c**) Representative images and quantification of brevican depletion at 6 h after hyaluronidase injection at 14 dpf (vehicle, n = 16 fish; Hyal, n = 15 fish; p = 0.0018**, Welch’s t-test). Brevican intensity normalized to mean of vehicle control. Dashed lines indicate hindbrain regions. Scale: 20 µm. **d**) Representative images and quantification of SV2 presynapse marker 6 h after hyaluronidase injection at 14 dpf (vehicle, n = 7 fish; Hyal, n = 8 fish; p = 0.003**, Welch’s t-test). SV2 intensity normalized to mean of vehicle control. Scale: 100 µm. **e**) Non-merged images of time lapse imaging from Fig. 2d. Circles indicate stable synapses. Boxes indicate the region shown in Fig. 2d. Scale: 5 µm. **f**) Expression of *bcan* gene in *bcan*^*−/−*^ fish assessed by RT-qPCR from whole larvae. Expression normalized to *ef1a* housekeeper gene (n = 3/group; p = 0.0064**, Welch’s t-test). **g**) Representative images and quantification of brevican protein in *bcan*^*−/−*^ fish (*bcan*^*+/+*^, n = 10 fish; *bcan*^*−/−*^, n = 12 fish; p < 0.0001****, Welch’s t-test). Brevican intensity normalized to mean of *bcan*^*+/+*^ control. Scale: 100 µm. **h**) Representative images and quantification of SV2 presynapse marker in *bcan*^*−/−*^ fish at 14 dpf (*bcan*^*+/+*^, n = 15 fish; *bcan*^*−/−*^, n = 15 fish, p = 0.049*, Welch’s t-test). SV2 intensity normalized to mean of *bcan*^*+/+*^ control. Scale: 100 µm. **i**) Non-merged images of time lapse imaging experiment from Fig. 2i. Circles indicate stable synapses. Boxes indicate the region shown in Fig. 2i. Scale: 5 µm. Values were plotted as mean ± SEM. ****: p < 0.0001; **: p < 0.01; *: p < 0.05; ns: not significant. Source data

To define how ECM impacts developing synapses, we examined both static synapse numbers and synapse dynamics using two independent approaches to deplete the ECM. First, we injected hyaluronidase (Fig. 2a) and analyzed 6 h later to capture the maximal reduction in ECM density (Extended Data Fig. 3a–c). This acute ECM depletion reduced excitatory synapse density at 14 dpf (Fig. 2b,c and Extended Data Fig. 3d), and led to fewer newly observed synapses relative to vehicle control, whereas the numbers of lost and stable synapses were unchanged (Fig. 2e and Extended Data Fig. 3e).

**Extended Data Fig. 4:**
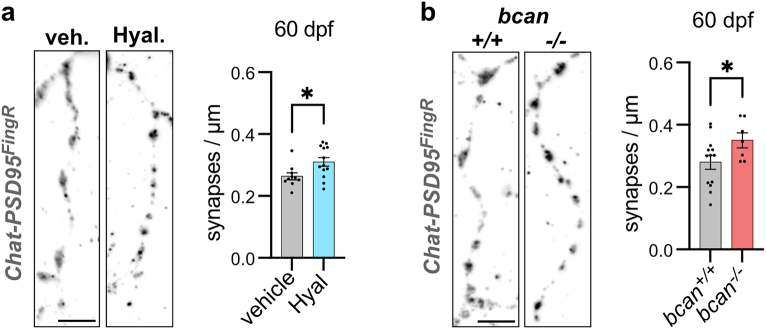
ECM depletion increases synapse density in the adult brain. **a**) Representative images and quantification of *Chat-PSD95*^*FingR*^ at 60 dpf from fixed section stained for ΤdT six hours after vehicle (PBS) or hyaluronidase injection. Dots represent means per fish, with n = 1-4 dendritic segments quantified per fish (vehicle, n = 16 dendrites from n = 10 fish; Hyal, n = 20 dendrites from n = 13 fish; p = 0.018*, Welch’s t-test for fish). Scale: 5 µm. **b**) Representative images and quantification of *Chat-PSD95*^*FingR*^ dendrites at 60 dpf from fixed section stained for ΤdT from *bcan*^*+/+*^vs. *bcan*^*−/−*^ fish. Dots represent means per fish, with n = 1 ~ 3 dendritic segments quantified per fish (*bcan*^*+/+*^, n = 25 dendrites from n = 13 fish, *bcan*^*−/−*^, n = 11 dendrites from n = 7 fish; p = 0.048*, Welch’s t-test for fish). Scale: 5 µm. Values were plotted as mean ± SEM. *: p < 0.05. Source data

We next generated brevican knockout (*bcan*^*−/−*^) fish (Fig. 2f and Extended Data Fig. 3f,g). These fish also had lower excitatory synapse density relative to *bcan*^*+/+*^ controls (Fig. 2g,h and Extended Data Fig. 3h), consistent with findings in juvenile mouse hippocampus^16^. Time-lapse imaging revealed fewer newly observed synapses, but no changes in the lost or stable synapse pools (Fig. 2i,j and Extended Data Fig. 3i). Interestingly, ECM depletion had the opposite effect in adult fish (60 dpf): both hyaluronidase and brevican knockout increased synapse density (Extended Data Fig. 4). These data suggest that the ECM may differentially impact developing versus adult synapses. In development, loss of ECM preferentially depletes short-lived synapses, contributing to synaptic density reductions.

### The microglial metalloproteinase Mmp14b restricted ECM accumulation and destabilized newborn synapses

**Extended Data Fig. 5:**
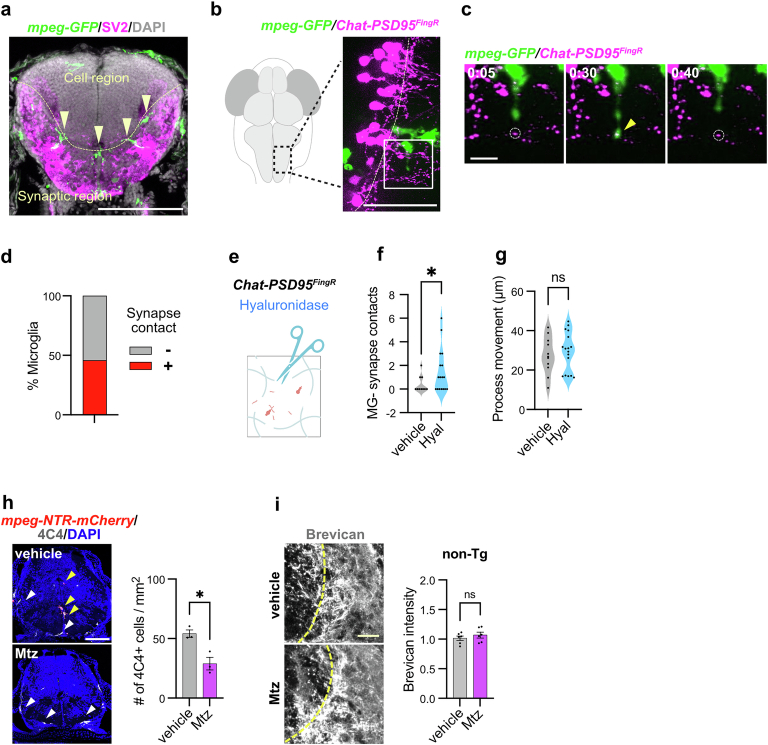
Analysis of microglial contact with excitatory synapses and validation of microglia ablation. **a**) Representative image of microglia and SV2 presynaptic marker in the coronal hindbrain section from *mpeg-GFP* fish at 14 dpf. The DAPI-rich region (cell region) and synapse-rich region (synaptic region) are indicated. Arrowheads indicate microglia associated with the synaptic region. Similar result was observed from more than n = 2 individual experiments. Scale bar, 100 µm. **b**) Schematic of live imaging shows a dorsal view of hindbrain with microglia labeled with *mpeg-GFP*, and sparsely labeled cholinergic neuron and synapses with *Chat-PSD95*^*FingR*^. Similar result was observed from n = 3 individual experiments. Scale: 50 µm. **c**) Representative time lapse image of microglia contact with *Chat-PSD95*^*FingR*^ puncta. Z-stack images taken every 5 min for 1 hr. Dashed circles indicate *Chat-PSD95*^*FingR*^ puncta before and after microglial contact and yellow arrowhead indicates contact detected by colocalization of TdT and GFP. Scale: 10 µm. See also Supplementary video 1. **d**) Quantification of percentage of microglia that contacted *Chat-PSD95*^*FingR*^ puncta during 1 hr video (n = 24 microglia from 13 fish). **e**) Schematic of hyaluronidase injection to digest ECM before live imaging of microglial-synapse contacts. **f**) Quantification of the number of microglial contacts with *Chat-PSD95*^*FingR*^ puncta during 1 hr video (vehicle, n = 10 microglia from 5 fish; Hyal, n = 17 microglia from 8 fish; p = 0.043*, Welch’s t-test, performed for microglia). **g**) Quantification of microglial process movement during 1 hr video (vehicle, n = 11 microglia; Hyal, n = 17 microglia; p = 0.606, Welch’s t-test, performed for microglia). **h**) Representative images of mCherry and the microglial marker 4C4 and quantification of microglia number in the *Tg(mpeg:gal4);Tg(UAS:NTR-mCherry)* with Mtz or vehicle (DMSO) treatment at 14 dpf (vehicle, n = 3 fish; Mtz, n = 3 fish; p = 0.015*, Welch’s t-test). non-Tg= non-transgenic fish. Scale: 100 µm. **i**) Representative images and quantification of brevican staining in Mtz-treated non-transgenic siblings at 14 dpf (vehicle, n = 6 fish; Mtz, n = 6 fish; p = 0.412, Welch’s t-test). Brevican intensity normalized to the mean of vehicle control. Scale: 20 µm. Values were plotted as mean ± SEM.*: p < 0.05; ns: not significant. Source data

We previously showed that microglia promote synapse plasticity in the adult mouse brain by restricting accumulation of ECM^26^. To visualize microglia–synapse interactions we crossed our *Chat-PSD95*^*FingR*^ fish to a microglial/myeloid reporter line (*Tg(mpeg:GFP-CAAX)*, hereafter ‘*mpeg-GFP*’). We observed synapse-adjacent microglia^35^ (Extended Data Fig. 5a,b) that frequently contacted synapses (10–12 dpf; Extended Data Fig. 5c,d and Supplementary Video 1). Of 53 contacts examined, none were associated with subsequent disappearance of the synapse within the 1-h time window. ECM digestion with hyaluronidase modestly increased the frequency of synaptic contacts without altering overall process motility (Extended Data Fig. 5e–g). Thus, microglia contact excitatory synapses in the developing brain.

**Fig. 3:**
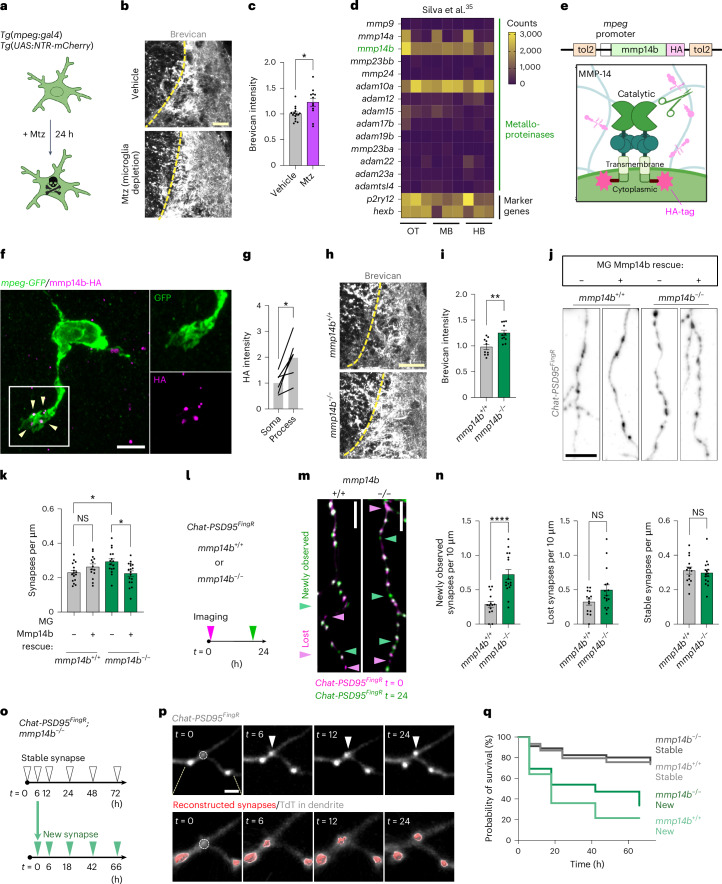
The microglial metalloproteinase Mmp14b restricted ECM accumulation and destabilized newborn synapses. **a**, Schematic of microglial ablation by adding Mtz to fish water in fish that express nitroreductase (*ntr*) in microglia and macrophages (*Tg(mpeg:gal4);Tg(UAS:NTR-mCherry)*). **b**, Representative images of brevican in synaptic region after vehicle (DMSO) or microglial ablation with 5 mM Mtz in fish water for 24 h. Experiment at 14 dpf. **c**, Quantification of brevican intensity in synaptic region normalized to mean of vehicle control (vehicle, *n* = 15 fish; Mtz, *n* = 12 fish; *P = 0.043, Welch’s *t*-test). **d**, Heatmap of absolute expression (counts) of *mmp*, *adam* and *adamts* metalloproteinase genes in zebrafish microglia from bulk RNA sequencing at 28 dpf in optic tectum (OT), midbrain (MB) and hindbrain (HB). Marker genes *hexb* and *p2ry12* are shown for comparison. Reanalyzed from ref. ^35^. **e**, Schematic of a dimer of the membrane-associated metalloproteinase MMP14 and design of the *mmp14b-HA* expression construct for epitope tagging of the C-terminal end with HA. Catalytic domain, transmembrane domain and cytoplasmic domain are indicated. **f**, Mmp14b-HA transgene expression in an *mpeg-GFP* microglia expressing mpeg:mmp14b-HA. Inset shows HA signals colocalized with microglial processes (arrowheads). Similar results were observed from *n* = 2 individual experiments. **g**, Quantification of Mmp14b-HA intensity in microglial processes versus soma. Lines connect data from the same microglia (*n* = 6 microglia from 3 fish; *P = 0.015, paired *t*-test per cell). **h**, Representative images of brevican staining in synaptic regions of *mmp14b*^*−/−*^ and *mmp14b*^*+/+*^ control at 14 dpf. **i**, Quantification of brevican intensity in synaptic region normalized to mean of *mmp14b*^*+/+*^ control (*mmp14b*^*+/+*^, *n* = 10 fish; *mmp14b*^*−/−*^, *n* = 11 fish; **P = 0.0014, Welch’s *t*-test). **j**, Representative images of microglia-specific Mmp14b rescue experiment. *Chat-PSD95*^*FingR*^*;mmp14b*^*+/+*^ and *;mmp14*^*−/−*^ fish were injected with mpeg:mmp14b-HA construct at one-cell-stage embryos and analyzed at 14 dpf. **k**, Quantification of synapse density (Chat-PSD95^FingR^ puncta per µm of dendrite length) in *mmp14b*^*+/+*^ and *mmp14*^*−/−*^ with or without microglial-specific rescue with mpeg:mmp14b-HA. Dots show means per fish from *n* = ~1–4 dendritic segments analyzed per fish (*mmp14*^*+/+*^ no rescue, *n* = 33 dendrites from *n* = 16 fish; *mmp14b*^*+/+*^ rescue, *n* = 31 dendrites from *n* = 14 fish; *mmp14b*^*−/−*^ no rescue, *n* = 27 dendrites from *n* = 16 fish; *mmp14b*^*−/−*^ rescue, *n* = 40 dendrites from *n* = 18 fish; two-way ANOVA for fish, **F_(1,60)_ interaction effect *P* = 0.0022, asterisks showing Tukey’s multiple comparisons). MG, microglia. **l**, Schematic of time-lapse imaging to measure synapse turnover in *Chat-PSD95*^*FingR*^*;mmp14b*^*+/+*^ and *;mmp14b*^*−/−*^ fish. **m**, Representative merged images of synapses in *Chat-PSD95*^*FingR*^*;mmp14b*^*−/−*^ and *;mmp14b*^*+/+*^ fish at *t* = 0 (pink) and *t* = 24 (green). Experiments at 10–12 dpf. Pink arrowheads, lost synapses; green arrowheads, newly observed synapses. Nonmerged images are shown in Extended Data Fig. 8d. **n**, Quantification of newly observed, lost and stable synapses between *t* = 0 and *t* = 24 in *mmp14b*^*+/+*^ versus *mmp14b*^*−/−*^ fish (*mmp14b*^*+/+*^, *n* = 14 fish; *mmp14b*^*−/−*^, *n* = 16 fish; ****P < 0.0001 for newly observed, *P* = 0.058 for lost, *P* = 0.61 for stable, Welch’s *t*-test). **o**, Schematic of time-lapse imaging to determine the fate of individual synapses in *mmp14b*^*−/−*^ fish. Synapses at *t* = 0 were defined as ‘stable’ synapses. Synapses born between *t* = 0 and *t* = 6 were defined as ‘new’ synapses and subsequently followed with the 6-h time point set as *t* = 0 (lower time course). Experiments performed at 10–12 dpf. **p**, Representative image of a single excitatory synapse imaged at *t* = 0, 6, 12 and 24 shows a ‘new synapse’ born between *t* = 0 and *t* = 6 in *mmp14b*^*−/−*^ fish. Raw fluorescence images on top and fluorescence overlaid with 3D reconstruction of synapse on bottom. Arrowheads, newborn synapse. Dashed circle, site of newborn synapse. **q**, Kaplan–Meier plot of survival of individual synapses over time in *mmp14b*^*+/+*^ control (from Fig. 1m) and *mmp14b*^*−/−*^ fish (for *mmp14b*^*−/−*^, data from *n* = 15 fish, *n* = 331 stable synapses and *n* = 26 new synapses). Values were plotted as mean ± s.e.m. ****P < 0.0001; **P < 0.01; *P < 0.05. Scale bars, 20 µm (**b**,**h**), 5 µm (**f**), 5 µm (**j**,**m**), 2 µm (**p**). Illustrations in **a** and **e** created using BioRender.com. Source data

**Extended Data Fig. 6:**
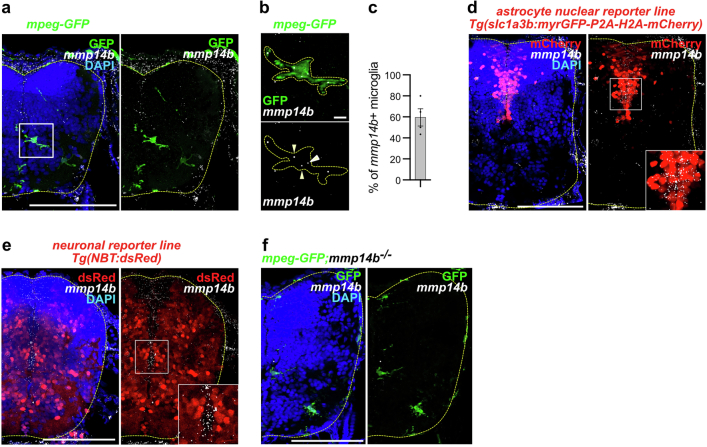
*mmp14b* is expressed in microglia and astrocytes, but not neurons. **a**) Representative image of RNA *in situ* hybridization for *mmp14b* in *mpeg-GFP* fish. Dashed lines indicate hindbrain regions. White box indicates inset in b. Similar result was observed from n = 4 fish from single experiment. Scale: 100 µm. **b**) Inset of microglia in a. Arrowheads indicate *mmp14b* puncta in *mpeg-GFP* microglia. Dashed lines indicate microglia. Scale: 5 µm. **c**) Quantification of percentage of hindbrain microglia expressing *mmp14b*. Dots indicate n = 4 fish. **d**) Representative images of RNA *in situ* hybridization for *mmp14b* in astrocyte reporter line *Tg(slc1a3b:myrGFP-P2A-H2A-mCherry)* at 14 dpf. The mCherry signals indicate the astrocyte nucleus. Dashed lines indicate hindbrain regions. Inset is shown at the bottom. Similar result was observed from n = 3 fish from single experiment. Scale: 100 µm. **e**) Representative images of RNA *in situ* hybridization for *mmp14b* in neuronal reporter line *Tg(NBT:dsRed)* at 14 dpf. Dashed lines indicate hindbrain regions. Inset is shown at the bottom. Similar result was observed from n = 3 fish from single experiment. Scale: 100 µm. **f**) Representative image of RNA *in situ* hybridization for *mmp14b* in *mpeg-GFP*;*mmp14b*^*−/−*^ fish showing lack of probe hybridization. Dashed lines indicate hindbrain regions. Similar result was observed from n = 3 fish from single experiment. Scale: 100 µm. Values were plotted as mean ± SEM.

Conditional partial ablation of microglia (Fig. 3a and Extended Data Fig. 5h,i) led to increased perisynaptic brevican (Fig. 3b,c). These results suggest that microglia restrict ECM accumulation in the developing hindbrain, consistent with their role in the adult brain^26,49^. To determine potential microglial regulators of the ECM, we screened our previous dataset of zebrafish microglia genes^35^ for expression of the major classes of metalloproteinases^24,25,50^. *mmp14b* and *adam10a* were both highly expressed at levels comparable to canonical marker genes (Fig. 3d). Interestingly, both *mmp14b* and *adam10a* are membrane bound, potentially enabling them to cleave proteins in a cell-contact-dependent manner^51,52^. We previously identified mouse *Mmp14* as a top candidate in a screen for ECM remodeling enzymes in mouse microglia^26^. We confirmed *mmp14b* expression in hindbrain microglia by RNA in situ hybridization (Extended Data Fig. 6a–c). *Mmp14b* was also detected in astrocytes but was rarely detected in neurons (Extended Data Fig. 6d,e) and not detected in *mmp14b* knockout fish (*mmp14b*^*−/−*^ (ref. ^53^); Extended Data Fig. 6f). MMP14 can directly cleave ECM proteins and locally activate other metalloproteinases^51^. An epitope-tagged Mmp14b (Fig. 3e) preferentially localized at microglial processes (Fig. 3f,g), suggesting a potential contact-dependent role.

**Extended Data Fig. 7:**
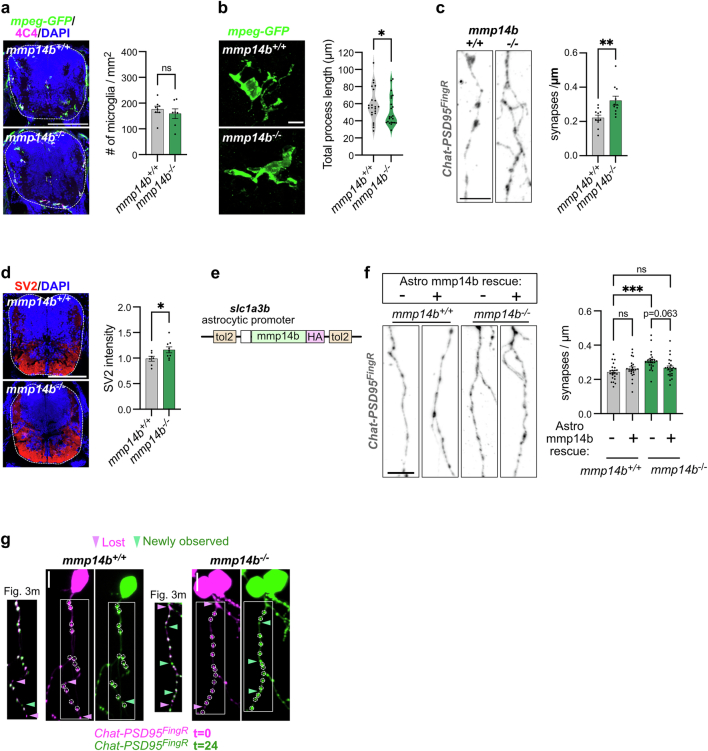
Additional characterization of *mmp14b* deficient fish. **a**) Representative images and quantification of the number of microglia having 4C4 microglial marker in *mpeg-GFP* fish (*mmp14b*^*+/+*^, n = 8 fish; *mmp14b*^*−/−*^, n = 7 fish; p = 0.468, Welch’s t-test). Dashed lines indicate hindbrain regions. Scale: 100 µm. **b**) Representative images and quantification of the total length of microglial processes (*mmp14b*^*+/+*^, n = 23 microglia from n = 7 fish; *mmp14b*^*−/−*^, n = 23 microglia from n = 7 fish; p = 0.026*, Kolmogorov-Smirnov test, performed for microglia). Scale: 5 µm. **c**) Representative images and quantification of synapse density per µm of dendrite length (Chat-PSD95^FingR^ puncta) in *mmp14b*^*−/−*^ and *mmp14b*^*+/+*^ control at 14 dpf. Dots represent means per fish from n = 1-3 dendritic segments analyzed per fish (*mmp14b*^*+/+*^, n = 21 dendrites from n = 11 fish; *mmp14b*^*−/−*^, n = 17 dendrites from n = 11 fish; p = 0.0044**, Welch’s t-test for fish). Scale: 5 µm **d**) Representative images and quantification of SV2 presynapse marker in *mmp14b*^*−/−*^ fish at 14 dpf (*mmp14b*^*+/+*^, n = 7 fish; *mmp14b*^*−/−*^, n = 9 fish, p = 0.035*, Welch’s t-test). SV2 intensity normalized to mean of *mmp14b*^*+/+*^ control. Dashed lines indicate hindbrain regions. Scale: 100 µm. **e**) Schematics of the astrocytic *mmp14b-HA* expression construct. **f**) Representative images and quantification of synapse density per µm of dendrite length (Chat-PSD95^FingR^ puncta) with or without astrocytic Mmp14b rescue. *Chat-PSD95*^*FingR*^*;mmp14b*^*+/+*^ and*;mmp14*^*−/−*^ fish were injected with slc1a3b:mmp14b-HA construct at one-cell stage embryos and analyzed at 14 dpf. Dots show means per fish from n = 1 ~ 4 dendritic segments analyzed per fish (*mmp14b*^*+/+*^ no rescue, n = 47 dendrites from n = 22 fish; *mmp14b*^*+/+*^ rescue, n = 47 dendrites from n = 23 fish; *mmp14*^*−/−*^ no rescue, n = 48 dendrites from n = 24 fish; *mmp14*^*−/−*^ rescue, n = 52 dendrites from n = 24 fish; Two-way ANOVA for fish, F_(1,89)_ interaction effect p = 0.0083**, asterisks show Tukey’s multiple comparisons). Scale: 5 µm. **g**) Non-merged images of time lapse imaging experiment from Fig. 3m. Circles indicate stable synapses. Boxes indicate the region shown in Fig. 3m. Scale: 5 µm. Values were plotted as mean ± SEM.***: p < 0.001; **: p < 0.01; *: p < 0.05; ns: not significant. Source data

To determine the function of Mmp14b in vivo we examined *mmp14b*^*−/−*^ fish^53^ (Extended Data Fig. 6f). Unlike MMP14-deficient mice^54^, fish were viable and fertile with only a small population of adult fish showing skeletal defects^53^. *mmp14b*^*−/−*^ fish had normal numbers of microglia (Extended Data Fig. 7a), but with shorter processes (Extended Data Fig. 7b). *mmp14b*^*−/−*^ fish also had increased brevican (14 dpf; Fig. 3h,i), indicating that Mmp14b restricts ECM accumulation. Mmp14b deletion led to increased excitatory synapses (14 dpf; Extended Data Fig. 7c and Extended Data Fig. 7d), the opposite of what we had observed after ECM depletion (Fig. 2). Microglia-specific rescue of *mmp14b* using the *mpeg:mmp14b-HA* was sufficient to rescue excitatory synapse density to control levels in *mmp14b*^*−/−*^ fish, but did not increase excitatory synapse density in *mmp14b*^*+/+*^ (Fig. 3e,j,k). Astrocyte-specific expression of Mmp14b did not decrease synapse density in *mmp14b*^*−/−*^ fish (Extended Data Fig. 7e,f). Thus, microglial Mmp14b restricts accumulation of ECM and excitatory synapses. To examine the effect of *mmp14b* on synapse dynamics, we performed time-lapse imaging (Fig. 3l). We found that *mmp14b*^*−/−*^ fish had more newly observed synapses (Fig. 3m,n and Extended Data Fig. 7g) and a longer lifetime of newborn synapses (Fig. 3o–q; control data from Fig. 1m). Taken together, these results suggested that microglial Mmp14b restricts ECM and total synapse numbers in the developing brain by preferentially destabilizing newly formed synapses.

### Microglial MMP14 promoted brevican digestion in human cells

**Fig. 4:**
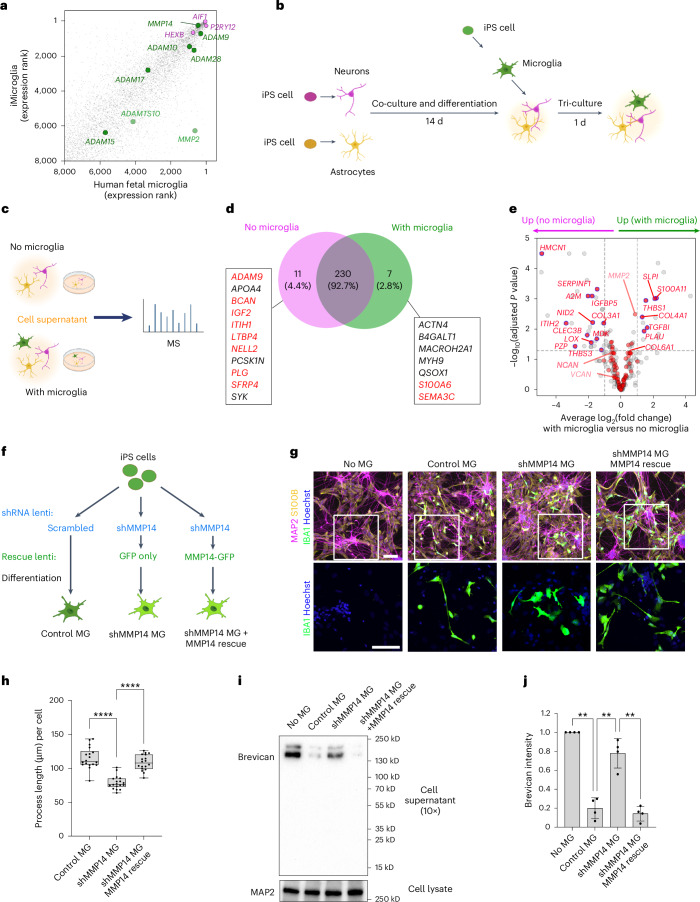
Microglial MMP14 promoted brevican digestion in human cells. **a**, Scatter plot of gene expression (sorted by rank) of iPS-cell-derived microglia and human fetal microglia. MMPs, ADAMs and ADAMTSs families that have transmembrane domain in dark green; MMPs, ADAMs and ADAMTSs without transmembrane domains in light green; microglial marker genes in violet. Data were reanalyzed from the previous study^55^. **b**, Schematic of the iPS-cell-derived astrocyte–neuron–microglia tri-culture system. **c**, Schematic of the proteomics analysis with iPS-cell-derived cells. Cell supernatants were collected from co-culture (neurons and astrocytes; no microglia) and tri-culture (neurons, astrocytes and microglia; with microglia) and analyzed. MS, mass spectrometry. **d**, Venn diagram of proteins detected in cell culture supernatants with or without microglia. Proteins detected only in the absence of microglia are listed on the left and proteins detected only in the presence of microglia are listed on the right. ECM-related proteins as defined by the matrisome database MatrisomeDB2.0^80^ are labeled in red. **e**, Volcano plot of proteins in the intersection of Venn diagram in **d**. The fold change is calculated by comparing the ‘with microglia’ condition with the ‘no microglia’ condition. Thresholds: adjusted *P* < 0.05 (horizontal dashed line), and average log_2_(fold change) > 1 (vertical dashed gray lines). ECM-related proteins as defined by the matrisome database MatrisomeDB2.0^80^ in red. Significant matrisome proteins are outlined in blue. **f**, Strategy for MMP14 knockdown (KD) by shRNA (shRNA interference) with a scrambled shRNA control, and MMP14 rescue by expression of MMP14 fused to GFP, versus a GFP-only control in microglia. The rescue construct has silent mutations in the MMP-GFP to prevent knockdown by shMMP14. All constructs were delivered by lentivirus at the iPS cell stage and used the ubiquitous promoter EF1a. **g**, Representative images of the tri-culture system with MMP14 KD and rescue in microglia. Iba1 (microglia), S100β (astrocyte) and neuron (MAP2) stainings are shown. Scale bar, 100 µm. **h**, Quantification of the process length of microglia in the tri-culture system with MMP14 KD and rescue. Dots show mean microglial process length per field of view from 18 fields of view over 3 independent experiments. Box-and-whiskers plot: box shows 25th to 75th percentile; whiskers show minimum to maximum. Statistics performed on means per field of view (Kruskal–Wallis test; asterisks in the figure show results of Dunn’s multiple comparisons). **i**, Representative image of western blotting for brevican in cell supernatant and MAP2 loading control in cell lysate without microglia or with microglia after MMP14 KD and rescue. **j**, Quantification of brevican in the cell supernatant without microglia or with microglia after MMP14 KD and rescue. Each dot represents mean brevican intensity normalized to no microglia control from *n* = 4 independent experiments (one-way ANOVA; asterisks in the figure show results of Tukey’s multiple comparisons). Values were plotted as mean ± s.e.m. ****P < 0.0001; *P < 0.05. Illustrations in **b**, **c** and **f** created using BioRender.com. Source data

To explore the relevance of this pathway in human cells we established a tri-culture system of neurons, astrocytes and microglia. *MMP14* is highly expressed in human fetal microglia and conserved in microglia from induced pluripotent stem (iPS) cells (Fig. 4a, reanalyzed from ref. ^55^). Human microglia express multiple metalloproteinases, and *MMP14* and *ADAM10* are among the most highly expressed, similar to zebrafish. To determine the impact of microglial MMP14 on the brain ECM, we co-cultured iPS-cell-derived neurons^56^ and astrocytes^57^ for 14 d to generate ECM, then added iPS-cell-derived microglia^58^ for 24 h (Fig. 4b).

Label-free mass spectrometry on cell culture supernatants (Fig. 4c) revealed robust enrichment for ECM and extracellular proteins in the culture supernatant from both conditions, including the CSPGs brevican, neurocan and versican (Fig. 4d,e and Supplementary Tables 3 and 4). Addition of microglia led to a loss of brevican detection (Fig. 4d) although other ECM proteins were not altered (neurocan, versican), or in some cases increased (COL4A1). MMP2, a secreted metalloproteinase that is known to cleave brevican^59^ and can be proteolytically activated by MMP14 (ref. ^51^), was increased. These data demonstrate that microglia alter the extracellular proteome and can reduce brevican levels.

**Extended Data Fig. 8:**
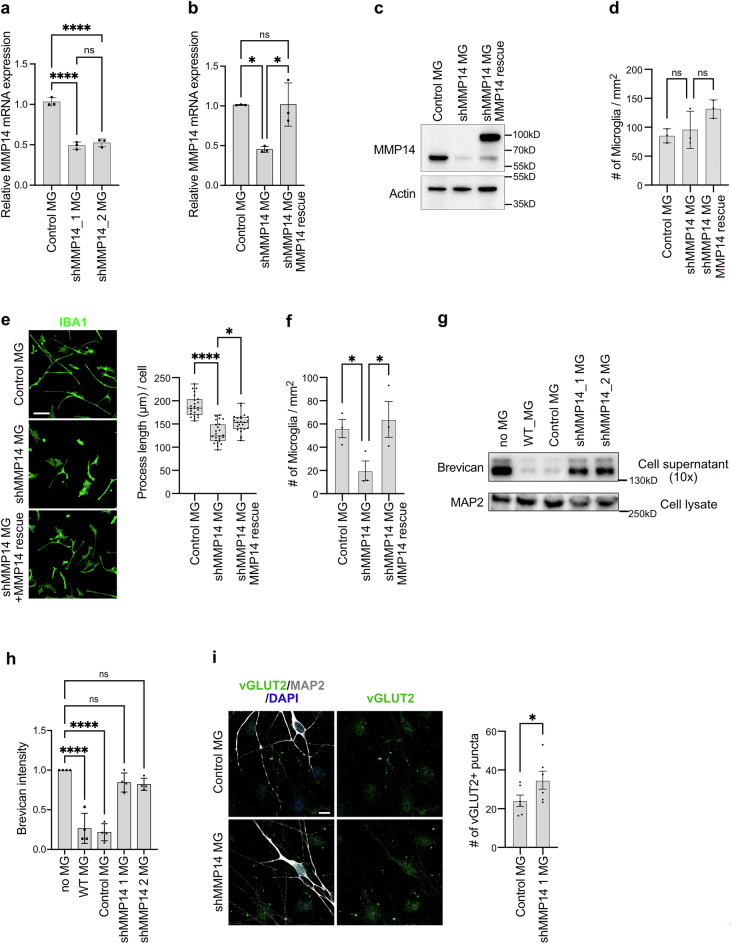
Validation of MMP14 knock-down in human iPSC derived Microglia. **a**) *MMP14* mRNA levels from MMP14 KD microglia analyzed by RT-qPCR showing two independent shRNAs targeting MMP14, with scrambled shRNA as control. Expression normalized to *GAPDH* housekeeping gene (n = 3 independent experiments; One-way ANOVA with Tukey’s multiple comparison). **b**) *MMP14* mRNA levels from MMP14 KD and MMP14-GFP rescue microglia analyzed by RT-qPCR. Expression normalized to *GAPDH* (n = 3 independent experiments; one-way ANOVA, asterisks in the figure show results of Tukey’s multiple comparison). **c**) Western blot of MMP14 showing *MMP14* KD and rescue with MMP14-GFP in microglia. Note higher molecular weight of MMP14-GFP vs. endogenous MMP14. **d**) Quantification of the microglia cell number in the triculture system with MMP14 KD and MMP14-GFP rescue (n = 3 independent experiments, p = 0.209, one-way ANOVA). **e**) Representative images and quantification of process length in the microglia mono-culture system with MMP14 KD and rescue. Dots show mean microglial process length per field of view from 24 fields of view over 3 independent experiments. Box-and-whiskers plot: box shows 25-75th percentile, whiskers show min to max. Statistics performed on means per field of view. (Kruskal-Wallis test, asterisks in the figure show results of Dunn’s multiple comparisons). Scale: 100 µm. **f**) Quantification of the microglia cell number in the microglia mono-culture system with MMP14 KD and MMP14-GFP rescue (n = 3 independent experiments, one-way ANOVA, asterisks in the figure show results of Dunn’s multiple comparison). **g**) Representative western blot of Brevican in cell supernatant vs. MAP2 loading control in cell lysate from the tri-culture system with MMP14 KD. **h**) Quantification of brevican in the cell supernatant in tri-culture system with MMP14 KD. Each dot represents mean brevican intensity normalized to no microglia control from n = 4 independent experiments (n = 4 independent experiments, p < 0.0001****, One-way ANOVA, asterisks in figure show results of Tukey’s multiple comparison). **i**) Representative images and quantification of synapse puncta in the tri-culture system with or without MMP14 KD in microglia. The number of vglut2+ puncta per image field were quantified (n = 6 technical replicates for each group, p = 0.043*, Welch’s t-test). Scale: 10 µm. Values were plotted as mean ± SEM.****: p < 0.0001; *: p < 0.05; ns: not significant. Source data

To determine the impact of microglial MMP14, we reduced MMP14 levels by delivering lentiviruses to express short hairpin RNAs (shRNAs) (Fig. 4f and Extended Data Fig. 8a), and overexpressed an MMP14–GFP fusion protein with synonymous mutations in the *MMP14* shRNA binding region to prevent its repression (Extended Data Fig. 8b,c). Both constructs were delivered to iPS cells before differentiation into iMicroglia (Fig. 4f,g). Knockdown of MMP14 led to smaller, rounder microglia with shorter processes, whereas MMP14 rescue increased process length to control levels (Fig. 4g,h), with no change in microglial numbers (Extended Data Fig. 8d). Interestingly, microglia in monocultures with only Matrigel-derived ECM had substantially longer processes (Extended Data Fig. 8e) and impaired microglial survival, partly or fully rescued by re-expression of MMP14 (Extended Data Fig. 8f). These results suggest that while MMP14 has cell autonomous effects on microglia, these effects can be context-dependent. Brevican reduction after addition of microglia was MMP14-dependent (Fig. 4i,j and Extended Data Fig. 8g,h). These data indicate that microglia promote digestion of brevican via MMP14. Although these cultures had few synapses, vglut2^+^ presynaptic puncta were significantly increased in the tri-culture system after MMP14 knockdown (Extended Data Fig. 8i). Taken together, these data suggest that MMP14 can promote microglial function in human cells as well as in zebrafish, and that microglial MMP14 promotes cleavage and digestion of brevican.

### Brevican and MMP14 regulated neuromotor behaviors

**Fig. 5:**
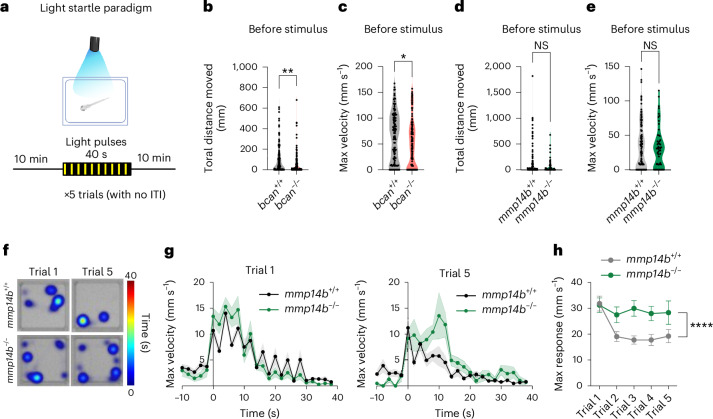
Brevican and MMP14 regulated neuromotor behaviors. **a**, Schematic of light startle paradigm performed in this study. For the behavior assay, 14-dpf pigmented fish were used. ITI, inter-trial interval. **b**, Quantification of total distance moved before light stimulus of Trial 1 in the *bcan*^*−/−*^ versus *bcan*^*+/+*^ control (*bcan*^*+/+*^, *n* = 120 fish; *bcan*^*−/−*^, *n* = 128 fish; **P = 0.011, Welch’s *t*-test). **c**, Quantification of maximum (max) velocity before light stimulus of Trial 1 in the *bcan*^*−/−*^ versus *bcan*^*+/+*^ control (*bcan*^*+/+*^, *n* = 120 fish; *bcan*^*−/−*^, *n* = 128 fish; *P = 0.013, Welch’s *t*-test). **d**, Quantification of total distance moved before light stimulus of Trial 1 in the *mmp14b*^*−/−*^ versus *mmp14b*^*+/+*^ control (*mmp14b*^*+/+*^, *n* = 118 fish; *mmp14b*^*−/−*^, *n* = 52 fish; *P* = 0.072, Welch’s *t*-test). **e**, Quantification of maximum velocity before light stimulus of Trial 1 in the *mmp14b*^*−/−*^ versus *mmp14b*^*+/+*^ control (*mmp14b*^*+/+*^, *n* = 118 fish; *mmp14b*^*−/−*^, *n* = 52 fish; *P* = 0.596, Welch’s *t*-test). **f**, Representative heatmaps of Trial 1 and Trial 5 from *mmp14*^*+/+*^ and *mmp14*^*−/−*^ fish. The heatmap shows the time spent in each area during the light stimulation period. **g**, Maximum velocity for each 2-s time bin was plotted from 10 s before the first light stimulus to the end of the last 2-s dark period. The results from the Trial 1 and Trial 5 are shown (*mmp14b*^*+/+*^, *n* = 115 fish from Trials 1–3, *n* = 75 fish from Trial 4–5; *mmp14b*^*−/−*^, *n* = 50 fish from Trials 1–3, *n* = 27 fish from Trials 4–5). **h**, Quantification of maximum response upon light stimuli for each trial (*mmp14b*^*+/+*^, *n* = 115 fish from Trials 1–3, *n* = 75 fish from Trials 4–5; *mmp14b*^*−/−*^, *n* = 50 fish from Trials 1–3, *n* = 27 fish from Trials 4–5, ****P < 0.0001, two-way ANOVA, *P* value for genotype). Values were plotted as mean ± s.e.m. ****P < 0.0001; **P < 0.01; *P < 0.05. Illustration in **a** created using BioRender.com. Source data

To assess how this pathway impacts physiologic behaviors, we examined startle responses which occur upon sudden visual stimulus but diminish with habituation^60,61^. We used a light startle paradigm to record spontaneous motor activity both before and after a 40-s train of light pulses over five trials (total time 103 min; Fig. 5a). *bcan*^*−/−*^ fish had impaired baseline motility (Fig. 5b,c), precluding further analyses, whereas mmp*14b*^*−/−*^ fish did not (Fig. 5d,e). *mmp14b*^*−/−*^ fish had normal initial startle responses but failed to habituate relative to control fish (Fig. 5f–h). These data implicate brain ECM as a regulator of neuromotor behavior and suggest that Mmp14b-dependent ECM remodeling promotes habituation and neural circuit adaptation to stimuli.

### ECM accumulation blunted experience-dependent synaptic change

**Fig. 6:**
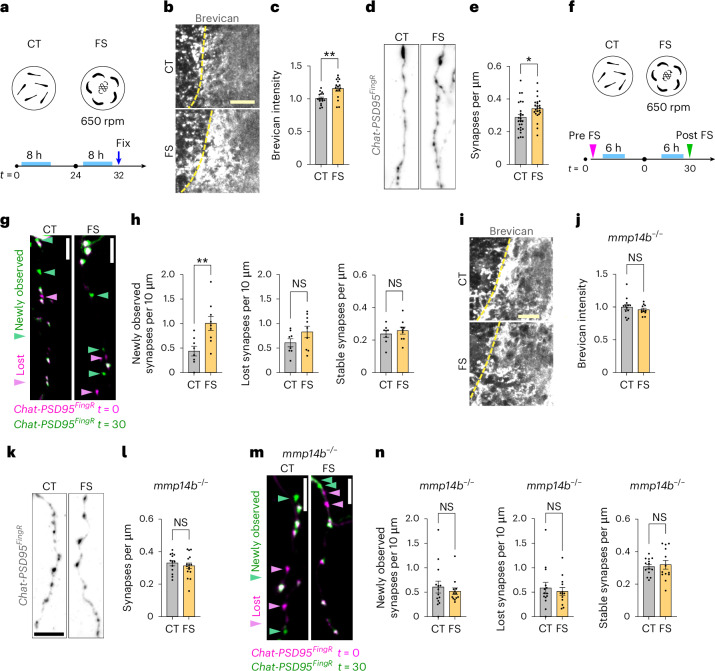
ECM accumulation blunted experience-dependent synaptic change. **a**, Schematic of the forced swim paradigm performed in this study. Forced swim (FS) groups were put on a Petri dish with a stirrer bar and exposed to water current for 8 h per day at 650 rpm, for 2 consecutive days. Control (CT) groups were put in a Petri dish without stirrer bars. Experiments were performed at 13–14 dpf. **b**, Representative images of brevican staining in CT and FS groups. **c**, Quantification of brevican intensity in synaptic region normalized to mean of CT group (CT, *n* = 16 fish; FS, *n* = 16 fish; **P = 0.0021, Welch’s *t*-test). **d**, Representative images of *Chat-PSD95*^*FingR*^ dendrites from fixed sections stained for TdT in FS or CT group. **e**, Quantification of excitatory synapse density (Chat-PSD95^FingR^ puncta) per µm of dendrite length in FS or CT group. Dots represent means per fish from *n* = 1–4 dendritic segments analyzed per fish (CT, *n* = 39 dendrites from *n* = 25 fish; FS, *n* = 34 dendrites from *n* = 23 fish; *P = 0.026, Welch’s *t*-test for fish). **f**, Schematic of time-lapse synapse imaging performed before (*t* = 0) and after (*t* = 30) forced swim paradigm. Experiments were performed at 10–12 dpf. **g**, Representative merged images of time-lapse assay in the *Chat-PSD95*^*FingR*^ before (*t* = 0, pink) and after (*t* = 30, green) forced swim paradigm or control. Pink arrowheads, lost synapses; green arrowheads, new synapses. Nonmerged images are shown in Extended Data Fig. 10a. **h**, Quantification of newly observed, lost and stable synapses after forced swim paradigm (CT, *n* = 8 fish; FS, *n* = 9 fish; **P = 0.0060 for newly observed, *P* = 0.140 for lost, *P* = 0.507 for stable, Welch’s *t*-test). **i**, Representative images of brevican staining in *mmp14b*^*−/−*^ fish from FS or CT group. **j**, Quantification of brevican intensity in synaptic region in *mmp14b*^*−/−*^ fish normalized to CT group (CT, *n* = 13 fish; FS, *n* = 11 fish; *P* = 0.397, Welch’s *t*-test). **k**, Representative images of *Chat-PSD95*^*FingR*^ dendrite in *mmp14b*^*−/−*^ fish from FS or CT group. **l**, Quantification of synapse density (Chat-PSD95^FingR^ puncta) per µm of dendrite length from FS or CT group in *mmp14b*^*−/−*^. Dots represent means per fish from *n* = 1–4 dendritic segments analyzed per fish (CT, *n* = 20 dendrites from *n* = 13 fish; FS, *n* = 27 dendrites from *n* = 16 fish; *P* = 0.48, Welch’s *t*-test for fish). **m**, Representative merged images of time-lapse assay in the *Chat-PSD95*^*FingR*^*;mmp14b*^*−/−*^ fish before (*t* = 0, pink) and after (*t* = 30, green) forced swim paradigm or control. Pink arrowheads, lost synapses; green arrowheads, new synapses. Nonmerged images are shown in Extended Data Fig. 10b. **n**, Quantification of newly observed, lost and stable synapses after forced swim in the *mmp14b*^*−/−*^ fish (CT, *n* = 14 fish; FS, *n* = 13 fish; *P* = 0.542 for newly observed, *P* = 0.621 for lost, *P* = 0.690 for stable, Welch’s *t*-test). Values were plotted as mean ± s.e.m. **P < 0.01; *P < 0.05. Scale bars, 20 µm (**b**,**i**), 5 µm (**d**,**g**,**k**,**m**). Source data

**Extended Data Fig. 9:**
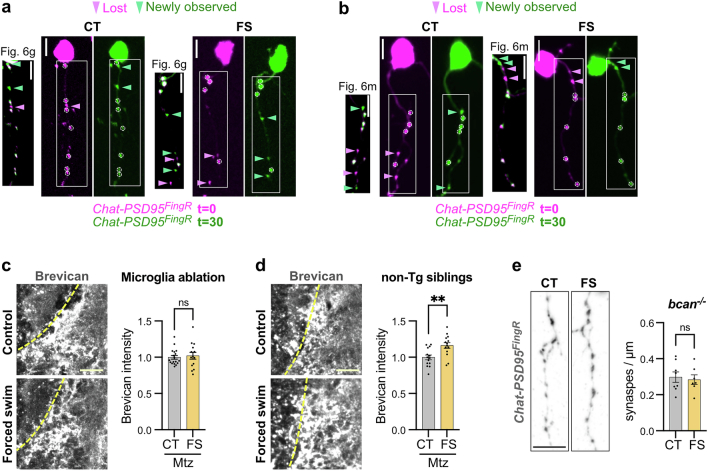
Effect of forced swim paradigm on synapses and brevican. **a**) Non-merged images of time lapse imaging experiment from Fig. 6g. Circles indicate stable synapses. Boxes indicate the region shown in Fig. 6g. Scale: 5 µm. **b**) Non-merged images of time lapse imaging experiment from Fig. 6m. Circles indicate stable synapses. Boxes indicate the region shown in Fig. 6m. Scale: 5 µm. **c**) Representative images and quantification of brevican staining in control and forced swim groups at 14 dpf, with microglia ablation. (CT, n = 18 fish; Mtz, n = 14 fish; p = 0.706, Welch’s t-test). *Tg(mpeg:gal4);Tg(UAS:NTR-mCherry)* fish was treated with 5 mM Mtz during the forced swim paradigm. Brevican intensity normalized to the mean of the control group. Scale: 20 µm. **d**) Representative images and quantification of brevican staining in control and forced swim groups at 14 dpf, without microglia ablation. (CT, n = 14 fish; Mtz, n = 14 fish; p = 0.004**, Welch’s t-test). Non-transgenic siblings were treated with 5 mM Mtz during the forced swim paradigm. Brevican intensity normalized to the mean of the control group. Scale: 20 µm. **e**) Representative images and quantification of synapse density per µm of dendrite length (Chat-PSD95^FingR^ puncta) in *bcan*^*−/−*^ fish from forced swim and control group at 14 dpf. Dots represent means per fish from n = 1-3 dendritic segments analyzed per fish (CT, n = 10 dendrites from n = 8 fish; FS, n = 10 dendrites from n = 7 fish; p = 0.732, Welch’s t-test for fish). Scale: 5 µm. Values were plotted as mean ± SEM. **: p < 0.01; ns: not significant. Source data

Synapses remodel in response to experience. To examine experience-dependent remodeling we used a forced swim paradigm, a stressor that induces plasticity^62,63^, similar to motor learning assays in mice^47^. We performed 8 h of forced swim daily for 2 consecutive days (Fig. 6a). Forced swim increased brevican intensity (Fig. 6b,c) as well as excitatory synapses (Fig. 6d,e). It also increased the number of newly observed synapses without altering stable synapses (Fig. 6f–h and Extended Data Fig. 9a). However, *mmp14b*^*−/−*^ fish had no increase in brevican (Fig. 6i,j) or synapses, although synapses were higher at baseline as previously shown (Fig. 6k,l). Time-lapse imaging did not reveal the increase in newly observed synapses (Fig. 6m,n and Extended Data Fig. 9b). Microglia-ablated fish also did not have more brevican after forced swim (Extended Data Fig. 9c,d), and *bcan*^*−/−*^ fish did not have increased synapses (Extended Data Fig. 9e). Taken together, these results suggest that both stable amounts of ECM (brevican) and the capacity for ECM remodeling are needed for excitatory synapse formation in this model of neural circuit plasticity.

### Computational modeling supported the existence of two synapse states and a role of ECM in stabilizing newborn synapses

**Fig. 7:**
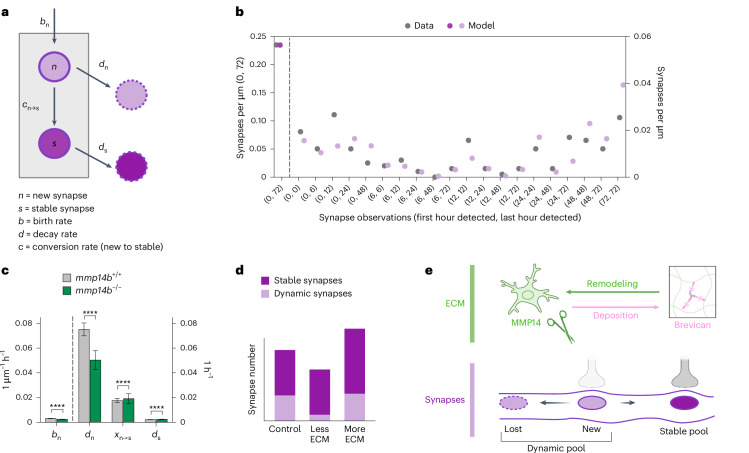
Computational modeling supported the existence of two synapse states and a role of ECM in stabilizing newborn synapses. **a**, Model of synapse dynamics in our system, based on four parameters: birth rate of new synapses (*b*_n_), decay rates of new synapses (*d*_n_), probability of transition from new to stable synapse (*c*_n→s_) and decay rate of stable (*d*_s_) synapses. **b**, Fitting of synapse lifetime data in Fig. 1m from *Chat-PSD95*^*FingR*^ fish, based on the two-state model described in **a**. Densities of synapses for each category of lifetime are shown from raw data (gray) and as predicted by the two-state model (purple). The numbers in parentheses on the *x* axis indicate the first and last time (in hours) that synapses were detected over 72 h. Note that while experimental analyses in Figs. 1 and 3 tracked only synapses that were present at 0 h (‘stable’) or born between 0 and 6 h (‘new’), the computational model examined all synapses observed over 72 h of imaging to generate this predicted distribution. **c**, Synapse dynamics calculated from the two-state model in *mmp14*^*+/+*^ and *mmp14b*^*−/−*^ fish, showing calculations of the four parameters described in **a**. Units for synapse birth rate (*b*_n_) reflect synapses born per µm per hour, shortened to 1 µm^−1^ h^−^^1^. Birth rate is independent of the number of synapses in the system. Units for rates of synapse decay or stabilization (*d*_n_, *c*_n→s_ and *d*_s_) reflect the rates as a proportion of existing synapses, that is, changed synapses per µm per hour divided by total synapses per µm, shortened to 1 h^−1^. Error bars show ±s.d. ****P < 0.0001, Mann–Whitney *U*-test. See Supplementary Data 1 for exact *P* values. **d**, Graphical data summary. **e**, Graphical abstract of ECM impact on synapse numbers and dynamics. Illustrations in **a**, **d** and **e** created using BioRender.com.

Our data on synapse dynamics using ECM loss- and gain-of-function perturbations suggest that ECM stabilizes newborn synapses, thereby increasing synapse number during development. However, as we cannot monitor synapses continuously, our data are limited by the experimentally determined imaging windows. We therefore sought to develop a mathematical model of developmental synapse dynamics that could potentially predict the rate at which synapses are formed and remodeled. Based on our experimental observations, we considered a model whereby developing synapses can exist in two states: a newborn unstable state and a stable state (Fig. 7a; see Supplementary Data 1 for mathematical derivations). Four parameters define the behavior of this model: (1) the synapse birth rate, assumed to be via a homogenous Poisson process (*b*_n_); (2) the probability that a synapse will convert from the unstable to stable state (*c*_n→s_); (3) the decay rate from the newborn state (*d*_n_); and (4) the decay rate from the stable state (*d*_s_). We further assume that each newly born synapse will transition to a stable state or decay with constant rates, and that stable synapses decay but at a slower rate. The total numbers of new and stable synapses are thus random variables that evolve according to the above stochastic dynamics and can be predicted using our model.

**Extended Data Fig. 10:**
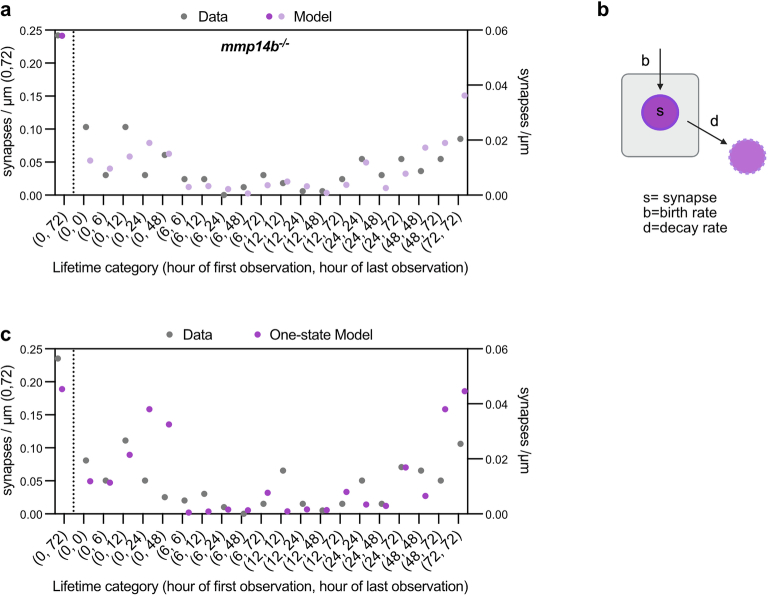
Additional computational modeling of synapse dynamics. **a**) Fitting of synapse lifetime data in Fig. 3q from *mmp14b*^*−/−*^*;Chat-PSD95*^*FingR*^ fish, based on the two-state model in Fig. 7a. Density of synapses for each category of lifetime are shown from raw data and as predicted by the two-state model. The numbers in parentheses indicate the time point of first and last observation of synapses during the time course over 72 h. **b**) Schematic of a “one state” model of synapse dynamics.**c**) Attempted fit of experimental data from control fish in Fig. 1m to a “one state” model shows poor alignment. Compare also Supplemental File 1 for a detailed comparison of the model fits. The numbers in parentheses indicate the time point of first and last observation of synapses during a time course over 72 h.

To test how this two-state model aligned with our experimentally determined data, we used the model to predict synaptic observations made during a 72-h imaging period and compared this model against our experimentally determined synapse lifetime data from Fig. 1k–m. We found that the model accurately predicted the ratio of dynamic to stable synapses observed experimentally in control data from Fig. 1m (Fig. 7b) and in data from *mmp14b*^*−/−*^ fish in Fig. 3q (Extended Data Fig. 10a). In contrast, a single-state model of synapse dynamics was unable to fit the experimentally observed data (Extended Data Fig. 10b,c).

We next used this model to calculate synapse birth rate, decay rates and conversion rates from dynamic to stable in control and *mmp14b*-deficient conditions (Fig. 7c). Examining only our control data, our model predicted that the decay rate (*d*_n_) of newborn synapses is much larger than the decay rate of stable synapses, as expected. It further predicts that most new synapses will decay, rather than be converted to stable synapses. In the setting of *mmp14b* deficiency, the model predicted a 25% reduction in the synapse birth rate, suggesting that a change in birth rate could not explain the increased number of ‘newly observed’ synapses seen over a 24-h imaging window (Fig. 3n). However, the decay rate of new synapses (*d*_n_) was decreased by 33%, and the transition of new to stable synapses (*c*_n→s_) was increased by 9%. Taken together, these computations supported our hypothesis that remodeling of the ECM maintains synapses in a dynamic state and that impaired ECM remodeling leads to an accumulation of stable synapses (Fig. 7d,e). This would be predicted to diminish the efficiency of experience-dependent adaptation and could contribute to the observed circuit and behavioral impacts of ECM alterations.

## Discussion

Our study directly examined how alterations of the brain ECM shape the structural plasticity of developing synapses and identified brevican and microglial MMP14 as a molecular regulator of this process. We propose a model whereby MMP14 acts by promoting microglial cleavage of brevican, which destabilizes and preferentially eliminates recently born synapses. In support of this model, we show that loss of either microglia or MMP14 in developing fish leads to accumulation of brevican, an increase in synapses and an increase in newly stabilized synapses. Conversely, loss of brevican leads to a loss of synapses, consistent with studies in mice^16^, and a decrease in newly stabilized synapses. We show both in fish and in human tri-cultures that microglial MMP14 mediates this effect. It is possible that MMP14 acts on additional ECM proteins besides brevican, or has alternate functions besides ECM cleavage.

For example, the behavioral deficits in brevican-deficient fish are not just the converse of MMP14 deficiency. While this could be explained by the total loss of brevican in the knockout, which is more profound than the 20% increase seen in MMP14-deficient mice, it is also likely that additional ECM proteins are cleaved by MMP14^64^. These could be applicable not only to microglia–synapse interactions, but also to microglial regulation of myelin and other structural components in the CNS. Indeed, brevican is also strongly associated with axons, and MMP14-dependent remodeling of these tracts might also be involved in synapse regulation through influencing neural activity. It is also likely that MMP14 regulation of the ECM has signaling as well as structural roles. For example, MMP14 can act as an ectodomain sheddase^65^, a process that can control the abundance or activity of membrane proteins^66^. Defining how different cell types employ MMP14 is also an interesting direction for future research. Microglia’s high motility and dynamic synaptic contacts may enable them to be a versatile source of Mmp14 to regulate synapse dynamics. Astrocytes make stable, lifelong contact with synapses, as well as axons and blood vessels, and thus astrocyte MMP14 expression may impact other aspects of synapse function. In both cases, it is interesting to speculate that MMP14 regulates process formation and extension and may regulate astrocyte or microglial morphology.

Our data suggest a mechanism through which microglia promote developmental synapse elimination: by destabilizing newborn synapses and promoting their subsequent loss via neuron autonomous mechanisms. Many studies suggest that molecular pathways that promote microglial function also restrict synapse numbers in the developing brain^67–69^. While this is often interpreted as evidence of microglial synapse pruning, or engulfment, a close review of the literature has not identified instances of microglia directly engulfing synapses in vivo^70,71^. This mechanism is distinct from a prevailing model whereby microglia prune synapses by active synaptic engulfment. In this alternate model, we propose that microglia alter the extracellular environment of synapses in a manner that promotes their subsequent elimination or resorption by the neuron.

It is possible that microglial ECM alterations occur in a spatially restricted manner. For example, we observed that microglial processes frequently contacted excitatory synapses, similar to mice^32^. It is interesting to speculate that microglial cleavage of the ECM could be contact-dependent, as MMP14 is surface bound and was concentrated in microglial processes. Interestingly, inhibition of another membrane-bound metalloproteinase, Adam10, in microglia prevented synapse elimination after whisker deprivation in developing mouse sensory cortex^72^. However, support for our model of microglial contact-dependent synapse elimination by ECM clearance would require further experiments to determine (1) whether synapses previously contacted by microglia are eventually eliminated, and (2) whether this effect depends on the amount of ECM in the local environment. Advances in noninvasive microscopy, such as lattice light sheet imaging, could enable prolonged live imaging of structural synaptic changes, which can occur on the order of hours to days^46^, and, ideally, concurrent imaging of the ECM itself. Whether synapse-specific or not, microglial remodeling of the ECM could explain many scenarios in which microglia contribute to synapse elimination.

These findings also raise a critical question: if microglial remodeling of the ECM in development leads to synapse elimination, why does it have the opposite effect of promoting synapse formation in adulthood, as we and others have previously shown^26,73,74^? This is not simply a difference in model systems, as we shown here that two independent methods of ECM depletion reduced synapse numbers in development but increased them in adulthood in the same circuit and synapse type. The answer to this question may reflect both developmental differences in the ECM and differences in the structural plasticity of synapses. With regard to the ECM, we and others have shown a rapid accumulation of ECM proteins including brevican in the early postnatal period. These proteins reach maximal levels and change their glycosylation patterns by adulthood^15,39^. ECM can both stabilize synapses and prevent synaptic plasticity. In development, a relatively looser ECM may stabilize newborn synapses but be easily displaced as new synapses are formed. In adulthood, a denser ECM lattice may form a steric barrier to new synapse formation. Further studies and computational modeling of how ECM function changes with age could extend these findings to other contexts, such as to explain the relationship between ECM accumulation and the closure of developmental critical periods.

A second fundamental difference between development and adulthood is the higher fraction of developing synapses that undergo structural plasticity. While synapse and synaptic protein turnover persists well into adulthood^75^, the fraction of synapses that are dynamic decreases^47^. Our data suggest that most excitatory synapses are short-lived in developing cholinergic neurons in zebrafish hindbrain, consistent with other studies^34^. Increased ECM preferentially increased the number of these nascent synapses, while having relatively little effect on the stable synapse pool. Our data suggest that similar to other synaptic organizers such as neurexins^76^, this effect of the ECM reflects increased stabilization of newborn synapses rather than a direct induction of new synapse formation. Therefore, loss of ECM would preferentially reduce overall synapse numbers in developing brains despite increasing synaptic plasticity in both settings. As zebrafish lose optical transparency around 14 dpf, live imaging in other model organisms (such as the related fish species *Danionella*^77^) could directly test this hypothesis in the same circuit and brain region.

Finally, structural synaptic plasticity is required for experience-dependent adaptation both during development and in adulthood. Our data suggest that ECM remodeling is essential for this plasticity. We found that forced swim, a stressor and inducer of motor learning, led to a robust increase in newborn synapses, consistent with studies of motor learning in mouse cortical neurons^47^. This increase was completely abrogated in MMP14-deficient fish. Chronic stress models in rodents cause an increase of brevican and decrease of MMPs^78,79^, suggesting that both ECM deposition and remodeling could be altered. Thus, ECM remodeling could be essential to optimize circuit function in response to stress and novel experience. Given that MMP14 is highly expressed in human microglia, this mechanism of ECM remodeling may be conserved in the human brain and may suggest new strategies to regulate plasticity in human neurodevelopmental disorders.

## Methods

### Zebrafish

All animal protocols were approved by and in accordance with the ethical guidelines established by the University of California, San Francisco Institutional Animal Care and Use Committee and Laboratory Animal Resource Center. Zebrafish (*D. rerio*) were propagated, maintained and housed in recirculating habitats at 28.5 °C and on a 14/10-h light/dark cycle. Embryos were collected after natural spawns and incubated at 28.5 °C. Sex was not considered as a variable before 28 dpf as this is before sex determination. Adults of mixed sex were used at ages 60 dpf and 90 dpf. Ages were matched within experiments. All experiments were performed with fish on the nonpigmented Casper background (*roy*^*−/−*^*;nacre*^*−/−*^)^81^ except for behavior analyses, which were performed on the normally pigmented Ekkwill (EKW; ZFIN ID: ZDB-GENO-990520-2) background to preserve normal light startle responses. For experiments, fish were assigned to each experimental group in a completely random way.

### Transgenic zebrafish

The following transgenic (Tg) fish described in previous studies were used in this study^38,42,82–84^: *Tg(mpeg1.1:GFP-CAAX)*^zh901Tg^, *Tg(mpeg1.1:gal4)*^zf2055Tg^, *Tg(UAS:NTR-mCherry)*^c264tg^, *Tg(chata:gal4)*^mpn202Tg^, *Tg(ubi:ssncan-GFP)*^uq25bhTg^, *Tg(slc1a3b:myrGFP-P2A-H2A-mCherry)*^vo80Tg^, *Tg(NBT:dsRed)*^zf148Tg^. The following transgenic line was established in this study from a construct previously reported^43^: *Tg(zcUAS:PSD95.FingR-TdT-CCR5TC-KRAB(A))*, and when crossed to a *Tg(chata:gal4)*^mpn202Tg^ this was abbreviated as *Chat-PSD95*^*FingR*^ in the manuscript.

### Cloning

The *Tg(zcUAS:PSD95.FingR-TdT-CCR5TC-KRAB(A))* construct generated in this study was modified from a pTol2-zcUAS:PSD95.FingR-eGFP-CCR5TC-KRAB(A) construct kindly provided by J. Bonkowsky^43^. The pTol2-zcUAS:PSD95.FingR-TdT-CCR5TC-KRAB(A) vector was digested by the BglII restriction enzyme. The CCR5TC-KRAB(A) and TdTomato (TdT) sequences were amplified by PCR and integrated into the digested vector by NEBuilder Hifi DNA assembly mix (NEB).

The pDESTTol2CR2-mpeg:mmp14b-HA construct (abbreviated mpeg:mmp14b-HA) was generated in this study. We generated the pME-mmp14b gateway donor construct by integrating the *mmp14b* gene amplified from zebrafish brain complementary DNA into the pME vector. We generated the p3E-4xHA gateway donor construct by integrating the 4xHA sequence amplified from pUB-smHA-KDM5B-MS2 (Addgene, cat. no. 81085) into the p3E vector. The pDESTTol2CR2, p5E-mpeg1.1 (Addgene, cat. no. 75023), pME-mmp14b and p3E-4xHA constructs were recombined by gateway LR Clonase enzyme mix (Thermo Fisher Scientific).

To generate the pDESTTol2CR2-slc1a3b:mmp14b-HA construct (abbreviated slc1a3b:mmp14b-HA), the pDESTTol2CR2, p5E-slc1a3b (Addgene, cat. no. 170205), pME-mmp14b and p3E-4xHA vectors were recombined by gateway LR Clonase enzyme mix.

To generate the rescue human MMP14-eGFP construct, first, silent mutations on MMP14 were introduced that made it resistant to shMMP14_1 (human MMP14_shRNA, Millipore Sigma, cat. no. TRCN0000429314). Wild-type (WT) MMP14 and rescue MMP14 sequences were amplified by PCR and integrated into the BamH1-digested vector pLV-EF1A-BSD (kept in-house) by Gibson Assembly Cloning Kit (NEB).

### Embryo microinjection

The injection mix contained 1% Danieau buffer (diluted from 30% Danieau buffer stock: 58 mM NaCl, 0.7 mM KCl, 0.4 mM MgSO_4_, 0.6 mM Ca(NO_3_)_2_ and 5 mM HEPES, adjusted pH to 7.2), 0.1% phenol red, 5 ng µl^−1^ TPase mRNA and 15 ng µl^−1^ DNA construct in nuclease-free water as the final concentration. We injected 1 nl of the injection mix into one-cell-stage zebrafish embryos with a microinjector. The injected F0 embryos were incubated at 28.5 °C. To generate Tg fish, we injected the pTol2-zcUAS:PSD95.FingR-TdT-CCR5TC-KRAB(A) construct into the *Tg(chata:gal4)* fish. For F0 fish analysis, we injected pDESTTol2CR2-mpeg:mmp14b-HA construct into the *Tg(mpeg:GFP-CAAX)* or *Tg(chat:gal4);Tg(zcUAS:PSD95.FingR-TdT-CCR5TC-KRAB(A))* embryos in WT or *mmp14b*^*−/−*^ background, or pDESTTol2CR2-slc1a3b:mmp14b-HA construct into the *Tg(chat:gal4);Tg(zcUAS:PSD95.FingR-TdT-CCR5TC-KRAB(A))* embryos in WT or *mmp14b*^*−/−*^ background. The embryos were screened by the red heart marker and used for analysis at 14 dpf.

### CRISPR gene knockout

*mmp14b*^*−*/*−*^ fish were kindly provided by B. Black and generated as described previously^53^. To establish the *bcan*^*−*/*−*^ fish, we used the Alt-RTM CRISPR–Cas9 system from IDT. Briefly, the guide RNA (gRNA) complex was prepared by mixing 100 µM tracrRNA and 100 µM crRNA targeting exon 3 (5′-gcgtacggggaagactcacc-3′) or exon 14 (5′-gacctggtaacatggcgcac-3′) of the *bcan* gene and incubated at 95 °C for 5 min. The gRNAs targeting the *bcan* gene were designed by the IDT CRISPR–Cas9 gRNA design tool and CHOPCHOP. The two gRNA complexes were mixed at 1:1 ratio and mixed with 0.5 µg µl^−1^ Cas9 protein diluted in Cas9 working buffer (20 mM HEPES, 150 mM KCl, pH 7.5). The mixture of gRNA complex and Cas9 protein was incubated at 37 °C for 10 min to prepare the RNP complex. Then, 2 nl of the RNP complex was injected into one-cell-stage embryos. Gene deletion was analyzed by PCR (for knockout genome, forward primer: 5′-caaagctttaaacactgctgatg-3′, reverse primer: 5′-tcaggtgtttttgttgttgagtc-3′; for WT genome, forward primer: 5′-tgcagttcaatgggttgtgt-3′, reverse primer: 5′-tcaggtgtttttgttgttgagtc-3′). In the F1 generation, we identified heterozygous mutants by fin genotyping and in-crossed the F1 fish. In F2 generation we identified homozygous mutant and WT fish on this shared genetic background and used age-matched larvae for subsequent experiments.

### Immunohistochemistry on zebrafish fixed brain sections

Zebrafish at 7, 14, 21, 28, 60 and 90 dpf were fixed overnight at 4 °C in 0.1 M phosphate-buffered 4% paraformaldehyde, cryoprotected with 20% sucrose and embedded in optimal cutting temperature (OCT) medium (Sakura Finetek). Immunohistochemistry (IHC) was performed on 18- to 25-μm-thick coronal sections collected on a cryostat and mounted onto VWR Superfrost Plus microscope slides (VWR). Sections were washed in PBS with 0.04% Triton-X (PBST) and incubated with 20% heat-inactivated normal goat serum (NGS; Sigma-Aldrich) in PBST for 1 h. Primary antibodies were diluted in 3% NGS PBST and applied overnight at 4 °C. Sections were then washed with PBST three times and incubated in secondary antibodies diluted in 3% NGS PBST for 1–2 h at room temperature. Sections were washed with PBST three times and mounted with DAPI Fluoromount-G (SouthernBiotech). Stained and mounted sections were imaged on an LSM700 confocal microscope (Zeiss) using ×20 (numerical aperture 0.8) and ×63 (numerical aperture 1.4) objectives. Images were analyzed with ImageJ (v.2.14.0).

For HA staining in the mpeg:mmp14b-HA-injected fish, signal amplification was performed using the TSA Plus Cyanine 3 system (AKOYA Biosciences). After incubation in the primary antibody, sections were incubated in 3% H_2_O_2_ solution for 30 min at room temperature. Sections were washed and secondary antibodies conjugated with HRP were diluted in 3% NGS PBST, applied to sections and incubated for 1–2 h at room temperature. After washing sections, Cy3 dye solution (1:50) was applied to sections and incubated for no more than 8 min at room temperature. Sections were washed and mounted as described above.

Antibodies were: chicken anti-GFP (Aves Labs, cat. no. GFP-1020, 1:1,000), mouse anti-brevican (1:100)^85^, Living Colors DsRed (Clontech, cat. no. 632496, 1:1,000), mouse anti-SV2 (DSHB, 1:500), mouse anti-4C4 (gift from Hitchcock lab, 1:200), rabbit anti-HA (Cell Signaling Technology, cat. no. 3724T, 1:500), rabbit anti-synapsin (Synaptic Systems, cat. no. 106 002, 1:1,000), rat anti-mCherry (16D7) (Thermo Fisher Scientific, cat. no. M11217, 1:1,000), rabbit anti-MBP (1:500, gift from Appel lab^86^). To obtain anti-brevican antibody, SI10-brevican (Addgene, cat. no. 46300) was transfected into HEK293T cells by Lipofectamine 3000 Transfection reagent (Thermo Fisher Scientific) and collected from culture media at 48–72 h and concentrated to 1/10 volume (Amicon Ultra centrifuge filter, Millipore). The *Wisteria floribunda* lectin conjugated with biotin (Sigma-Aldrich, cat. no. L1516, 1:1,000) was mixed with the primary antibody solution. All secondary antibodies were used at 1:500 dilution: goat anti-chicken Alexa Fluor (AF) 488, goat anti-mouse AF 488, goat anti-mouse AF 555, goat anti-mouse AF 647, goat anti-rabbit AF 555, goat anti-rat AF 555 (Invitrogen), goat anti-rabbit HRP (Cell Signaling Technology).

To measure the fluorescence intensity, the region was selected by the selection tool and then we measured the mean intensity. Whole hindbrain measurements included an entire coronal section. To measure the intensity from the synaptic region, a 100-µm^2^ region of interest was placed at the dorsal part of the synaptic region and the mean intensity within the region of interest was measured. We measured three different regions from one section and averaged. We averaged values from two or three sections per fish. Means per fish were plotted and used for statistical comparisons. Values were normalized with the average of control for each experiment.

To count the Chat-PSD95^FingR^ puncta, we randomly took images of single dendrites connected with the cell body. We then cropped the dendrite region and set standard thresholding parameters equivalent across all samples to optimize detection of synaptic puncta. After thresholding, we performed the particle analysis and counted the number of the puncta. We analyzed more than one cell per each fish and generated an average value for each fish when two or more were analyzed. Mean values from each fish were plotted and used for statistical comparisons. Key results were verified with fully blinded quantifications.

To measure the total process length of microglia, z-stack images (0.5-µm step size, ×63 objective) encompassing the entire microglia were analyzed using Imaris software (Bitplane, v.9.8.2). The measurement point function was used to calculate the total length of all processes per cell in three-dimensional (3D) view. Total process length of each microglia was calculated and plotted.

### Fluorescence in situ hybridization on zebrafish brain sections

Fluorescence in situ hybridization experiments were performed using the RNAscope technology (Advanced Cell Diagnostics) following the manufacturer’s protocol for fixed-frozen tissue, but without the 60 °C incubation and post-fixation steps before tissue dehydration. Reagents for RNAscope assay were purchased from Advanced Cell Diagnostics. For fish sample preparation, fish were fixed and embedded at 14 dpf and 14-µm-thick sections were collected as described in the IHC section. We used a C1 probe for zebrafish *mmp14b* (Advanced Cell Diagnostics, cat. no. 1061661-C1). IHC following RNAscope was immediately performed after the last wash of the RNAscope protocol. The IHC was performed as described in the IHC section. Stained sections were imaged on an LSM700 confocal microscope using a ×63 objective lens. Images were analyzed with ImageJ.

### Brain injections

Zebrafish larvae at 10–14 dpf and adult fish at 60 dpf were anesthetized with 0.2 mg ml^−1^ tricaine diluted in fish water. For larval injection, larvae were injected with 2 nl of 10 mg ml^−1^ hyaluronidase (Invitrogen) or PBS as a vehicle control into the hindbrain ventricle by using a microinjector. The injection was confirmed by seeing a slight expansion of the ventricle. The injected larvae were transferred to fresh fish water after injection for recovery. For adult injection, adult fish were placed in a mold made from 2% agarose gel. A small hole was made in the skull above the hindbrain using a 31 gauge insulin syringe (BD, cat. no. 328438), a microinjection capillary was inserted from the hole and 40 nl of hyaluronidase or PBS was injected into the hindbrain ventricle. The injected adult fish were transferred to fresh fish water after injection for recovery. We carefully monitored the fish after injection to confirm that they recovered from anesthesia and did not exhibit abnormal swimming behaviors. Only fish that looked healthy were used for further experiments.

### Time-lapse live imaging of synapse dynamics (24–72-h time-lapse)

For the synapse time-lapse assay, *Tg(chata:gal4);Tg(zcUAS:PSD95.FingR-TdT-CCR5TC-KRAB(A))* (*Chat-PSD95*^*FingR*^) fish at 5–14 dpf were used. Larvae were anesthetized with 0.2 mg ml^−1^ tricaine diluted in fish water and mounted in 1.5% low-melting agarose gel on a glass-bottom 35-mm dish (MatTek) and covered with fish water containing 0.2 mg ml^−1^ tricaine. The still z-stack images were acquired on the Nikon or Leica CSU-W1 spinning disk confocal microscope on a custom imaging system controlled by the open-source software Micromanager. We took 40–60-µm z-stack images with 0.5-µm step size. After taking the first image, larvae were carefully removed from agarose gel, placed in a Petri dish with fresh fish water and kept in the fish facility at 28.5 °C with feeding overnight. At the second time point, the larvae were re-mounted and imaged as previously described. For the longer time courses the same procedure was repeated for up to 72 h.

The images were processed by ImageJ software as follows: for analysis of average synapse turnover over two time points, two images of the same dendrite were obtained. We defined Chat-PSD95^FingR^ puncta observed only on the first image as ‘lost synapses’, puncta observed only on the second image as ‘new synapses’ and puncta visible in both images as ‘stable synapses’. We carefully compared dendrite shapes and synapses between the first and second images and manually counted the numbers of the new, lost and stable synapses from the same region of each dendrite, analyzing regions only where Chat-PSD95^FingR^ puncta were clearly defined, and normalized per length of dendrites (averaged from values across two time points) analyzed. At least one cell per fish was analyzed and average values for each fish were used for graphing and statistical analyses. For merged representative images, we performed image registration by using the bUnwarpJ tool in ImageJ. For the representative reconstructed synapse images, we used Imaris software (Bitplane, v.9.8.2). We performed 3D reconstructions using the surfacing function on Imaris.

To quantify synapse size, we measured the maximum diameter of stable and new synapses at *t* = 6 using the line function in ImageJ. New synapse diameters were normalized to the average of stable synapse diameters for each cell. To quantify the synapse distance to the nearest synapse, we measured the dendrite length from nearest synapses (any synapses including stable and new) by using the segmented line function at *t* = 6. The values were normalized by the average of stable synapses for each cell. The values from each synapse were plotted and used for statistical analysis.

### Imaging of microglia–synapse contact

*Tg(mpeg:GFP-CAAX);Tg(chat:gal4);Tg(zcUAS:PSD95.FingR-TdT-CCR5TC-KRAB(A))* at 10–12 dpf were mounted for live imaging. For the hyaluronidase injection experiment, we injected hyaluronidase or PBS as described in the brain injection section and started sample preparation at 4 h post injection. Time-lapse imaging was performed on a Nikon CSU-W1 spinning disk/high speed widefield confocal microscope. We took 40–60-µm z-stack images (step size: 0.5 µm) with 5-min intervals for 30 min to 1 h. The images were processed by ImageJ software. To count the microglia–synapse contacts, we carefully analyzed single z-stack images and counted the number of Chat-PSD95^FingR^ puncta colocalized with microglial processes. TdT puncta completely merged with GFP signals from microglial processes were defined as ‘contacted synapses’. To analyze total process movement of microglia, we adapted a previously published method^87^. Briefly, a single process was chosen per microglia and process length was measured at each time point to calculate the total amount of process tip movement over the entire imaging session to obtain the sum of the total process motility.

### Microglia ablation

We bred fish harboring *Tg(mpeg:gal4);Tg(UAS:NTR-mCherry)* to express nitroreductase (NTR) under control of the *mpeg* promoter. Fresh 5 mM metronidazole (Mtz; Sigma-Aldrich) was prepared in fish water containing 0.2% DMSO before every experiment. Mtz powder was completely dissolved by vigorous shaking. Larvae at 13 dpf were treated with 5 mM Mtz in a Petri dish for 24 h in the dark and fixed at 14 dpf. Control larvae were treated with embryo medium with 0.2% DMSO. For nontransgenic control, we used nontransgenic siblings with no mCherry fluorescence in myeloid cells.

### Light startle behavior assay

Zebrafish larvae at 14 dpf were transferred into a 96-well plate (Cytiva, cat. no. 7701-1651) and placed in the DanioVision chamber (Noldus). Fish were habituated in the dark for 30 min before the behavior paradigm started. After habituation, fish movement was tracked according to the following setup: 10-min recording before stimuli; 2-s white light stimulus and 2-s dark repeated 10 times; 10-min recording after stimuli. We repeated the same trial five times with no inter-trial intervals. At the conclusion of the experiment all wells were checked for fish viability (normal swimming). Any wells with dead fish were excluded from the analysis.

Startle behavior was analyzed using EthoVision software. To analyze baseline locomotor activity, total distance moved and maximum velocity during the first 10 min of recording before the stimuli in Trial 1 were calculated from each fish and plotted. To analyze light startle behavior, a plot of velocity over time was binned into 2-s time intervals beginning at 10 s before the first stimulus and ending at the end of stimulus sessions. The maximum velocity within each bin was plotted over time. The ‘maximum response’ was defined as the highest velocity achieved over the entire stimulation period.

### Forced swim paradigm

As per previous studies^63,88^, zebrafish larvae (*n* = 5–15 fish per dish) at 13 dpf were placed in 60-mm Petri dishes with or without a stirrer bar (Fisherbrand, cat. no. 14-513-65) on a stir plate set at 650 rpm for 8 h per day. The experiment was performed for 2 consecutive days. To perform the forced swim paradigm in the microglia-ablated fish, *Tg(mpeg:gal4);Tg(UAS:NTR-mCherry)* fish or nontransgenic siblings swam in 5 mM Mtz in fish water with or without a stirrer bar. Larvae were immediately fixed for IHC after the end of the forced swim paradigm at 14 dpf.

For synapse turnover assays, images before forced swim were taken in the morning of day 1 and then fish were placed in a Petri dish with a stirrer bar for at least 5 h. On day 2, fish were placed in the Petri dish with a stirrer bar for at least 5 h and then images after forced swim were taken. Experiments and analysis were performed as described in the synapse time-lapse assay section above.

### Quantitative PCR with reverse transcription

For RNA extraction from whole zebrafish larvae, we collected 20 larvae in a tube and homogenized them by syringe and needle. RNA extraction was performed using QIAGEN RNeasy Mini Kit following the manufacturer’s instructions. cDNA synthesis was performed with the High Capacity cDNA Reverse Transcription kit (Applied Biosystems). The quantitative PCR (qPCR) reaction was performed in 10 µl of reaction mixture containing cDNA solution, 5 µM forward and reverse primers, fast SYBR Green Master Mix (Applied Biosystems) and nuclease-free water. qPCR was performed by the QuantStudio 6 Real-Time PCR system (Applied Biosystems). The primers used for qPCR with reverse transcription (RT–qPCR) are *bcan* (forward primer: 5′-gatcgctccgtcagataccc-3′, reverse primer: 5′-gttctccatcgtgccgtagtt-3′) and internal control *ef1a* (forward primer: 5′-ctggaggccagctcaaacat-3′, reverse primer: 5′-atcaagaagagtagtaccgctagcattac-3′). For analysis, we used the ddCt method and showed relative expression levels to each control sample. We collected 20 larvae from three independent fish pairs and each dot in the graph shows each batch of larvae.

For RNA extraction from human iPS-cell-derived microglia, around 1 million cells were collected per group in a 1.5-ml tube and RNA extraction was performed using QIAGEN RNeasy Plus Mini Kit following the manufacturer’s instructions. cDNA synthesis was then performed with Primescript RT Reagent Kit (Takara Bio) or Superscript IV First Strand Synthesis System (Invitrogen). The qPCR reaction was performed in 10 µl of reaction mixture containing cDNA solution, 1 µM forward and reverse primers, fast SYBR Green Master Mix (Applied Biosystems) and nuclease-free water. qPCR was performed by QuantStudio 6 Real-Time PCR System (Applied Biosystems). The primers used for RT–qPCR are *MMP14* (forward primer: 5′-gcagaagttttacggcttgcaa-3′, reverse primer: 5′-ccttcgaacattggccttgat-3′) and internal control *GAPDH* (forward primer: 5′-tcaacgaccccttcattgac-3′, reverse primer: 5′-atgcagggatgatgttctgg-3′). Relative expression levels were analyzed to each control sample. We collected cells from three independent experiments and each dot in the graph shows the mean value from an independent experiment.

### Human iPS cells induced tri-culture

Human iPS cells (male donor, WTC11 background) were engineered and differentiated, as previously described, with a doxycycline-inducible NGN2 cassette for iNeuron differentiation^89^, doxycycline-inducible SOX9/NFIA cassette for iAstrocyte differentiation^57^ or doxycycline-inducible six transcription factor cassette (6TF: MAFB, IRF5, IRF8, PU.1, CEBPα, CEBPβ) for iTF-Microglia differentiation^58^. Briefly, iPS-cell-derived astrocytes (at 20 d of treatment with ScienCell Astrocyte Medium (ScienCell, cat. no. 1801)) were thawed and seeded at 30,000 cells per cm^2^ on Matrigel (Corning, cat. no. 356231)-coated plates. Seeding of iAstrocytes corresponds to day 0. On day 1, pre-differentiated iNeurons were seeded on top of iAstrocytes at a density of 60,000 cells per cm^2^ and differentiated for 14 d in the following medium: BrainPhys (STEMCELL Technologies, cat. no. 05791) as the base, 0.5 × B27 Supplement Minus Vitamin A (Life Technologies, cat. no. 12587010), 1 × N2 Supplement (Life Technologies, cat. no. 17502-048), 10 ng ml^−1^ NT-3 (PeproTech, cat. no. 450-03), 10 ng ml^−1^ BDNF (PeproTech, cat. no. 450-02), 1 μg ml^−1^ mouse laminin (Thermo Fisher Scientific, cat. no. 23017-015) and 2 μg ml^−1^ doxycycline (Takara Bio, cat. no. 631311). The medium was fully replaced at day 4 with doxycycline and half-replaced without doxycycline every 2–3 d after day 7. At day 7, iPS-cell-6TF was differentiated into microglia in parallel as the following: iPS-cell-6TF was seeded onto poly-d-lysine + Matrigel double-coated plates in the following medium: Essential 8 Basal Medium (Gibco, cat. no. A15169-01) as a base, 10 nM ROCK inhibitor (Fisher Scientific, cat. no. 125410) and 2 μg ml^−1^ doxycycline. Then, 2 d later at day 9, medium was replaced with the microglia differentiation medium as the following: Advanced DMEM/F12 Medium (Gibco, cat. no. 12634-010) as a base, 1 × Antibiotic-Antimycotic (Gibco, cat. no. 15240-062), 1 × GlutaMAX (Gibco, cat. no. 35050-061), 2 μg ml^−1^ doxycycline, 100 ng ml^−1^ Human IL-34 (Peprotech, cat. no. 200-34) and 10 ng ml^−1^ Human GM-CSF (Peprotech, cat. no. 300-03). Another 2 d later at day 11, medium was replaced to the microglia differentiation medium with addition of 50 ng ml^−1^ Human M-CSF (Peprotech, cat. no. 300-25) and 50 ng ml^−1^ Human TGFB1 (Peprotech, cat. no. 100-21C). At day 15, microglia was either fixed for mono-culture immunofluorescence or dissociated for adding into tri-culture. TrypLE (Gibco, cat. no. 12605010) was used to dissociate the microglia from the plate. Then dissociated microglia were added at 30,000 cells per cm^2^ to the astrocyte and neuron co-culture to form the tri-culture system. At day 16, the tri-culture system was then used in western blot and immunofluorescence as described later.

### Process length analysis through immunofluorescence

Cells were washed carefully with PBS once and then fixed with 4% paraformaldehyde for 30 min at room temperature. After washing with PBS three times, cells were permeabilized with SuperBlock buffer (Thermo Scientific, cat. no. 37515) containing 0.1% Triton X-100 for 1 h at room temperature. Cells were then incubated with primary antibodies diluted in PBS at 4 °C overnight, then washed and incubated with secondary antibodies diluted in PBS for 2 h at room temperature, then washed, mounted and imaged on a Leica Widefield microscope. Antibodies were: chicken anti-MAP2 (1:500, Invitrogen, cat. no. PA116751), rabbit anti-IBA1 (1:200, Wako Chemicals, cat. no. 019-19741), mouse anti-S100β (1:200, Sigma-Aldrich, cat. no. S2532), guinea pig anti-vGLUT2 (1:200, Synaptic Systems, cat. no. 135404).

### Protein detection by western blotting

For brevican detection, cell culture medium was collected in 1.5-ml Ep tubes separately and centrifuged at 16,200*g* for 1 min at 4 °C to get rid of potential debris. Then the supernatant was concentrated tenfold (Amicon Centrifuge filter unit, 10kD, EMD Millipore). Next, 4 × Laemmli Sample Buffer (Bio-Rad) was added to concentrated supernatant and boiled at 100 °C for 10 min. At the same time, cells were collected in 1 × Laemmli Sample Buffer separately and boiled at 100 °C for 10 min. For western blotting, 20 μg of total proteins were loaded into 4–15% Mini-PROTEAN TGX Precast Protein Gels (Bio-Rad). Subsequently, the gels were transferred into Immun-Blot PVDF Membrane (Bio-Rad). The membranes were blocked by 5% milk (Bio-Rad, cat. no. 1706404), followed by incubation with primary antibodies at 4 °C overnight. Antibodies were: rabbit anti-Brevican (1:1,000, Thermo Fisher Scientific, cat. no. PA552477) and mouse anti-MAP2 (1:1,000, Thermo Scientific, cat. no. 13-1500) as inner control. The membranes were washed three times with TBST (Tris-buffered saline with 0.1% Tween detergent, Bio-Rad) and then incubated with secondary antibodies for 2 h at room temperature. The membranes were then washed three times with TBST and developed in Clarity Western ECL Substrate (Bio-Rad, cat. no. 1705060). Images were taken using ChemiDoc Imaging Systems and analyzed using ImageJ.

For MMP14 detection, microglial cells were collected in 1 × Laemmli Sample Buffer and boiled at 100 °C for 10 min. The same procedure outlined above was followed, except that the primary antibodies used were rabbit anti-MMP14 (1:1,000, Cell Signaling Technology, cat. no. 26424S) and rabbit anti-β-Actin (1:1,000, Cell Signaling Technology, cat. no. 4970S) as inner control.

### Gene expression analysis of human fetal microglia and iPS-cell-derived microglia

Bulk RNA sequencing data of human fetal microglia and iPS-cell-derived microglia were obtained from the previous study^55^. Within each group, genes are ranked by their mean transcript per kilobase million across samples. Scatter plots showing the top 8,000 of gene expression rank were generated using matplotlib and Python.

### Proteomics analysis

#### Sample preparation for proteomics

For proteomics analysis of the human iPS-cell-derived tri-culture system, protein samples were denatured with 50 µl of 6 M guanidine HCl in 100 mM Tris pH 8. Proteins were then reduced and alkylated by addition of Tris(2-carboxyethyl)phosphine (TCEP) and 2-chloroacetamide to a final concentration of 10 mM and 40 mM, respectively, before incubation at 95 °C and 700 rpm for 7 min. Guanidine was diluted 1:5 by the addition of 200 µl of 100 mM Tris pH 8. Proteins were digested by adding 1 µg of trypsin to each sample and incubated overnight (37 °C, with shaking at 800 rpm).

Digested peptides were acidified with 50 µl of 10% trifluoroacetic acid (TFA) before desalting using NEST UltraMicrospin tips attached to a vacuum manifold. Each tip was pre-activated with 100 µl of 80% acetonitrile (ACN), 0.1% TFA then washed three times with 0.1% TFA (wash buffer). Peptides were loaded on the tips, before washing four times with wash buffer. Peptides were eluted by centrifugation after addition of 150 µl of 50% ACN, 0.25% formic acid (FA), dried in a SpeedVac and resuspended in 60 µl of 0.1% FA. Resuspended peptide samples were filtered by centrifugation (Millipore 0.45-µm hydrophilic low protein binding Durapore Membrane) and diluted 1:10 before 3 µl was injected onto the liquid chromatography–mass spectrometry system.

For analysis of the zebrafish brain extracellular proteome, sulfo-NHS-biotin solution was injected into the brain ventricle. Briefly, zebrafish at 14, 28 and 60 dpf were injected with 2, 10 and 40 nl of 20 mg ml^−1^ sulfo-NHS-biotin (Thermo Scientific, cat. no. A39256) in PBS, respectively. Representative images derived from injected fish were fixed at 1 h post injection from sections stained with Streptoavidin conjugated with AF 647 (Invitrogen) for 2 h after the blocking procedure.

To prepare protein lysates, brains were dissected at 1 h post injection and incubated in the quenching solution (50 mM glycine in PBS). We pooled ten brains for 14 dpf and three brains for 28 dpf for one replicate. Brains were homogenized in 200 µl of 1%-SDS RIPA buffer with pipetting and incubated on ice for 30 min. The homogenized brains were sonicated, incubated at 95 °C for 5 min and incubated at 4 °C with rotation after adding SDS-free RIPA buffer. The samples were ultracentrifuged at 100,000*g* for 45 min at 4 °C. The supernatants were quantitated using BCA assay (Thermo Scientific). Equivalent protein amounts were incubated with Streptavidin magnetic beads (New England Biolabs, cat. no. S1420S) overnight at 4 °C. Beads were resuspended in 18 µl of 20 mM Tris-HCl (pH 8.0), then 0.8 µl of 100 mM dithiothreitol was added for 30 min at room temperature, followed by 1.2 µl of 100 mM iodoacetamide and incubation in the dark for 10 min. Next, 500 ng of trypsin (Promega, cat. no. V5113) was added and digested overnight at 37 °C, then an additional 500 ng of trypsin added for 4 h at 37 °C. Digestion was quenched with 2% FA, and peptides were desalted using C18 ZipTip columns (Millipore, cat. no. ZTC18S096).

#### Liquid chromatography–mass spectrometry

For proteomics analysis on the human iPS-cell-derived tri-culture system, samples were analyzed on an Orbitrap Exploris 480 mass spectrometry system (Thermo Fisher Scientific) equipped with a Neo Vanquish ultra-high-pressure liquid chromatography system (Thermo Fisher Scientific) interfaced via a Nanospray Flex source. Separation was performed using a 15-cm-long PepSep column with a 150-µm inner diameter packed with 1.5-µm Reprosil C18 particles. Mobile phase A consisted of 0.1% FA, and mobile phase B consisted of 0.1% FA, 80% ACN. Peptide samples were separated by a gradient of 3% to 28% mobile phase B over 67 min followed by an increase to 40% B over 5 min. Chromatographic gradients ended with a wash at 95% B for 8 min. The flow rate was 600 nl min^−1^ throughout, outside of sample loading, which was pressure controlled, with a maximum pressure of 300 bar. Spectra were acquired in a data-independent manner (DIA) with a full scan across 350–1,050 *m*/*z* in the Orbitrap at 60,000 resolving power (at 200 *m*/*z*) with a normalized auto gain control target of 300%, a radio frequency lens setting of 40% and a maximum ion injection time of ‘Auto’. DIA isolation windows were generated automatically, covering 350–1,050 *m*/*z* space with 20-*m*/*z-*wide windows and 0.5-*m*/*z* overlap. Peptide ions were fragmented using a normalized higher-energy collisional dissociation collision energy of 28%, with an auto gain control target of ‘standard’, a maximum injection time of 40 ms, with scan range mode set to ‘auto’, and a resolving power of 15,000. Gas phase fraction data were collected from a pooled sample as described in ref. ^90^ to supplement our existing spectral libraries. Resolution, fragmentation and chromatographic parameters were identical to DIA runs, with full scans covering 110-*m*/*z* windows with 5-*m*/*z* overlap (for example, 345–455, 445–555 and so on) and fragment scan windows of 4 *m*/*z*.

For the zebrafish brain extracellular proteome, the experiment was performed using a NanoAcquity UPLC system (Waters) connected to an Orbitrap Fusion Lumos Mass Spectrometer (Thermo Scientific). A binary solvent system was used, consisting of 0.1% FA in water (solvent A) and 0.1% FA in ACN (solvent B). Chromatographic separation was performed using an Easy-Spray HPLC column (75 μm × 150 mm, Thermo Scientific) at a flow rate of 300 nl min^−1^. A linear gradient elution was applied, increasing solvent B from 5% to 30% over 72 min. For data-dependent tandem mass spectrometry acquisition, precursor ions were measured in the Orbitrap over an *m*/*z* range of 375–1500 at a resolution of 120,000 full-width at half-maximum (FWHM) (cycle time: 3 s; maximum injection time: 50 ms; intensity threshold: 2 × 10^4^). Fragment ions generated by higher-energy collisional dissociation were detected in the Orbitrap with a resolution of 30,000 FWHM (collision energy: 30%; quadrupole isolation window: 1.6 *m*/*z*; maximum injection time: 100 ms).

#### Mass spectrometry data searching and analysis

For the human iPS-cell-derived tri-culture system, DIA data files were searched in Spectronaut (v.19.5) against a spectral library developed in-house from the full, reviewed human proteome with isoforms (downloaded from UniprotKB, 30 May 2023) and supplemented with our gas phase fraction acquisition files. The search was performed with default settings, except for cross run normalization, which was not used. Results were output in the MSStats format. Peptide features were summarized into protein abundances and the two groups were compared using the dataProcess and groupComparison functions of the MSstats^91^ package (v.4.12.0), respectively. The group comparison results were used to generate the significance and fold change for proteins depicted in Fig. 4. For the proteomics results, see Supplementary Tables 3 and 4.

For the zebrafish brain extracellular proteome, acquired raw mass spectrometry data were converted to peak lists using in-house PAVA software, followed by analysis using Protein Prospector (v.6.6.5)^92^. Data were searched against a concatenated database of *D. rerio* entries from the UniprotKB database (downloaded April 2025: 26,728 protein entries) and sequence-randomized decoy entries, employing precursor and fragment mass tolerances of ±10 ppm and ±20 ppm, respectively. Carbamidomethylation of cysteine residues was set as a constant modification, while variable modifications included N-terminal acetylation, N-terminal methionine acetylation and oxidation, pyroglutamate formation from N-terminal glutamine, loss of the protein N-terminal methionine, N-terminal methionine excision followed by acetylation of the new N terminus, oxidation of methionine and oxidation of proline (proline replaced by hydroxyproline), allowing up to three modifications per peptide. Results were thresholded at a false discovery rate of 1% at both the protein and peptide levels based on target:decoy database searching. For label-free quantification of phosphopeptides and proteins, results were output in .blib format, then imported into Skyline for MS1 filtering^93^. The result was filtered by the proteins detected in the zebrafish matrisome project^36^.

### Computational analysis

We fitted models for the synapse population dynamics to the synapse lifetime imaging data, to determine whether the observations are better described by a single or two (dynamic and stable) internal states of the synapses as well as to estimate the four key model parameters (synapse birth rate, decay rate of dynamic synapses, transition rate from dynamic to stable synapses and the decay rate of stable synapses) for *mmp14*^*+/+*^ and *mmp14b*^*−/−*^ conditions, as shown in Fig. 7. See Supplementary Data 1 for mathematical derivation of the models and their predictions of experimental observations and numerical fitting.

### Statistical analysis

GraphPad Prism v.10.3.0 was used for all statistical analyses. Statistical tests used, number of *n* replicates and *P* values are described in figures, text and figure legends. No statistical methods were used to pre-determine sample sizes but our sample sizes are similar to those reported in previous publications^35^. All statistical analyses unless otherwise noted were performed on means of multiple technical replicates per animal. For comparisons of two groups, we used a two-sided *t*-test with Welch’s correction to correct unequal variance. Data were usually normally distributed. Datasets with one than two groups were analyzed with one-way or two-way analysis of variance (ANOVA).

### Reporting summary

Further information on research design is available in the Nature Portfolio Reporting Summary linked to this article.

## Online content

Any methods, additional references, Nature Portfolio reporting summaries, source data, extended data, supplementary information, acknowledgements, peer review information; details of author contributions and competing interests; and statements of data and code availability are available at 10.1038/s41593-025-02153-4.

## Supplementary information

Reporting Summary

Supplementary Video 1 Representative time lapse image of microglia contact with *Chat-PSD95*^*FingR*^ puncta shown in Extended Data Fig. 5c. Z-stack images taken every 5 min for 1 h. White arrowheads indicate a synapse before and after microglial contact and yellow arrowheads indicate a synapse contacted by microglia.

Supplementary Table 1 Analyzed proteomics result from zebrafish brain in Extended Data Fig. 1e. Comparison between 14 dpf and 28 dpf.

Supplementary Table 2 Analyzed proteomics result from zebrafish brain in Extended Data Fig. 1e. Comparison between 14 dpf and 60 dpf.

Supplementary Table 3 Analyzed proteomics result from iPSC-derived tri-culture system. Analyzed result used for Fig. 4e.

Supplementary Table 4 Analyzed proteomics result from iPSC-derived tri-culture system. Analyzed expression for each protein in Fig. 4. Results from four independent samples are included.

Supplementary Data 1 Derivation and fitting of the computational model.

## Source data

Source Data Fig. 1 Statistics source data for Fig. 1.

Source Data Fig. 2 Statistics source data for Fig. 2.

Source Data Fig. 3 Statistics source data for Fig. 3.

Source Data Fig. 4 Uncropped western blot of Fig. 4c.

Source Data Fig. 4 Statistics source data for Fig. 4.

Source Data Fig. 5 Statistics source data for Fig. 5.

Source Data Fig. 6 Statistics source data for Fig. 6.

Source Data Extended Data Fig. 2 Statistics source data for Extended Data Fig. 2.

Source Data Extended Data Fig. 3 Statistics source data for Extended Data Fig. 3.

Source Data Extended Data Fig. 4 Statistics source data for Extended Data Fig. 4.

Source Data Extended Data Fig. 5 Statistics source data for Extended Data Fig. 5.

Source Data Extended Data Fig. 7 Statistics source data for Extended Data Fig. 7.

Source Data Extended Data Fig. 8 Uncropped western blot of Extended Data Fig. 8c,g.

Source Data Extended Data Fig. 8 Statistics source data for Extended Data Fig. 8.

Source Data Extended Data Fig. 9 Statistics source data for Extended Data Fig. 9.

## Data Availability

All data needed to evaluate the conclusions in the paper are present in the paper, Supplementary Tables 1–4, Supplementary Data 1 and Supplementary Video 1. All additional information will be made available upon request to the authors. The mass spectrometry proteomics data have been deposited to the ProteomeXchange Consortium via the PRIDE partner repository. The dataset identifier for the proteomics result from zebrafish brain is PXD069328, and for the proteomics result from iPS-cell-derived tri-culture system is PXD060477. Source data are provided with this paper.

## Code availability

The open-source code established by computational modeling is available via GitHub at https://github.com/ChristophKirst/developmental_synapse_remodeling, together with the scripts and datasets used to generate the figures, as well as documentation on how to run the code.
