# Supplementary material for: Extracellular matrix proteolysis maintains synapse plasticity during brain development
Source: Nat Neurosci. 2025 Dec 22;29(3):567–80. doi: 10.1038/s41593-025-02153-4 (PMC12971489; doi:10.1038/s41593-025-02153-4)
Supplement: Supplementary file 1 — Reporting Summary [file 41593_2025_2153_MOESM1_ESM.pdf]

nature portfolio

Corresponding author(s): Anna V. Molofsky

Last updated by author(s): Oct 13, 2025

Reporting Summary

Nature Portfolio wishes to improve the reproducibility of the work that we publish. This form provides structure for consistency and transparency in reporting. For further information on Nature Portfolio policies, see our Editorial Policies and the Editorial Policy Checklist.

Statistics

For all statistical analyses, confirm that the following items are present in the figure legend, table legend, main text, or Methods section.

n/a	Confirmed	
☐	☒	The exact sample size (*n*) for each experimental group/condition, given as a discrete number and unit of measurement
☐	☒	A statement on whether measurements were taken from distinct samples or whether the same sample was measured repeatedly
☐	☒	The statistical test(s) used AND whether they are one- or two-sided<br>*Only common tests should be described solely by name; describe more complex techniques in the Methods section.*
☒	☐	A description of all covariates tested
☐	☒	A description of any assumptions or corrections, such as tests of normality and adjustment for multiple comparisons
☐	☒	A full description of the statistical parameters including central tendency (e.g. means) or other basic estimates (e.g. regression coefficient) AND variation (e.g. standard deviation) or associated estimates of uncertainty (e.g. confidence intervals)
☐	☒	For null hypothesis testing, the test statistic (e.g. *F*, *t*, *r*) with confidence intervals, effect sizes, degrees of freedom and *P* value noted<br>*Give P values as exact values whenever suitable.*
☒	☐	For Bayesian analysis, information on the choice of priors and Markov chain Monte Carlo settings
☒	☐	For hierarchical and complex designs, identification of the appropriate level for tests and full reporting of outcomes
☒	☐	Estimates of effect sizes (e.g. Cohen's *d*, Pearson's *r*), indicating how they were calculated

Our web collection on statistics for biologists contains articles on many of the points above.

Software and code

Policy information about availability of computer code

Data collection	Confocal images were acquired using Zeiss Zen 2.6 software on an LSM700 Microscope. Live imaging images were acquired using Micro-Manager software 2.0 on Nikon or Leica CSU-W1 spinning disk microscope. Fish behavior was collected and analyzed by using Noldus EthoVision XT software (version 16.0.1536) on DanioVision system. RT-qPCR results were acquired using Applied Biosystems QuantStudio Real-Time PCR Sftware (version 1.7.2) on QuantStidio 6 Real-Time PCR system. Western blotting images were acquired using Bio-Rad Image Lab software on Chemidoc Imaging system.
Data analysis	Images were processed and analyzed using ImageJ/Fiji (NIH, version 2.14.0) and Imaris software (Bitplane, version 9.8.2). Fish behavior analysis was performed using EthoVision XT software (version 16.0.1536). RT-qPCR analysis was performed using QuantStudio Real-Time PCR Sftware (version 1.7.2). Proteomics data searching and analysis were performed using Spectronaut (version 19.5) and MSstats package (version 4.12.0). Computational modeling were performed using python version 3.11 and open source modules (numpy version 2.2 3, scipy version 1.15.2).

For manuscripts utilizing custom algorithms or software that are central to the research but not yet described in published literature, software must be made available to editors and reviewers. We strongly encourage code deposition in a community repository (e.g. GitHub). See the Nature Portfolio guidelines for submitting code & software for further information.

Data

Policy information about availability of data

All manuscripts must include a data availability statement. This statement should provide the following information, where applicable:

- Accession codes, unique identifiers, or web links for publicly available datasets
- A description of any restrictions on data availability
- For clinical datasets or third party data, please ensure that the statement adheres to our policy

Data Availability:
The mass spectrometry proteomics data have been deposited to the ProteomeXchange Consortium via the PRIDE partner repository. The dataset identifier is PXD060477 for the dataset from iPSC-derived tri-culture system, and PXD069328 for the dataset from zebrafish brain.
Additional information will be made available upon request to the authors.

Code Availability:
The code established by computational modeling is available as an open-source github repository at “https://github.com/ChristophKirst/developmental_synapse_remodeling”, together with the scripts and data sets used to generate the figures, as well as documentation how to run the code.

Research involving human participants, their data, or biological material

Policy information about studies with human participants or human data. See also policy information about sex, gender (identity/presentation), and sexual orientation and race, ethnicity and racism.

Reporting on sex and gender	*Use the terms sex (biological attribute) and gender (shaped by social and cultural circumstances) carefully in order to avoid confusing both terms. Indicate if findings apply to only one sex or gender; describe whether sex and gender were considered in study design; whether sex and/or gender was determined based on self-reporting or assigned and methods used. Provide in the source data disaggregated sex and gender data, where this information has been collected, and if consent has been obtained for sharing of individual-level data; provide overall numbers in this Reporting Summary. Please state if this information has not been collected. Report sex- and gender-based analyses where performed, justify reasons for lack of sex- and gender-based analysis.*
Reporting on race, ethnicity, or other socially relevant groupings	*Please specify the socially constructed or socially relevant categorization variable(s) used in your manuscript and explain why they were used. Please note that such variables should not be used as proxies for other socially constructed/relevant variables (for example, race or ethnicity should not be used as a proxy for socioeconomic status). Provide clear definitions of the relevant terms used, how they were provided (by the participants/respondents, the researchers, or third parties), and the method(s) used to classify people into the different categories (e.g. self-report, census or administrative data, social media data, etc.) Please provide details about how you controlled for confounding variables in your analyses.*
Population characteristics	*Describe the covariate-relevant population characteristics of the human research participants (e.g. age, genotypic information, past and current diagnosis and treatment categories). If you filled out the behavioural & social sciences study design questions and have nothing to add here, write "See above."*
Recruitment	*Describe how participants were recruited. Outline any potential self-selection bias or other biases that may be present and how these are likely to impact results.*
Ethics oversight	*Identify the organization(s) that approved the study protocol.*

Note that full information on the approval of the study protocol must also be provided in the manuscript.

Field-specific reporting

Please select the one below that is the best fit for your research. If you are not sure, read the appropriate sections before making your selection.

☒ Life sciences ☐ Behavioural & social sciences ☐ Ecological, evolutionary & environmental sciences

For a reference copy of the document with all sections, see nature.com/documents/nr-reporting-summary-flat.pdf

Life sciences study design

All studies must disclose on these points even when the disclosure is negative.

Sample size	No statistical methods were used to predetermine sample size. Samples size were determined according to standards in the field.
Data exclusions	For behavioral assay, some data were excluded because these animals died during behavioral paradigm.
Replication	For the data quantified, we performed at least 3 independent experiments. For the result not quantified, we performed at least 2 independent experiments. All attempts at replication were successful. For RNAscope result in Extended Data Fig. 6, we only performed single experiment but similar result were observed at least n=3 fish.

Randomization	All animals were selected randomly. When animals were separated into several groups, animals were randomly assigned to each condition.
Blinding	Key results in the paper were analyzed in fully blinded way.

Reporting for specific materials, systems and methods

We require information from authors about some types of materials, experimental systems and methods used in many studies. Here, indicate whether each material, system or method listed is relevant to your study. If you are not sure if a list item applies to your research, read the appropriate section before selecting a response.

Materials & experimental systems

n/a	Involved in the study
☐	☒ Antibodies
☐	☒ Eukaryotic cell lines
☒	☐ Palaeontology and archaeology
☐	☒ Animals and other organisms
☒	☐ Clinical data
☒	☐ Dual use research of concern
☒	☐ Plants

Methods

n/a	Involved in the study
☒	☐ ChIP-seq
☒	☐ Flow cytometry
☒	☐ MRI-based neuroimaging

Antibodies

Antibodies used

For zebrafish sections,
anti-GFP chicken polyclonal antibody (Aves Labs GFP-1020, AB_2307313),
anti-brevican mouse monoclonal antibody (Staudt et al., 2015, reference #92),
Living Colors DsRed polyclonal antibody (Clontech 632496, AB_10013483),
anti-SV2 mouse monoclonal antibody (DSHB, AB_2315387),
anti-4C4 mouse monoclonal antibody (Gift from Hitchcock lab, Sigma 92092321, AB_10013752),
anti-HA rabbit monoclonal antibody (Cell Signaling Technology 3724T, AB_1549585),
anti-synapsin rabbit polyclonal antibody (Synaptic Systems 106002, AB_887804),
anti-mCherry rat monoclonal antibody (Thermo Fisher Scientific M11217, AB_2536611),
anti-MBP rabbit antibody (Gift from Appel lab, reference #93).

For human iPSC-derived cultures,
anti-Brevican rabbit polyclonal antibody (Thermo Fisher Scientific, PA552477, AB_2638590),
and anti-MAP2 mouse monoclonal antibody (Thermo Scientific, 13-1500, AB_2533001),
anti-MMP14 rabbit monoclonal antibody (Cell Signaling Technology, 26424S),
anti-β-Actin rabbit monoclonal antibody (Cell Signaling Technology, 4970S, AB_2223172),
anti-MAP2 chicken polyclonal antibody (Invitrogen, PA116751, AB_2138189),
anti-IBA1 rabbit polyclonal antibody (Wako Chemicals 019-19741, AB_839504),
anti-S100β mouse monoclonal antibody (Sigma-Aldrich, S2532, AB_477499),
anti-vGLUT2 guinea pig monoclonal antibody (Synaptic Systems, 135404, AB_887884).

Validation

For antibodies used for zebrafish experiments, antibodies were commonly used and validated in previous fish studies. For anti-brevican antibody, we validated the specificity by our brevican knock-out fish (Extended Data Fig. 3f-g).
For antibodies used for human iPSC-derived cultures, the antibodies were commonly used and validated in previous in vitro studies and manufacturer's website. MMP14 antibody was also validated by MMP14 knock-down experiment we performed (Extended Data Fig. 8c). Also, for western blotting data, antibodies were validated by the size of targeted proteins.

Eukaryotic cell lines

Policy information about cell lines and Sex and Gender in Research

Cell line source(s)	The human iPSC (hiPSC) line WTC11 was obtained from the Conklin Lab at Gladstone Institutes/UCSF.
Authentication	hiPSCs were characterized for maintaining capability to differentiate into microglia, astrocytes, and neurons. The WTC11 hiPSC line was also characterized using the StemCell Technologies hPSC Genetic Analysis Kit (cat. no. 07550), which did not reveal any chromosomal abnormalities common in iPSCs that can be detected by qPCR.
Mycoplasma contamination	All WTC11-based lines were regularly tested and confirmed to be negative for mycoplasma using the Universal Mycoplasma Detection Kit (ATCC 30-1012KTM).
Commonly misidentified lines (See ICLAC register)	No commonly misidentified lines were used.

Animals and other research organisms

Policy information about studies involving animals; ARRIVE guidelines recommended for reporting animal research, and Sex and Gender in Research

Laboratory animals	Zebrafish (Danio rerio) up to 90 days post fertilization (dpf) were used in this study (mostly 10-14 dpf). All experiments except behavioral assays were performed by non-pigmented Casper (roy-/-;nacre-/-) background fish. Behavioral assays were performed by pigmented Ekkwill (EKW; ZFIN ID: ZDB-GENO-990520-2) background fish. Already published transgenic fish used in this study were; Tg(mpeg1.1:GFP-CAAX)zh901Tg, Tg(mpeg1.1:gal4)zf2055Tg, Tg(UAS:NTR-mCherry)c264tg, Tg(chata:gal4)mpn202Tg, Tg(ubi:ssncan-GFP)uq25bhTg, Tg(slc1a3b:myrGFP-P2A-H2A-mCherry)vo80Tg, Tg(NBT:dsRed)zf148Tg . Transgenic fish established in this study was; Tg(zcUAS:PSD95.FingR-TdT-CCR5TC-KRAB(A)).
Wild animals	No wild animals were used in this study.
Reporting on sex	Sex was not considered in zebrafish before 28 days post fertilization (28 dpf) because this period is prior to sex determination. We used mixed sex at 60 dpf and 90 dpf.
Field-collected samples	No field-collected samples were used in this study.
Ethics oversight	All animal experiments were approved by the UCSF Institutional Animal Care and Use Committee and Laboratory Animal Resource Center (LARC).

Note that full information on the approval of the study protocol must also be provided in the manuscript.

Plants

Seed stocks	*Report on the source of all seed stocks or other plant material used. If applicable, state the seed stock centre and catalogue number. If plant specimens were collected from the field, describe the collection location, date and sampling procedures.*
Novel plant genotypes	*Describe the methods by which all novel plant genotypes were produced. This includes those generated by transgenic approaches, gene editing, chemical/radiation-based mutagenesis and hybridization. For transgenic lines, describe the transformation method, the number of independent lines analyzed and the generation upon which experiments were performed. For gene-edited lines, describe the editor used, the endogenous sequence targeted for editing, the targeting guide RNA sequence (if applicable) and how the editor was applied.*
Authentication	*Describe any authentication procedures for each seed stock used or novel genotype generated. Describe any experiments used to assess the effect of a mutation and, where applicable, how potential secondary effects (e.g. second site T-DNA insertions, mosiacism, off-target gene editing) were examined.*
