# Supplementary material for: Extracellular matrix proteolysis maintains synapse plasticity during brain development
Source: Nat Neurosci. 2025 Dec 22;29(3):567–80. doi: 10.1038/s41593-025-02153-4 (PMC12971489; doi:10.1038/s41593-025-02153-4)
Supplement: Supplementary file 7 — Derivation and fitting of the computational model. [file 41593_2025_2153_MOESM7_ESM.pdf]

Supplement: Modeling of developmental synapse dynamics

Here we describe the birth-decay processes models used to describe the developmental dynamics of synaptic life times observed in the experiments. We provide derivations of the model predictions, including predictions for the experimental measurements which are used to fit the parameter to the data. In our modeling approach, we assume that synapses are created via a random process and then transition with certain probabilities through one or multiple internal states before they disappear. We show that a model with only a single internal state cannot explain the experimental data. However, already a model with two states of synapses, including a newly generated state as well as a stable state in which the survival rate is higher, describes the experimental data well and fits 21 data points with only 4 parameter. Our analysis indicates that synapses come in two flavors: a dynamic pool into which synapses are born and from which they decays faster, and a stable pool to which new synapses can transition and from which decay is much slower. By fitting these two states models to control and knock-out data we find that key parameters change in accordance with the main interpretation of our data as described in the main manuscript.

In the following we will introduce the birth-decay processes model for developmental synaptic dynamics, derive key predictions of the models, including predictions for the experimental measurements, and describe our fitting procedures to the experimental data. As the one state model can be viewed as a special case of the two state model we consider the two state model first and specialize to the one state model in the end. If not stated otherwise we model the number or density of synapses on a fixed segment of a dendrite.

Open-source python code to simulate the model and data fitting routines are available online [1] or can be obtained upon reasonable request.

1 Two state synapse model

We use the following conventions in describing our model. Parameters are denoted using Greek letters, while variables use Latin characters. A random variable X is denoted with a capital letter while the corresponding lower case x will denote an instance of that random variable and $\bar{x}$ its average. We denote probability measures by $\mathbb{P}$ and expectations by $\mathbb{E}$.

Our modeling approach is based on birth-decay stochastic process models that have been previously used to describe nuclear decay or dynamics of epidemics [2, 3]. We assume two types of synapses:

- "new" synapses denoted by n of recently generated and
- "stable" synapses s that transitioned to a more stable state.

The number of new and stable synapses are random variables and denoted by $N(t)$ and $S(t)$ and these two numbers fully characterize the state of the system. New synapses are generated via a homogeneous Poisson process with a birth rate $\rho_{\emptyset,n}$. A single new synapse will transition to stable synapses with a rate $\gamma_{n,s}$ or decay with a rate $\gamma_{n,d}$. Given $N(t)$ new synapses at time t, the total transition rates are thus

$$\rho_{n,s}(t) = \gamma_{n,s}N(t) \qquad \rho_{n,d}(t) = \gamma_{n,d}N(t)$$

The total loss of new synapses is

$$\rho_{n,-}(t) = \rho_{n,d}(t) + \rho_{n,s}(t) = \gamma_{n,-}N(t)$$

were we introduced the total loss rate of new synapses

$$\gamma_{n,-} = \gamma_{n,s} + \gamma_{n,d}$$

Each stable synapses will decay with a typically smaller rate $\gamma_{s,d}$. Given $S(t)$ stable synapses the decay rate is

$$\rho_{s,d}(t) = \gamma_{s,d}S(t)$$

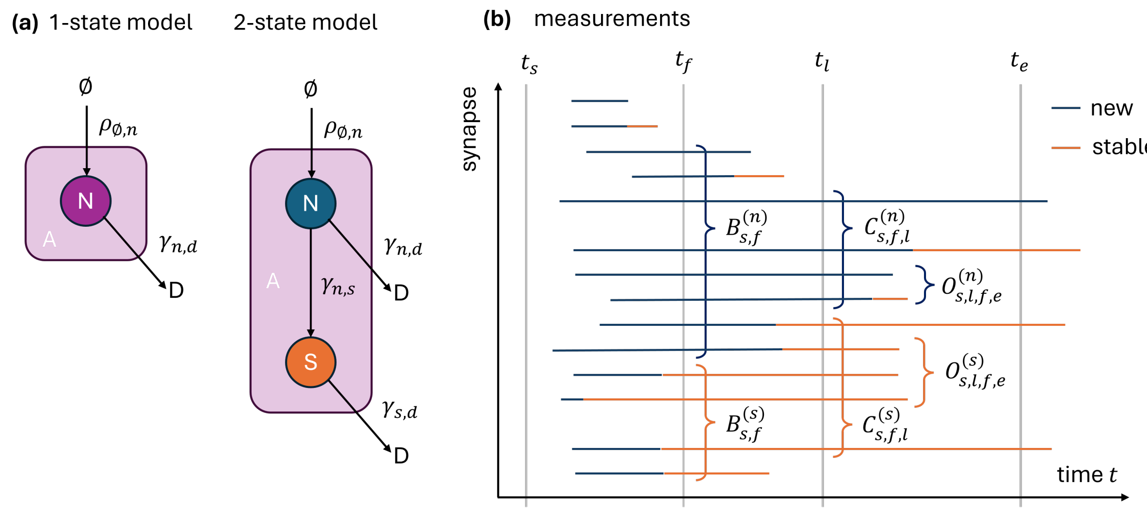

Figure 1: Model schematic. **(a)** 1- and 2-state model together with model parameter. **(b)** Measures of synapse counts given four discrete observation times $t_s < t_f < t_l < t_e$ (start, first, last, end) and possible scenarios of synapse dynamics contributing to these counts. $B^{(n)}_{s,f}$ and $B^{(s)}_{s,f}$ are the measured number of new and stable synapses *becoming visible* between t_s and t_f, , i.e they got generated after t_s and are still visible at t_f. $C^{(n)}_{s,f}$ and $C^{(s)}_{s,f}$ are the measured number of new and stable synapses becoming visible between t_s and t_f and *continuing to be visible until* t_l. Finally, $O^{(n)}_{s,f,l,e}$ and $O^{(s)}_{s,f,l,e}$ are the *occurred* synapses that become visible between t_s and t_f and decay between t_l and t_e. The only experimentally observable numbers are the total counts $B_{s,f} = B^{(n)}_{s,f} + B^{(s)}_{s,f}$ (eq. (6)) $C_{s,f,l} = C^{(n)}_{s,f,l} + C^{(s)}_{s,f,l}$ (eq. (7)) and $O_{s,f,l,e} = O^{(n)}_{s,f,l,e} + O^{(s)}_{s,f,l,e}$ (eq. (8)) for a limited discrete set of measurement times.

The probability to be in a specific state (n, s) at time t is denoted as

$$p(n, s, t) = \mathbb{P}(N(t) = n, S(t) = s)$$

The model is fully specified by its four parameters

$$\theta = \left(\rho_{\emptyset,n}, \gamma_{n,d}, \gamma_{n,s}, \gamma_{s,d}\right)$$

as well as the distribution of initial conditions $p(n, s, t_0) = p_0(n, s)$. We denote initial conditions centered on a deterministic state as $N(t_0) = n_0$ and $S(t_0) = s_0$.

As example simulation is shown in figure 2. All figures in the document are generated via our python software available at [1] which allows to regenerated each of them.

1.1 Master equation

Note that the probability for more than one event to happen in the time interval dt is $\mathcal{O}(dt)$. Thus given a state (n, s) at time t and denoting by $q_{n,s\to m,r}$ the transition probability to change to a state (m, r) at $t + dt$ we can list all the single events that may happen:

$$\begin{aligned}
q_{n,s\to n+1,s} &= \rho_{\emptyset,n} dt + \mathcal{O}(dt) \\
q_{n,s\to n-1,s} &= n\gamma_{n,d} dt + \mathcal{O}(dt) \\
q_{n,s\to n-1,s+1} &= n\gamma_{n,s} dt + \mathcal{O}(dt) \\
q_{n,s\to n,s-1} &= s\gamma_{s,d} dt + \mathcal{O}(dt)
\end{aligned}$$

and for no event to happen

$$q_{n,s\to n,s} = 1 - q_{n,s\to n+1,s} - q_{n,s\to n-1,s} - q_{n,s\to n-1,s+1} - q_{n,s\to n,s-1}$$

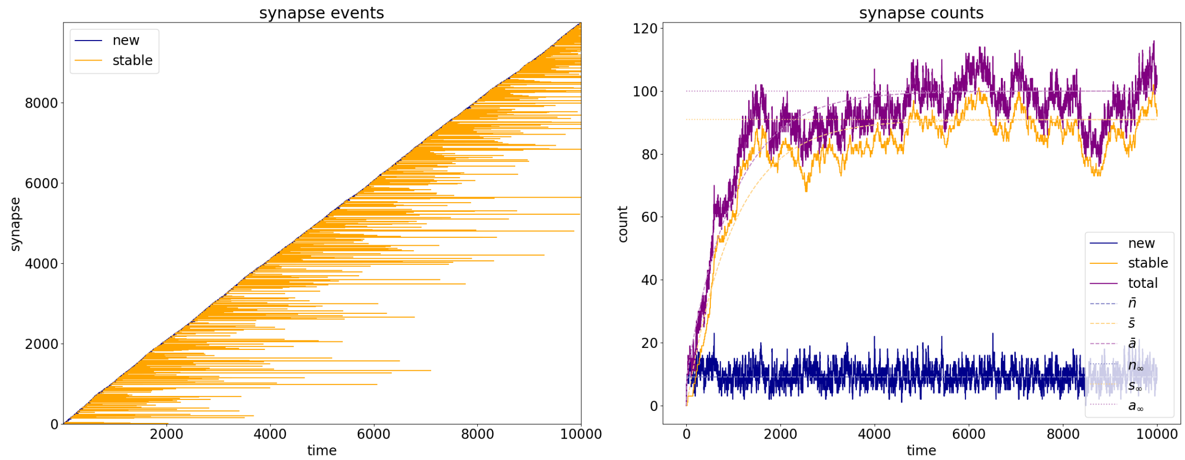

Figure 2: Simulation of the model for $\theta = (1, 0.1, 0.01, 0.001)$. **(a)** Synapse dynamics in the model indicating the state for each synapse over time (new in blue, stable in orange). **(b)** Number of new (blue), stable (orange), and total (purple) synapses over time together with the mean (dashed, cf. eq. (2)) and steady state (dotted, cf. eq. (4)) predictions.

Hence, the probabilities $p\left(n, s, t+dt\right)$ for the system to be in a state (n, s) at a time $t + dt$ are given by:

$$\begin{aligned} p(n, s, t+dt) =& q_{n,s\to n,s} p(n, s, t) + q_{n-1,s\to n,s} p(n-1, s, t) + q_{n+1,s\to n,s} p(n+1, s, t) \\ &+ q_{n+1,s-1\to n,s} p(n+1, s-1, t) + q_{n,s+1\to n,s} p(n, s+1, t) + \mathcal{O}\left(dt\right) \end{aligned}$$

We can rearrange and and take the limit $dt \to 0$ to obtain the master equations:

$$\begin{aligned} \partial_t p(n, s, t) =& -\left(\rho_{\emptyset,n} + n\gamma_{n,d} + n\gamma_{n,s} + s\gamma_{s,d}\right) p(n, s, t) + \rho_{\emptyset,n} p(n-1, s, t) + (n+1)\gamma_{n,d} p(n+1, s, t) \\ &+ (n+1)\gamma_{n,s} p(n+1, s-1, t) + (s+1)\gamma_{s,d} p(n, s+1, t) \end{aligned} \tag{1}$$

1.2 Average dynamics

For the average number of new $\bar{n}$ and stable $\bar{s}$ synapses we have

$$\begin{aligned} \bar{n}(t) &= \mathbb{E}\left(N(t)\right) = \sum_n n p(n, t) \\ \bar{s}(t) &= \mathbb{E}\left(S(t)\right) = \sum_s s p(s, t) \end{aligned} \tag{2}$$

Using (2), we can derive their evolution equations from the master equations (1) as:

$$\begin{aligned} \partial_t \bar{n}(t) &= \rho_{\emptyset,n} - \bar{n}(t)\gamma_{n,-} \\ \partial_t \bar{s}(t) &= \bar{n}(t)\gamma_{n,s} - \bar{s}(t)\gamma_{s,d} \end{aligned}$$

which for the initial conditions

$$\bar{n}(t_0) = \bar{n}_0 \qquad \bar{s}(t_0) = \bar{s}_0$$

can be integrated to yield

$$\begin{aligned} \bar{n}(t) &= \bar{n}_\infty + \left(\bar{n}_0 - \bar{n}_\infty\right) e^{-\gamma_{n,-}(t-t_0)} \\ \bar{s}(t) &= e^{-\gamma_{s,d}(t-t_0)} \left(\bar{s}_0 + \int_{t_0}^{t} e^{\gamma_{s,d}(s-t_0)} \gamma_{n,s} \bar{n}(s) ds\right) \end{aligned} \tag{3}$$

with steady state solution $(t \to \infty)$

$$\bar{n}_\infty = \frac{\rho_{\emptyset,n}}{\gamma_{n,-}} \qquad \bar{s}_\infty = \bar{n}_\infty \frac{\gamma_{n,s}}{\gamma_{s,d}} = \frac{\rho_{\emptyset,n}}{\gamma_{n,-}} \frac{\gamma_{n,s}}{\gamma_{s,d}} \tag{4}$$

We can solve for s explicitly

$$\bar{s}(t) = \bar{s}_\infty + (\bar{s}_0 - \bar{s}_\infty) e^{-\gamma_{s,d}(t-t_0)} + (\bar{n}_0 - \bar{n}_\infty) \frac{\gamma_{n,s}}{\gamma_{n,-} - \gamma_{s,d}} \left(e^{-\gamma_{s,d}(t-t_0)} - e^{-\gamma_{n,-}(t-t_0)} \right)$$

with a direct interpretation: The first term is the steady state, the second term captures initial deviations of stable synapses from the steady state while the last term captures deviations in the new state which first transition to the stable state and then decay form there.

The results of this section are illustrated in figure 2. This concludes the derivation of the population and average dynamics for the synapses which we can use to derive expressions for the average observed synapse numbers in experiments.

1.3 Average measurements

1.3.1 Experimental measures

Experimentally we cannot monitor the synapses continuously, and thus only measure them a discrete points in time. We here define the observables that we used to measure the synapse dynamics experimentally and connect them to our model.

More concretely, we measure occurrences of synapses at $K \in \mathbb{N}$ different measurement times t_k, $k \in \{1, 2, \ldots, K\}$ with $t_k < t_{k+1}$. We denote the set of all measurement times by

$$\mathcal{T} = \{t_1, \ldots, t_K\}$$

For later mathematical convenience we further define measurement times $t_0 = -\infty$ and $t_{K+1} = \infty$ to absorb boundary cases and discuss their role below. For the experiments in the main text we have 6 measurement times with $\mathcal{T} = \{0\text{h}, 6\text{h}, 12\text{h}, 24\text{h}, 48\text{h}, 72\text{h}\}$.

In the experiment it is determined at which measurement time t_f a synapse *first* appeared (i.e. it was not observed at the previous measurement time t_s) as well as at what measurement time t_l it was seen *last* (i.e. it *disappeared* at the following measurement time t_e). We denote the total *number of synapses that occurred between* t_f *and* t_l as

$$O_{s,f,l,e} = O(t_s, t_f, t_l, t_e) \tag{5}$$

where we emphasized that this number depends on four time points (*start, first, last, end*). To simplify notation we also write

$$O_{f,l} = O_{f-1,fl,l+1}$$

for a given $\mathcal{T}$. From this, the *total number of all synapses* at a single observation time t_k denoted by $A_k = A(t_k)$ can be derived as:

$$A_k = \sum_{f=1}^{k} \sum_{l=k}^{K} O_{f,l}$$

The synapses that *appear* between t_s and t_f are synapses that are not observed at t_s and *become visible* at t_f. We denoted their number by $B_{s,f} = B(t_s, t_f)$. We also write $B_f = B_{f-1,f}$ and we have

$$B_f = \sum_{l=f}^{K} O_{f,l} \tag{6}$$

Finally, we define the number of synapses that first become visible between t_s and t_f and *continue to be visible* until at least $t_l \geq t_f$ as $C_{s,f,l} = C(t_s, t_f, t_l)$. We use $C_{f,l} = C_{f-1,f,l}$ and have

$$C_{f,l} = \sum_{k=l}^{K} O_{f,k} \leq C_{f,f} \tag{7}$$

For a fixed f, the measures $C_{f,l}$ as a function of $l \geq k$ this measure has the interpretation of a experimentally sampled survival curve for the synapses first observed at t_f and thus have a intuitive interpretation. To highlight differences between our models fitted to data below we also define the normalized empirical survival curves as

$$\tilde{C}_{f,l} = \frac{C_{f,l}}{C_{f,f}}$$

where we assume $C_{f,f} \neq 0$ for simplicity. This curves measure the percentage of synapses first observed at t_f and surviving until t_l.

To obtain the synapses observed between t_f and t_l, $O_{f,l}$ we can invert the relationship 7 to obtain

$$O_{f,l} = C_{f,l} - C_{f,l+1} \tag{8}$$

where for convenience we have set $C_{f,K+1} = 0$. Thus, deriving the measures $C_{f,l}$ from our model will enable us to fit it to our experimental observations $O_{f,l}$. While the measures $O_{f,l}$ are less easy to interpret, the advantage of using the measures $O_{f,l}$ is, that they are *independent* random variables which simplifies the statistical inference used to estimate model parameters as will be shown below.

We also define the number of *decayed* synapses in the measurement interval (t_l, t_e) denoted by $D_{l,e} = D(t_l, t_e)$. Denoting $D_l = D_{l,l+1}$ we have as

$$D_l = \sum_{k=1}^{l} O_{k,l}$$

1.3.2 Model predictions

Here we will use the average developmental synapse dynamics (3) to predict the average numbers of observed synapses. As we have no experimental access to the internal state of the synapses at any instance of time t_k, the total number of synapses we observe is given by

$$A_k = A(t_k) = N(t_k) + S(t_k)$$

and on average

$$\bar{a}_k = \bar{a}(t_k) = \bar{n}(t_k) + \bar{s}(t_k)$$

where $\bar{n}$ and $\bar{s}$ are given by (3) and depend on initial conditions. Under the assumption that the system is in a steady state this reduces to

$$\bar{a}_k = \bar{n}_\infty + \bar{s}_\infty = \bar{a}_\infty$$

independent of the observation time t_k and initial synapse counts.

For the number of synapses $B_{s,f}$ that become visible for the first time at t_f and thus were not observed at the previous observation time t_s we can use (3) with $n_0 = s_0 = 0$ and $t_0 = t_s$, $t = t_f$ to obtain for the average appearance of new and stable synapses

$$\begin{aligned} \bar{b}^{(n)}_{s,f} &= \bar{n}_\infty \left(1 - e^{--\gamma_{n,-}(t_f - t_s)}\right) \\ \bar{b}^{(s)}_{s,f} &= \bar{s}_\infty \left(1 - e^{-\gamma_{s,d}(t_f - t_s)}\right) - \bar{n}_\infty \frac{\gamma_{n,s}}{\gamma_{n,-} - \gamma_{s,d}} \left(e^{-\gamma_{s,d}(t_f - t_s)} - e^{-\gamma_{n,-}(t_f - t_s)}\right) \end{aligned} \tag{9}$$

and thus in total

$$\bar{b}_{s,f} = \bar{b}^{(n)}_{s,f} + \bar{b}^{(s)}_{s,f}$$

Note that for $1 \leq s$ these observations of appearing synapses do not depend on the initial state or the assumption of a steady state of the model and are thus very useful types of observations for calibrating the model.

We can further calculate the change in the number of synapses that appeared at t_f and remain visible in a subsequent observation at time t_l. As we focus on the already appeared synapses at t_f, we can ignore newly generated synapses after t_f. In this case we can set $\rho_{\emptyset,n} = 0$ in our model to describe the dynamics and use solutions (2) for the average dynamics. We have $\bar{n}_\infty = \bar{s}_\infty = 0$ in this case as all synapses will decay eventually and our initial conditions are given by the average number of synapses appearing at t_f, (9), i.e. $n_0 = \bar{b}^{(n)}_{s,f}$ and $s_0 = \bar{b}^{(s)}_{s,f}$. This results in an average number of remaining synapses in the new and stable states given by

$$\begin{aligned} \bar{c}^{(n)}_{s,f,l} &= \bar{b}^{(n)}_{s,f} e^{-\gamma_{n,-}(t_l - t_f)} \\ \bar{c}^{(s)}_{s,f,l} &= \bar{b}^{(s)}_{s,f} e^{-\gamma_{s,d}(t_l - t_f)} + \bar{b}^{(n)}_{s,f} \frac{\gamma_{n,s}}{\gamma_{n,-} - \gamma_{s,d}} \left(e^{-\gamma_{s,d}(t_l - t_f)} - e^{-\gamma_{n,-}(t_l - t_f)}\right) \end{aligned} \tag{10}$$

and for the average total number of remaining synapses

$$\begin{aligned}\bar{c}_{s,f,l} =& \bar{c}^{(n)}_{s,f,l} + \bar{c}^{(s)}_{s,f,l} \\ =& \bar{n}_\infty \left(e^{-\gamma_{n,-}(t_l - t_f)} - e^{-\gamma_{n,-}(t_l - t_s)}\right) + \bar{s}_\infty \left(e^{-\gamma_{s,d}(t_l - t_f)} - e^{-\gamma_{s,d}(t_l - t_s)}\right) \\ &+ \bar{n}_\infty \frac{\gamma_{n,s}}{\gamma_{n,-} - \gamma_{s,d}} \left(e^{-\gamma_{s,d}(t_l - t_f)} - e^{-\gamma_{s,d}(t_l - t_s)} - e^{-\gamma_{n.-}(t_l - t_f)} + e^{-\gamma_{n,-}(t_l - t_s)}\right)\end{aligned} \tag{11}$$

These average expressions for the observations in the model can now be used to fit the model parameter θ to the experimental data. In particular, we can connect the results here to the experimental observations $O_{s,f,l,e}$ using (8). On average

$$\bar{o}_{s,f,l,e} = \bar{c}_{s,f,l} - \bar{c}_{s,f,e} \tag{12}$$

which are independent of any additional assumptions for $1 \leq s$. Like the total number of synapses $\bar{m}_k$, the initial observations $\bar{o}_{0,f,l,e}$ will depend on additional assumptions about the initial state. Under the steady state assumption we can take the limit $t_0 \to -\infty$ as indicated above.

1.4 Measurement distributions

Here we derive the distributions for the number $O_{f,l}$ of occurred synapses (5) given a set of measurement times $\mathcal{T}$. For this, it is sufficient to consider four arbitrary measurement times $t_s \leq t_f \leq t_l \leq t_e$ (*start, first, last, end*) and derive the probability distribution for the number of occurred synapses

$$p\left(o|t_s, t_f, t_l, t_e\right) = \mathbb{P}\left(O\left(t_s, t_f, t_l, t_e\right) = o\right) \tag{13}$$

The main result of this section is that all measures described above follow a Poisson distribution with means as derived above and as expected by intuition. We will use these results to perform statistical inference on the model parameters. For completeness, we here include a full analytical derivation of the measurement distributions for the mathematically inclined reader.

Specifically, we will derive the probability distribution (12) in three steps. First, we consider the distribution of synapses that appeared for the first time at t_f but were not observed at the previous measurement time t_s. Then we consider how this distribution changes for synapses that are continued to be observed (7) at a later time t_l. Finally, we determine which of these synapses decay between t_f and the final time t_e. In order to facilitate the calculations we first derive probabilities of single synapses remaining in their state or transitioning between certain states.

1.4.1 Single synapse transition probabilities

Here we derive the probabilities $w_{x\to y}(t_1, t_0)$ for a single synapse to transition from a state x at time t_0 to another state y at time t_1. Assuming a synapse is a state x at time t we denote the probabilities to transition to the state y in a short time interval dt as $q_{x\to y}$. For sufficiently small dt we have for new synapses

$$\begin{aligned} q_{n\to n} &= (1 - \gamma_{n,-} dt) + \mathcal{O}(dt) \\ q_{n\to s} &= \gamma_{n,s} dt + \mathcal{O}(dt) \\ q_{n\to d} &= \gamma_{n,d} dt + \mathcal{O}(dt) \end{aligned}$$

Thus, the probability for a single new synapse at time t_0 to remain in the new state until some time $t \geq t_0$ is

$$w_{n\to n}(t + dt, t_0) = q_{n\to n} w_{n\to n}(t, t_0) + \mathcal{O}(dt)$$

and in the limit $dt \to 0$ we obtain

$$\partial_t w_{n\to n}(t, t_0) = -\gamma_{n,-} w_{n\to n}(t, t_0)$$

with $w_{n\to n}(t_0, t_0) = 1$. The solution is given by

$$w_{n\to n}(t_1, t_0) = e^{-\gamma_{n,-}(t_1 - t_0)} \tag{14}$$

Similarly, for the probability for a new synapse to decay without switching to the stable state we have

$$w_{n\to d}(t + dt, t_0) = q_{n\to d} w_{n\to n}(t, t_0) + w_{n\to d}(t, t_0)$$

which gives

$$\partial_t w_{n\to d}(t,t_0) = \gamma_{n,d} w_{n\to n}(t,t_0)$$

with $w_{n\to d}(t_0,t_0) = 0$. Using the result above for $p_{n\to n}(t,t_0)$ this can be integrated to

$$w_{n\to d}(t_1,t_0) = \frac{\gamma_{n,d}}{\gamma_{n,-}}\left(1 - e^{-\gamma_{n,-}(t_1-t_0)}\right) \tag{15}$$

Similarly, for stable synapses we can derive

$$\begin{aligned} q_{s\to d} &= \gamma_{s,d}dt + \mathcal{O}(dt) \\ q_{s\to s} &= 1 - q_{s\to d} \end{aligned}$$

and obtain

$$w_{s\to s}(t_1,t_0) = e^{-\gamma_{s,d}(t_1-t_0)} \tag{16}$$

$$w_{s\to d}(t_1,t_0) = 1 - e^{-\gamma_{s,d}(t_1-t_0)} \tag{17}$$

For the transition probability of a new synapse at t_0 to transition to a stable synapse at time t, $w_{n\to s}(t,t_0)$ we have

$$w_{n\to s}(t+dt,t_0) = q_{n\to s}w_{n\to n}(t,t_0) + q_{s\to s}w_{n\to s}(t,t_0)$$

and taking limits we obtain

$$\partial_t w_{n\to s}(t,t_0) = \gamma_{n,s}w_{n\to n}(t,t_0) - \gamma_{s,d}w_{n\to s}(t,t_0)$$

with $w_{n\to s}(t_0,t_0) = 0$ and with solution

$$w_{n\to s}(t_1,t_0) = \frac{\gamma_{n,s}}{\gamma_{n,-} - \gamma_{s,d}}\left(e^{-g_{s,d}(t_1-t_0)} - e^{-g_{n,-}(t_1-t_0)}\right) \tag{18}$$

Finally, for the probability to first stabilize and then decay we have

$$w_{n\to s\to d}(t+dt,t_0) = q_{s\to d}w_{n\to s}(t,t_0) + w_{n\to s\to d}(t,t_0)$$

which yields

$$w_{n\to s\to d}(t_1,t_0) = \frac{\gamma_{n,s}}{\gamma_{n,-}} - \frac{\gamma_{n,s}\gamma_{s,d}}{\gamma_{n,-} - \gamma_{s,d}}\left(\frac{1}{\gamma_{s,d}}e^{-\gamma_{s,d}(t_1-t_0)} - \frac{1}{\gamma_{n,-}}e^{-\gamma_{n,-}(t_0-t_1)}\right) \tag{19}$$

A new synapse will take one of all it's possible paths and thus we have

$$1 = w_{n\to n}(t_1,t_0) + w_{n\to d}(t_1,t_0) + w_{n\to s}(t_1,t_0) + w_{n\to s\to d}(t_1,t_0)$$

which can also be easily verified from the results above.

1.4.2 Measurement distributions

We denote the distribution of the number $B_{s,f} = B(t_s,t_f)$ of synapses becoming visible between t_s and t_f as

$$p(b|t_s,t_f) = \mathbb{P}\left(B(t_s,t_f) = b\right)$$

As we cannot discriminate between the synapse's internal states in our experimental observations, the measured number $B_{s,f}$ is composed of new and stable synapses,

$$B_{s,f} = B_{s,f}^{(n)} + B_{s,f}^{(s)}$$

Lets consider $B_{s,f}^{(n)}$ first. To observe a single new synapse at t_f not previously observed at t_s it needs to be generated within the time interval (t_s,t_f) and not decay or transition until t_f. Thus, we consider the number of synapses $G_{s,f}$ generated in (t_s,t_f) irrespective of their future fate. In our model, synapses are generated via a Poisson process with mean

$$\bar{g}_{s,f} = \rho_{\emptyset,n}(t_f - t_s)$$

Moreover, the distribution of their generation times t_g is uniformly distributed on (t_s, t_f)

$$p(t_g) = \frac{1}{(t_f - t_s)}$$

$s \geq 1$To become measurable at t_f a synapse generated at a random time $t_g \in (t_s, t_f)$ also needs to survive and not decay. There are two possibilities to survive: (i) the synapse remains in the new state until t_f, or (ii) the synapse changes to a stable state within (t_s, t_f) and survives as a stable synapse until t_f.

Using our results on the single synapse transition probabilities for new synapses derived in (14) we obtain for the probability of a generated synapse to become visible at t_f as a new synapse

$$w_{g\to n}(t_f, t_s) = \int_{t_s}^{t_f} w_{n\to n}(t_f, t_g) p(t_g)\, dt_g = \frac{1}{t_f - t_s}\frac{1}{\gamma_{n,-}}\left(1 - e^{-\gamma_{n,-}(t_f - t_s)}\right)$$

Thus, out of the generated $g_{s,f}$ synapses each synapses survives independently with this probability. Thus given $g_{s,f}$ synapses the resulting distribution for the new synapses at t_f is binomial distributed with parameter $g_{s,f}$ and $w_{g\to n}(t_f, t_s)$. Using binomial thinning on the Poisson distribution for $G_{s,f}$ we find that the distribution for $B_{s,f}^{(n)}$ is also Poisson with mean

$$\bar{b}_{s,f}^{(n)} = \bar{g}_{s,f} w_{g\to n}(t_f, t_s) = \frac{\rho_{\emptyset,n}}{\gamma_{n,-}}\left(1 - e^{-\gamma_{n,-}(t_f - t_s)}\right) \tag{20}$$

which, as expected, matches our results (9) for the average number of new synapses becoming visible in (t_s, t_f).

We next consider the synapses that transitioned to a stable state before becoming visible at t_f. We can use (18) for a single new synapse at time t_g to be in the stable state at $t_f$and thus similarly as before

$$w_{g\to s}(t_f, t_s) = \int_{t_s}^{t_f} w_{n\to s}(t_f, t_g) p(t_g)\, dt_g = \frac{1}{t_f - t_s}\frac{\gamma_{n,s}}{\gamma_{n,-} - \gamma_{s,d}}\left(\frac{1}{\gamma_{s,d}}\left(1 - e^{-\gamma_{s,d}(t_f - t_s)}\right) - \frac{1}{\gamma_{n,-}}\left(1 - e^{-\gamma_{n,-}(t_f - t_s)}\right)\right)$$

Using binomial thinning on $G_{s,f}$ this gives rise to a Poisson distribution for $B_{s,f}^{(n)}$ with mean

$$\bar{b}_{s,f}^{(s)} = \bar{g}_{s,f} w_{g\to s}(t_f, t_s) = \frac{\rho_{\emptyset,n}\gamma_{n,s}}{\gamma_{n,-} - \gamma_{s,d}}\left(\frac{1}{\gamma_{s,d}}\left(1 - e^{-\gamma_{s,d}(t_f - t_s)}\right) - \frac{1}{\gamma_{n,-}}\left(1 - e^{-\gamma_{n,-}(t_f - t_s)}\right)\right) \tag{21}$$

which again is the mean equivalent to (9).

The number of synapses $B_{s,f}$ becoming visible in the interval (t_s, t_f) is the sum of two independent Poisson distributed random variables, and thus also Poisson distributed with mean

$$\bar{b}_{s,f} = \bar{b}_{s,f}^{(n)} + \bar{b}_{s,f}^{(s)} \tag{22}$$

We now consider the distribution of synapses that continued to be visible until a last measurement time t_l. In order for a synapse to become visible at t_f and also continue to be visible at t_l it needs to survive the time interval (t_f, t_l). We consider new and stable synapses at t_f separately.

Stable synapses can only survive by not decaying. We derived the number $B_{s,f}^{(s)}$ of stable synapses at t_f to be Poisson distributed in (21) and derived their survival probability in (16). In total, the number $C_{s,f,l}^{(s)}$ of stable synapses at t_l that became visible at t_f is also Poisson distributed with mean

$$\begin{aligned}\bar{c}_{s,f,l}^{(s\to s)} &= \bar{b}_{s,f}^{(s)} w_{s\to s}(t_l, t_f) \\ &= \frac{\rho_{\emptyset,n}\gamma_{n,s}}{\gamma_{n,-} - \gamma_{s,d}}\left(\frac{1}{\gamma_{s,d}}\left(1 - e^{-\gamma_{s,d}(t_f - t_s)}\right) - \frac{1}{\gamma_{n,-}}\left(1 - e^{-\gamma_{n,-}(t_f - t_s)}\right)\right) e^{-\gamma_{s,d}(t_l - t_f)}\end{aligned}$$

New synapses may survive until t_l as new synapses $C_{s,f,l}^{(n\to n)}$or transition to stable synapses and survive as such $C_{s,f,l}^{(n\to s)}$. For the first case we have $B_{s,f}^{(n)}$ new synapses at t_f and each of them survives with probability given by (14). As $B_{s,f}^{(n)}$ is Poisson $C_{s,f,l}^{(n\to n)}$ is Poisson with mean

$$\bar{c}_{s,f,l}^{(n\to n)} = \bar{b}_{s,f}^{(n)} w_{n\to n}(t_l, t_f) = \frac{\rho_{\emptyset,n}}{\gamma_{n,-}}\left(1 - e^{-\gamma_{n,-}(t_f - t_s)}\right) e^{-\gamma_{n,-}(t_l - t_f)}$$

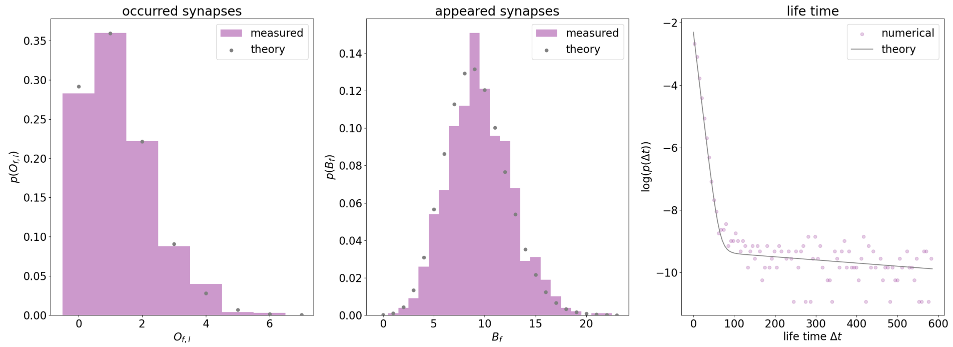

Figure 3: Measurement distributions for the example simulation in figure 2. **(a)** Occurred synapses $O_{s,f,l,e}$ measured and predicted via (23) for $t_f = t_s + 10$, $t_l = t_s + 10$, $t_e = t_e + 10$ and 500 choices of t_s sampled uniformly across the simulation interval. **(b)** As in (a) for synapses becoming visible, $B_{s,f}$, measured and predicted via (22) for $t_f = t_s + 20$. **(c)** Lifetime distribution $p(\Delta t)$ measured from the simulation data and predicted via (24). Note that to better identify the two time scales a logarithmic y-axis is used.

Similarly $C^{(n\to s)}_{s,f,l}$ is derived to be Poisson with mean

$$\bar{c}^{(n\to s)}_{s,f,l} = \bar{b}^{(n)}_{s,f} w_{n\to s}(t_l, t_f) = \frac{\rho_{\emptyset,n}}{\gamma_{n,-}}\left(1 - e^{-\gamma_{n,-}(t_f - t_s)}\right)\frac{\gamma_{n,s}}{\gamma_{n,-} - \gamma_{s,d}}\left(e^{-g_{s,d}(t_l - t_f)} - e^{-g_{n,-}(t_l - t_f)}\right)$$

In total we have $C^{(n)}_{s,f,l} = C^{(n\to n)}_{s,f,l}$ new and $C^{(s)}_{s,f,l} = C^{(n\to s)}_{s,f,l} + C^{(s\to s)}_{s,f,l}$ stable synapses at t_f that became visible at t_f as either type of synapse. Both Poisson distributed with corresponding means. Finally, we can measure $C_{s,f,l} = C^{(n)}_{s,f,l} + C^{(s)}_{s,f,l}$ which is Poisson distributed with mean

$$\bar{c}_{s,f,l} = \bar{c}^{(n\to n)}_{s,f,l} + \bar{c}^{(n\to s)}_{s,f,l} + \bar{c}^{(s\to s)}_{s,f,l} = \bar{c}^{(n)}_{s,f,l} + \bar{c}^{(s)}_{s,f,l}$$

which coincides with (11).

Finally, it follows from (8) that the observations $O_{s,f,l,e}$ are Poisson distributed with mean

$$\bar{o}_{s,f,l,e} = \bar{c}_{s,f,l} - \bar{c}_{s,f,e} \tag{23}$$

Lets derive the last statement. Consider $O_{s,f,l,e}$. Given $C^{(n)}_{s,f,l}$ new and $C^{(s)}_{s,f,l}$ stable synapses at t_l we need to calculate the probability for the number of those that decay until the final observation time t_e. For the $C^{(s)}_{s,f,l}$ stable synapses there is only one way to decay and given that $C^{(n)}_{s,f,l}$ is Poisson combined with (17) we have that $O^{(s\to d)}_{s,f,l,e}$ is Poisson with mean

$$\bar{o}^{(s\to d)}_{s,fl,e} = \bar{c}^{(s)}_{s,f,l} w_{s\to d}(t_e, t_l)$$

For the new synapses $C^{(n)}_{s,f,l}$ at t_l there are two ways to decay, directly form the new state (15) and through the stable state (19). We have

$$\bar{o}^{(n\to d)}_{s,fl,e} = \bar{c}^{(n)}_{s,f,l} w_{n\to d}(t_e, t_l)$$

and

$$\bar{o}^{(n\to s\to d)}_{s,fl,e} = \bar{c}^{(n)}_{s,f,l} w_{n\to s\to d}(t_e, t_l)$$

In total, it follows that $O_{s,f,l,e}$ is the sum of three independent Poisson variables and thus itself Poisson distributed, which is our main result of this section. The mean is given by

$$\bar{o}_{s,f,l,e} = \bar{o}^{(n\to d)}_{s,fl,e} + \bar{o}^{(n\to s\to d)}_{s,fl,e} + \bar{o}^{(s\to d)}_{s,fl,e}$$

which is identical to the mean derived in (12), as expected. Figure 3 shows an example of the predicted distributions for the number of occurred synapses.

1.5 Lifetime distribution

An interesting measure is the lifetime distribution of synapses which could provide details about the underling learning strategies. We here derive the lifetime distributions for the synapses in our model.

We denote the lifetime of a synapse with Δt and derive its distribution $p(\Delta t)$. Therefore we focus on a single synapse that was generated at time t_g and decayed at time t_d. We have $\Delta t = t_d - t_g$. As our model is homogeneous in time we can set $t_g = 0$ without loss of generality.

There are two ways for a synapse to decay: (i) directly from the new state, (15), or (ii) after transitioning to the stable state (19). From our single synapse transition probability calculations above the probability to decay from the new state is given by (15). Thus the total transition probability to decay is

$$w_{n\rightsquigarrow d}(t, 0) = w_{n\to d}(t, 0) + w_{n\to s\to d}(t, 0)$$

Hence, the probability density to decay at a specific time t given the synapse was generated at $t_g = 0$, is simply the time derivative of this probability and thus

$$p(\Delta t) = \partial_t w_{n\rightsquigarrow d}(t, 0)|_{t=\Delta t} = \left(\gamma_{n,d} - \frac{\gamma_{n,s}\gamma_{s,d}}{\gamma_{n,-} - \gamma_{s,d}}\right) e^{-\gamma_{n,-}\Delta t} + \frac{\gamma_{n,s}\gamma_{s,d}}{\gamma_{n,-} - \gamma_{s,d}} e^{-\gamma_{s,d}\Delta t} \quad (24)$$

An example distribution is show in figure (3).

2 One state model

For the one state model we simply forbid transitions to the stable state by setting $\gamma_{n,s} = 0$ and initially allow no stable synapses, i.e. $s_0 = 0$. It follows that $\bar{s}_\infty = 0$. Inserting these values in the above equations we obtain the predictions for the measures in the one state model.

3 Model fitting

3.1 Experimental data

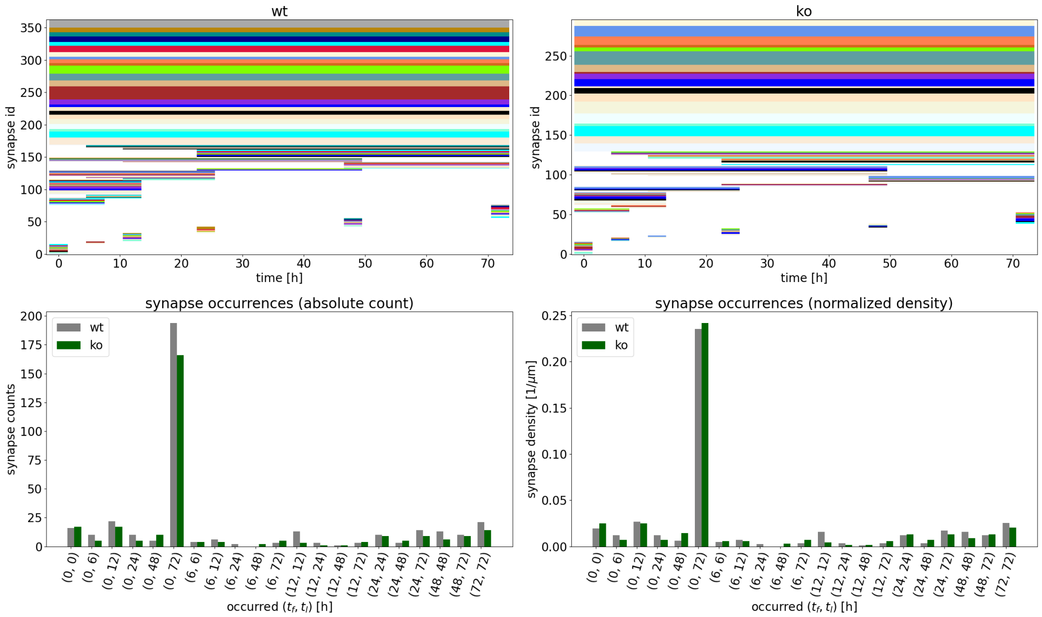

Figure 4: Experimental data. **(a,b)** Measured times of first and last occurrence for each synapse for wild type and knock-out conditions for the 6 measurement times $\mathcal{T} = \{0\text{h}, 6\text{h}, 12\text{h}, 24\text{h}, 48\text{h}, 72\text{h}\}$. Colors indicate synapses of different cells and fish. **(c,d)** Measured number of occurred synapses $O_{f,l}$ for all pair-wise measurement times $\mathcal{T}^2_\leq$ (25) showing raw counts (left) and counts normalized to the total measured dendrite length (right) for wild-type and knock-out conditions.

The experimental data used to fit our model is summarized in figure 4. We consider data from zebrafish hindbrain in wild-type and knock-out of metalloproteinase 14 (MMP14) conditions, as described in the main manuscript. Figure 5 indicates a difference in the distributions of the synapses lifetimes between the wild-type and knock-out condition that show higher survival.

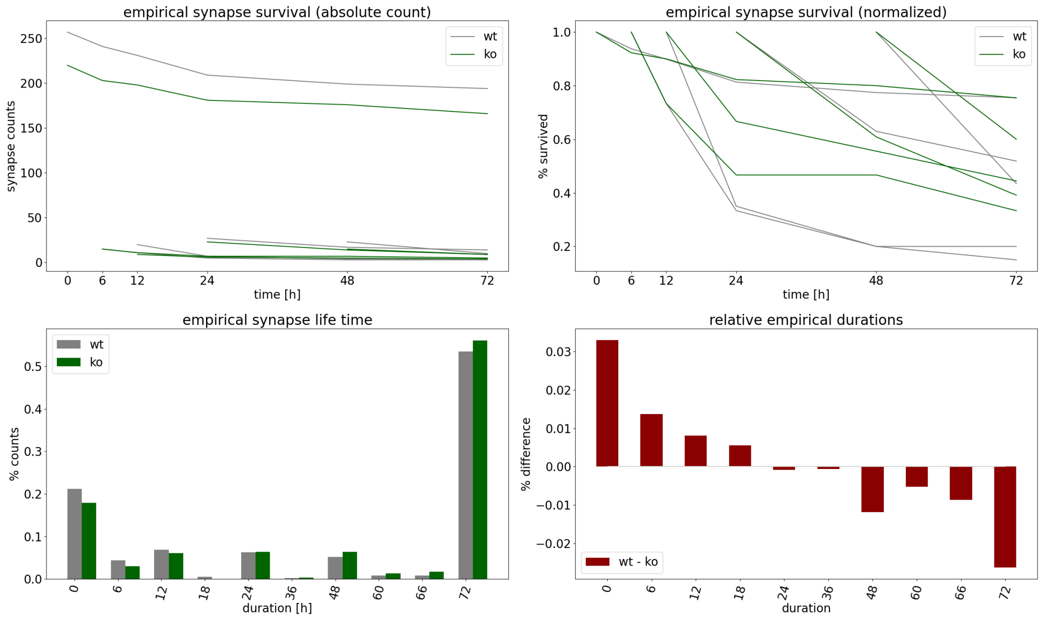

Figure 5: Experimental survival curves and synapse lifetimes. **(a, b)** Measured survival curves showing absolute (a) or normalized number of synapses first seen at the different measurement times for wild-type (gray) and knock-out conditions (green). **(c)** Distribution of experimentally observed synapses lifetimes (normalized to total synapse counts) for wild-type (gray) and knock-out conditions (green). **(d)** Difference in the synapse duration distribution between wild-type and knockout shows a consistent trend for wild-type synapses to live shorter.

3.2 Maximum likelihood estimation

We can perform inference using the distributions for the observations $O_{f,l}$ derived above. To simplify notation, we define for a set of measurement times $\mathcal{T} = \{t_1, \ldots, t_K\}$ with $t_i < t_{i+1}$, the set of ordered measurement time tuples as

$$\mathcal{T}^2_{\leq} = \{(t_f, t_l) \mid t_f, t_l \in T,\ t_f \leq t_l\} \tag{25}$$

For a tuple $m = (t_f, t_l) \in \mathcal{T}^2_{\leq}$ we write for the number of occurred synapses

$$O_m := O_{f,l} = O\left(t_{f-1}, t_f, t_l, t_{l+1}\right)$$

as defined in (5).

Let $\mathcal{S} \subset \mathcal{T}^2_{\leq}$ be a subset of the ordered tuples. We note that for $s \neq r$, the random variables O_s and O_r are independent as they are concerned with different subsets of synapses. Given model parameter θ and experimental observations $\mathcal{O} = \{o_s \mid s \in \mathcal{S}\}$, we can thus derive their joint probability as

$$p\left(\{O_s = o_s \,|\, s \in \mathcal{S}\} \,|\, \theta\right) = \prod_{s \in \mathcal{S}} p(O_s = o_s \,|\, \theta) = \prod_{s \in \mathcal{S}} \frac{(\bar{o}_s)^{o_s}}{o_s!} e^{-\bar{o}_s}$$

using expression (23) for the mean occurred synapses. From this probability, we derive the log likelihood as

$$l\left(\theta\right) = \sum_{s \in \mathcal{S}} \left(o_s \log\left(\bar{o}_s\right) - \bar{o}_s\right) \tag{26}$$

where we dropped parameter independent terms. The maximum likelihood estimator (MLE) is denoted by

$$\hat{\theta} = \arg\max_{\theta} l(\theta) \tag{27}$$

Note that we have the boundary conditions that all parameter are rates and thus are non-negative numbers.

To obtain the maximum likelihood estimator $\hat{\theta}$ we used numerical maximization of (26) using the python scipy optimization library [4]. To double confirm our numerical results we also implemented a Wolfram's Mathematica [5] version of our model were we absorbed the common denominator of all model predictions $\bar{o}_{l,f}$ by optimization for parameters α,$\gamma_{n,d}$, $\gamma_{n,s}$, and $\gamma_{s,d}$ where $\rho_{\emptyset,n} = \alpha\left(\gamma_{n,d}+\gamma_{n,s}\right)\left(\gamma_{n,d}+\gamma_{n,s}-\gamma_{s,d}\right)\gamma_{s,d}$ which avoids any divisions by the model parameters.

Besides the MLE obtained from real data, we calculated standard errors using bootstrapping. Therefore we subsampled the individual synapse observations randomly to 90% of the experimental data, re-normalized the counts of this sample to match the total number of observed synapses and performed inference to estimate the parameter. We repeated this process $B = 100$ times to obtain B estimates of the parameter from which we extracted the standard deviation as a error measure for the MLEs $\hat{\theta}$. We used this measure for the error bars in the figures. We also used the set of B parameters to determine statistically significant differences between the parameter in wild type and knockout conditions using a Mann Whitney U-test an report the resulting p-values.

3.3 Model selection

We tested if a two state model better explains the observed data than a 1-state model. For this we computed the MLEs for the one-state model $\hat{\theta}_{(2)}$ with $k = 2$ parameter and the two-state model $\hat{\theta}_{(4)}$ with $k = 4$ parameter. The results in figure 6 indicate that the two state model explains the data better. Indeed, the likelihoods for the full model is much lower and a comparison between the fitted model predictions and data is shown in figure 7. Also the survival curves are better fit with the two state model (figure 6). This observation is further confirmed using the Akaike information criterion [6] given by

$$\mathrm{AIC}_{(k)} = 2k - 2l\left(\hat{\theta}_{(k)}\right)$$

We find a difference in AIC of $\Delta\mathrm{AIC} = \mathrm{AIC}_{(4)} - \mathrm{AIC}_{(2)} \approx -147$ which estimates the probability for the one state model to fit the data better as $p < 10^{-65}$.

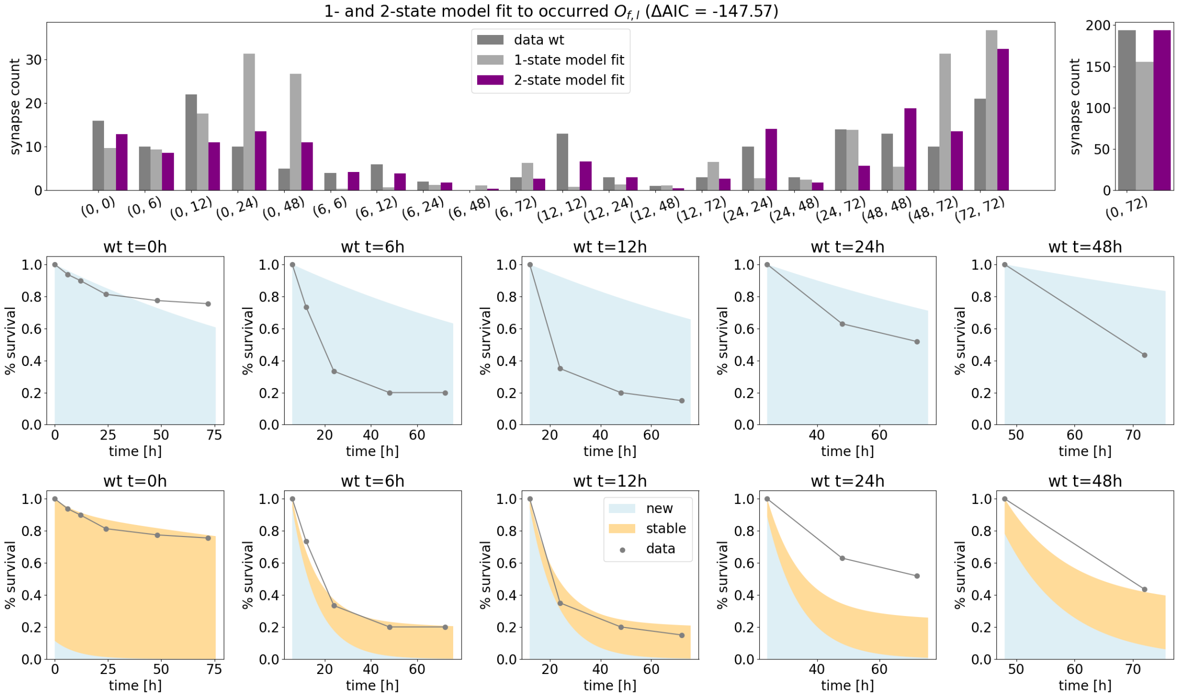

Figure 6: Comparison between one- and two-state models for the wild-type data. **(a)** Fits to the occurred synapse counts $O_{f,l}$ for a 1-state (light gray) and 2-state (purple) model together with the experimental wild-type data (gray). The $\Delta\mathrm{AIC} > -100$ indicates the two state model fits the significantly data better despite 2 more parameters. **(b-f)** The survival curves for the 1-state model (blue shaded) in comparison to the experimentally measured survival. The fast decay of new synapses (e.g. in c,d) is not captured by this model. **(g-l)** Same as in (b-f) but for the fitted 2-state model. Blue shading indicates new (dynamics) synapses while orange shading indicates stable synapses in the model. The faster decay is much better captured by the two state model.

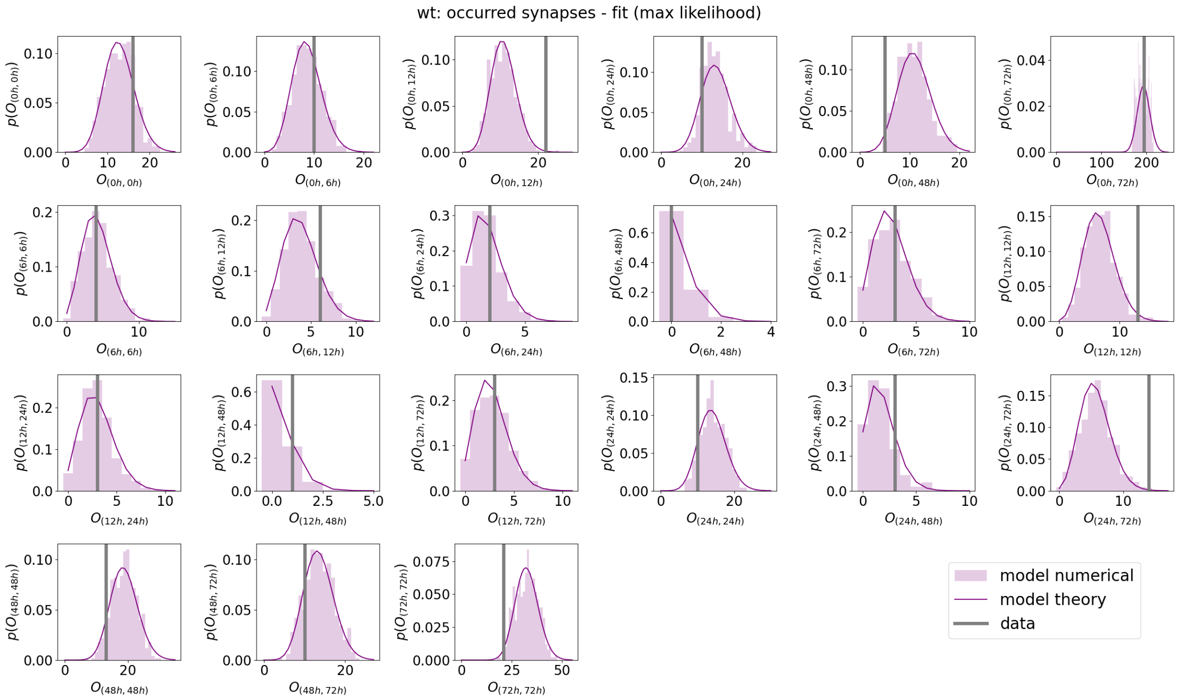

Figure 7: Model predictions and data. Shown are the theoretically derived and numerically simulated distributions for the synapse occurrences $O_{f,l}$ for the 2-state model (purple) using maximum likelihood estimates for the parameters as in figure 6 together with the experimental wild-type data (gray).

Similar results hold for the knock-out data ($\Delta\text{AIC} = \text{AIC}_{(4)} - \text{AIC}_{(2)} \approx -68$, $p < 10^{-29}$) as shown in figure 8 and 9.

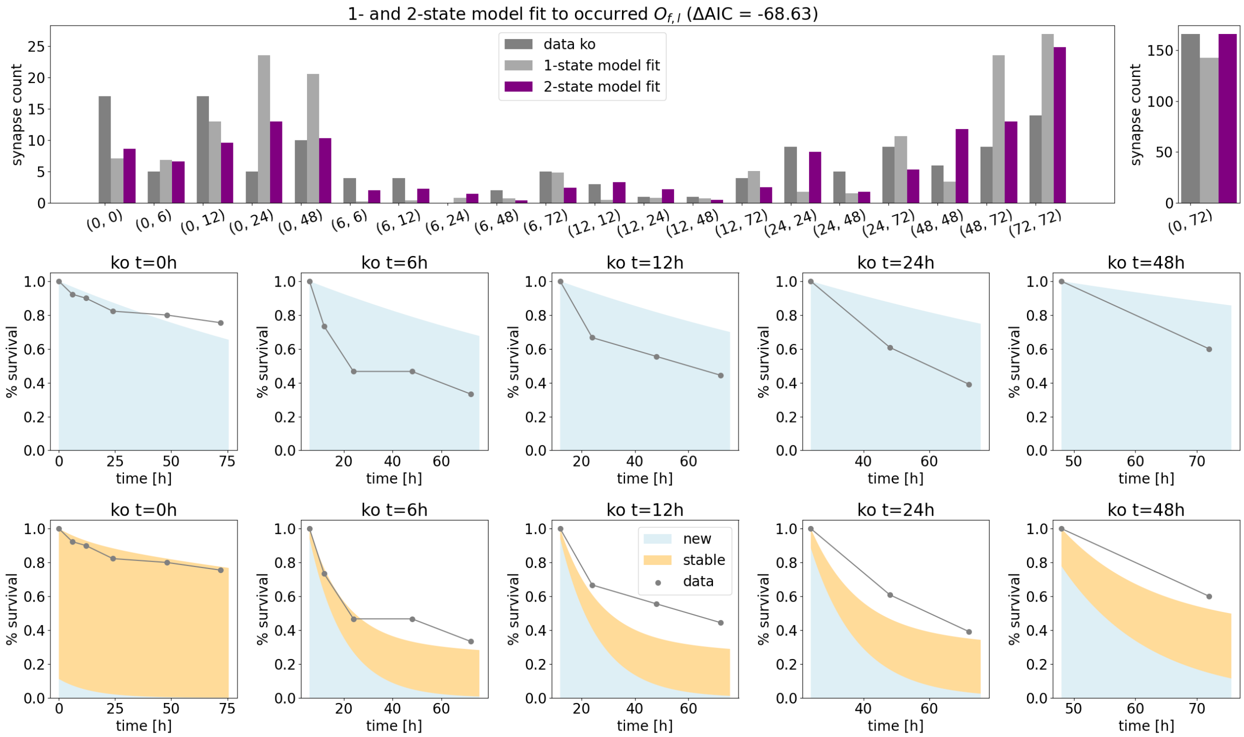

Figure 8: Comparison between one- and two-state models for knock-out data. Panels as in figure 6.

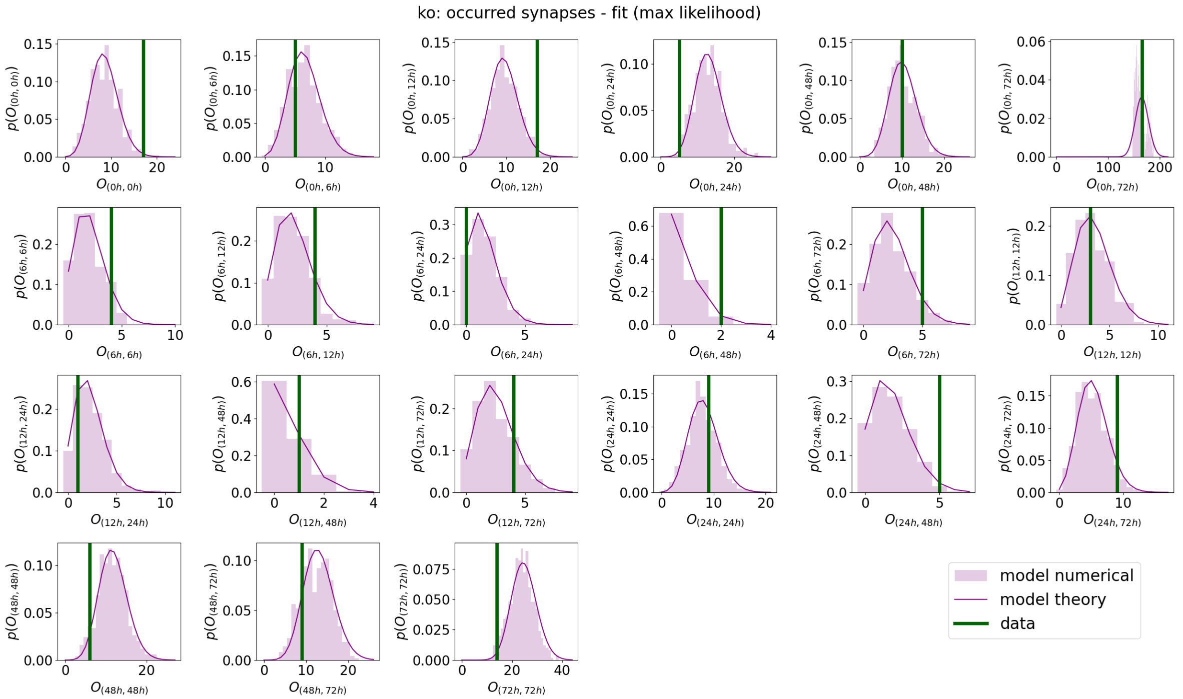

Figure 9: Model predictions and data. Shown are the theoretically derived and numerically simulated distributions for the synapse occurrences $O_{f,l}$ for the 2-state model (purple) using maximum likelihood estimates for the parameters as in figure 8 together with the experimental knock-out condition data (green).

3.4 Model parameter estimation

The results for the model parameter estimation together with statistics is shown in figure 10. We find that there are significant changes in the model parameter, particularly the knock-out model shows a reduced birth and decay rate of new dynamics synapses. This is also reflected in the heaver tail in the synapse lifetime distribution predicted by the model for the knock-out condition (figure 11).

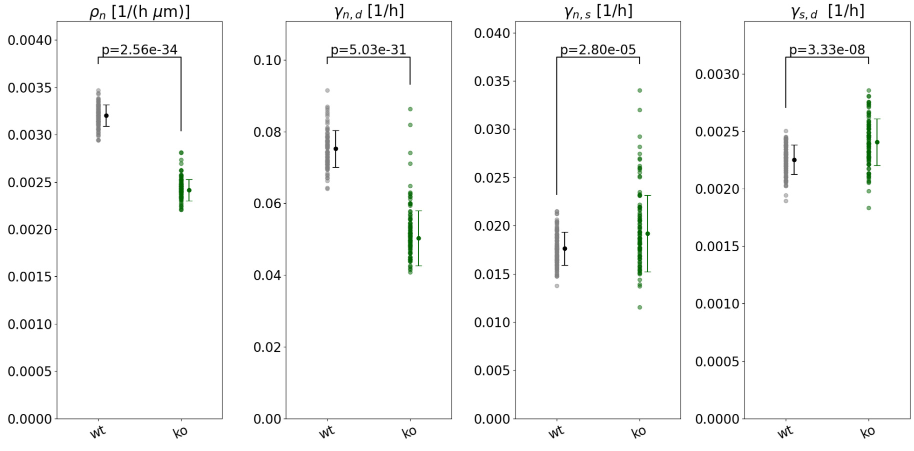

Figure 10: Model parameter comparison. **(a-d)** Maximum likelihood parameter obtained from 100 bootstrap samples ($B = 100$) for wild-type (gray) and knock-out conditions (green) together with the MLE $\hat{\theta}$ (27) obtained from the entire experimental data (dot). Error bars indicate standard deviation of the bootstrapped parameter set. p-values for Mann Whitney U tests are shown.

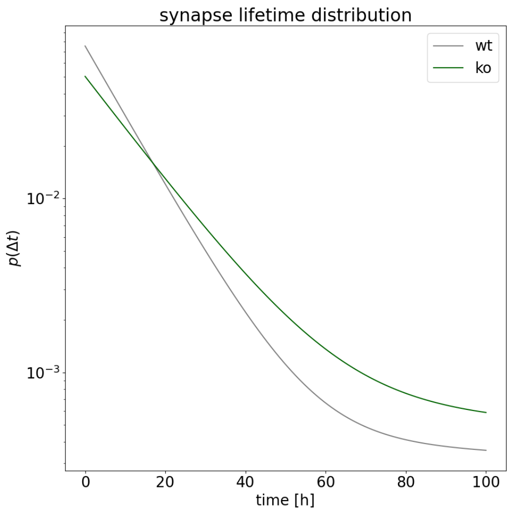

Figure 11: Model lifetime distribution and expected composition of new dynamic and stable synapses Life time distribution 10 for the fitted models shows heaver tail in the knock-out condition as was indicated by the analysis in figure 5.

4 Discussion

Our results indicate that a two state model fits the observed data better compared to a one state model, indicating internal states of synapses that determine their life time. A new unstable state and a stable state are sufficient to explain the observation data well. However, additional internal synapse states or life time dependent synapse stability of synapses may exists beyond the two states we studied here. It is an open question what the role of these states for circuit development and learning and memory are.

The main effects of the knockout in comparison to the wild type are reduced birth and decay rates of new, dynamic synapses.

References

[1] https://github.com/ChristophKirst/developmental_synapse_remodeling 1, 2

[2] Feller, W. (1939). Die Grundlagen der Volterraschen Theorie des Kampfes ums Dasein‘ in wahrscheinlichkeitstheoretischer Behandlung. Acta Biotheoretica. 5 (1): 11–40. doi:10.1007/BF01602932. 1

[3] Cinlar, E. (2013). Introduction to stochastic processes. Courier Corporation. 1

[4] Virtanen P. et al. (2020) SciPy 1.0: Fundamental Algorithms for Scientific Computing in Python. Nature Methods, 17(3), 261-272. DOI: 10.1038/s41592-019-0686-2. 12

[5] Wolfram Research, Inc., Mathematica, Version 14.2, Champaign, IL (2024). 12

[6] Akaike, H. (1998) Information theory and an extension of the maximum likelihood principle. Selected papers of Hirotugu Akaike. Springer New York. pp. 199--213. 12
