# Supplementary material for: Extracellular matrix proteolysis maintains synapse plasticity during brain development
Source: Nat Neurosci. 2025 Dec 22;29(3):567–80. doi: 10.1038/s41593-025-02153-4 (PMC12971489; doi:10.1038/s41593-025-02153-4)
Supplement: Supplementary file 11 — Uncropped western blot of Fig. 4c. [file 41593_2025_2153_MOESM11_ESM.pdf]

Source Data 1: Uncropped Blot of Fig. 4i_BCAN

BCAN

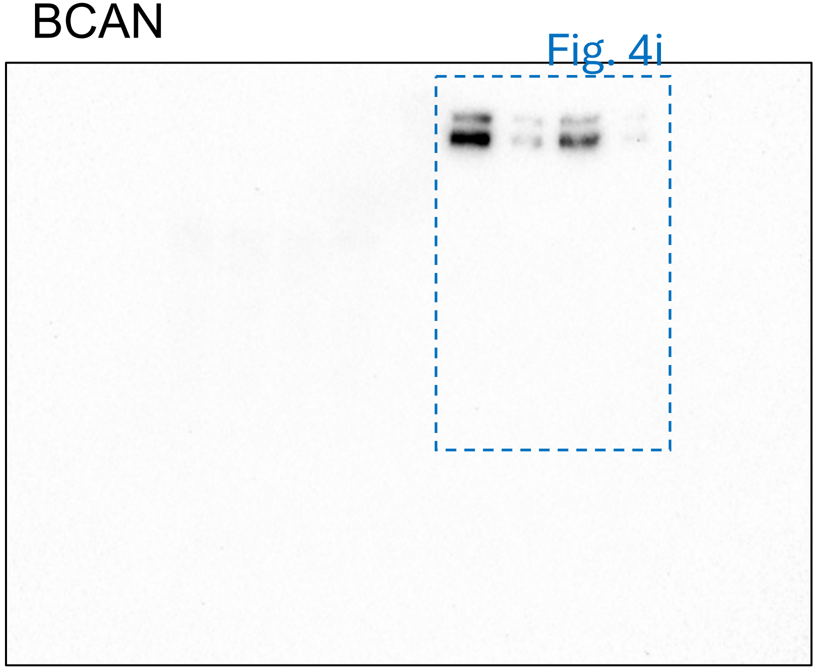

camera

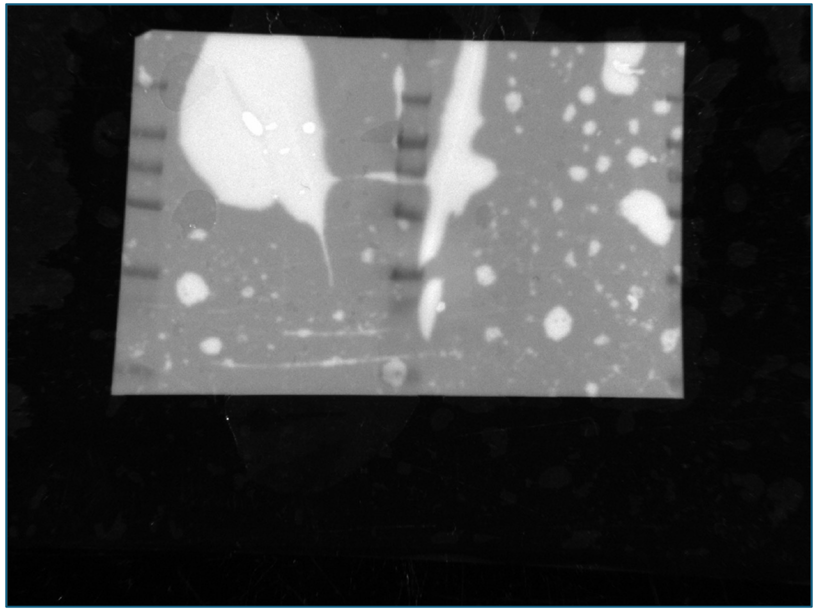

Merge

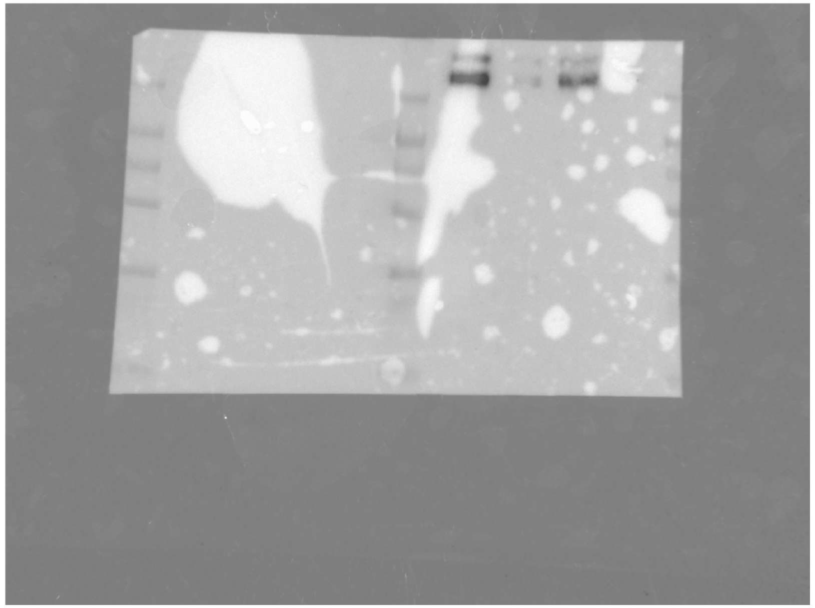

Sample information

	Cell lysate					Supernatant 10X				
marker	no MG	Control MG	shMMP14 MG	shMMP14 MG +MMP14 rescue	marker	no MG	Control MG	shMMP14 MG	shMMP14 MG +MMP14 rescue	marker

Source Data 1: Uncropped Blot of Fig. 4i_MAP2

Cell lysate: MAP2-Ms

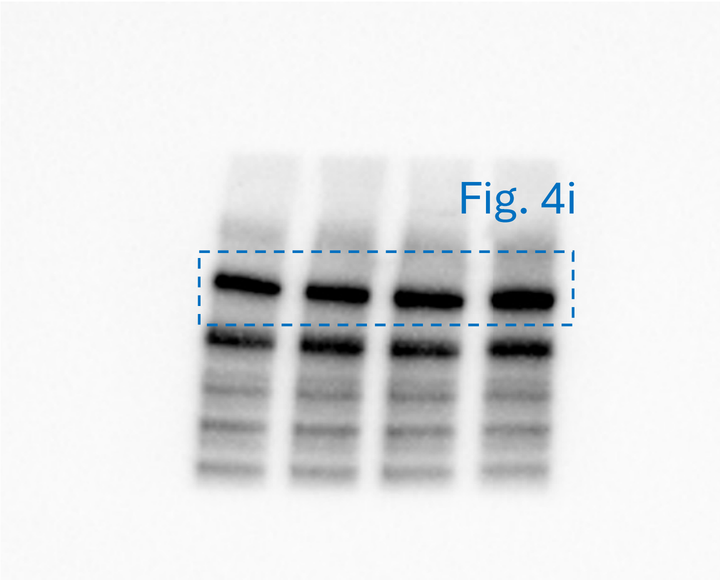

camera

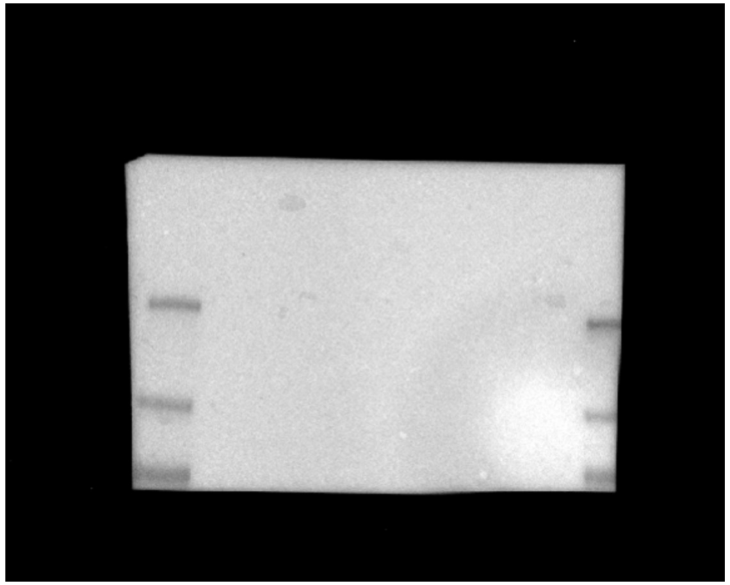

Merge

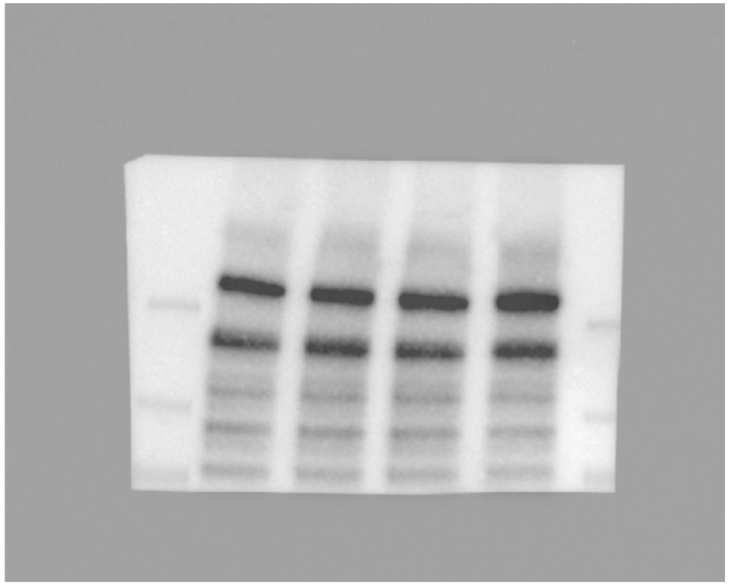

Sample information

	Cell lysate				
marker	no MG	Control MG	shMMP14 MG	shMMP14 MG +MMP14 rescue	marker
