# Supplementary material for: Extracellular matrix proteolysis maintains synapse plasticity during brain development
Source: Nat Neurosci. 2025 Dec 22;29(3):567–80. doi: 10.1038/s41593-025-02153-4 (PMC12971489; doi:10.1038/s41593-025-02153-4)
Supplement: Supplementary file 20 — Uncropped western blot of Extended Data Fig. 8c,g. [file 41593_2025_2153_MOESM20_ESM.pdf]

Source Data 2: Uncropped Blot of Extended Data Fig. 8c

MMP14+Actin

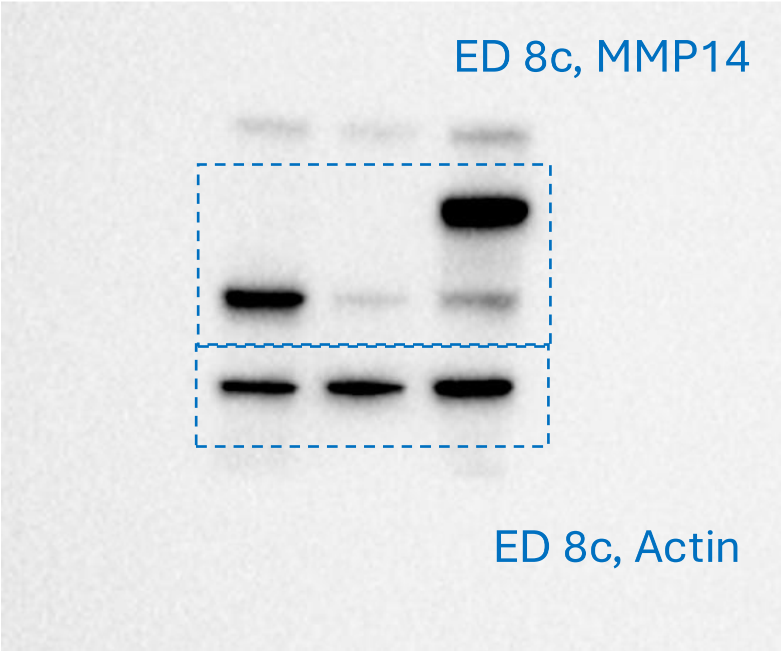

camera

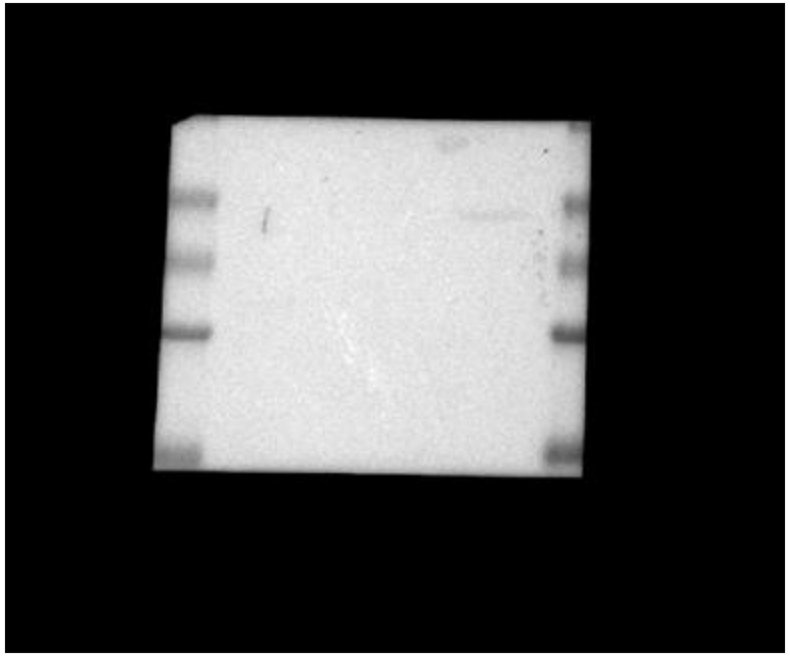

Merge

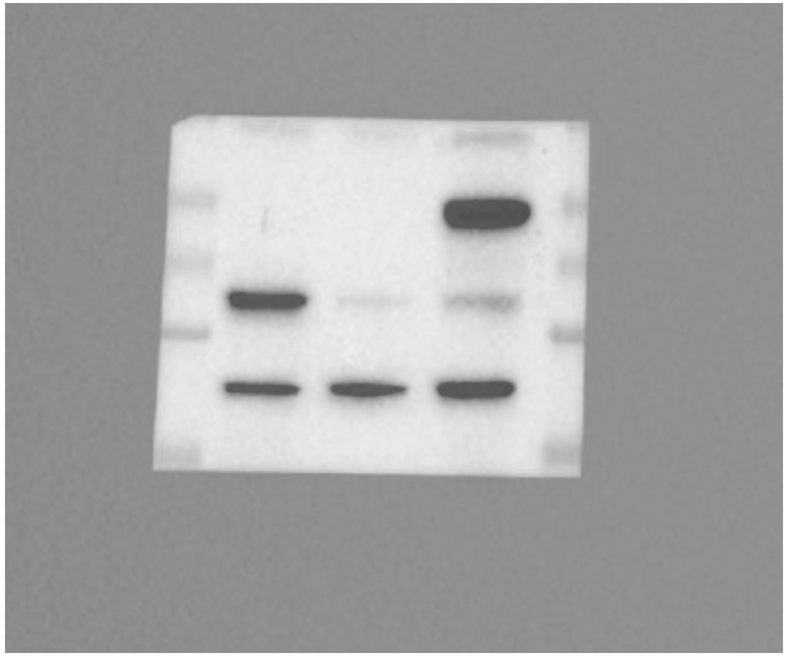

Sample information

marker	Control MG	shMMP14 MG	shMMP14 MG +MMP14 rescue	marker

Source Data 3: Uncropped Blot of Extended Data Fig. 8g

BCAN+MAP2

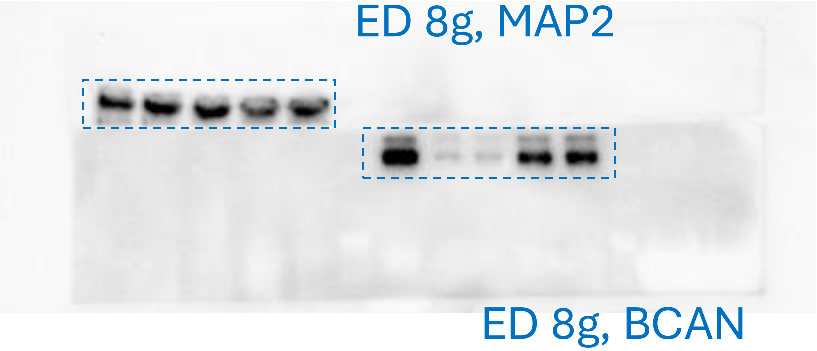

camera

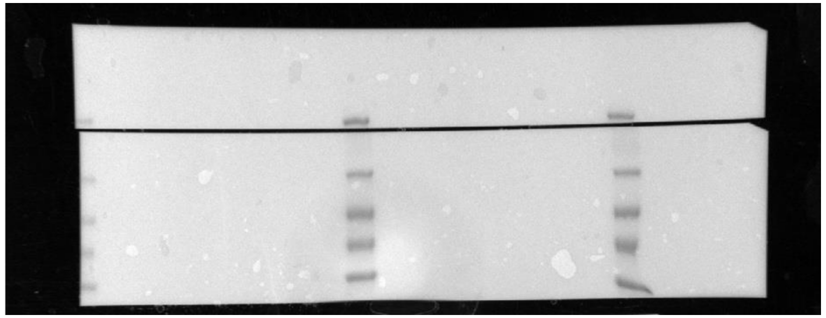

Merge

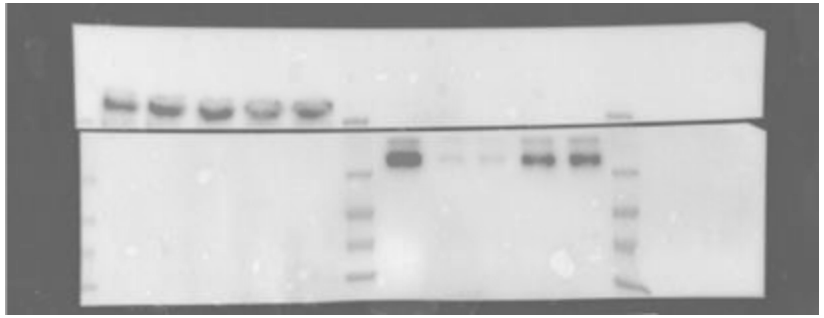

Sample information

	Cell lysate						Supernatant 10X					
marker	no MG	WT_MG	Control MG	shMMP14_1 MG	shMMP14_2 MG	marker	no MG	WT_MG	Control MG	shMMP14_1 MG	shMMP14_2 MG	marker
